# POGLUT2 and POGLUT3 *O*-glucosylate multiple EGF repeats in fibrillin-1, -2, and LTBP1 and promote secretion of fibrillin-1

**DOI:** 10.1016/j.jbc.2021.101055

**Published:** 2021-08-17

**Authors:** Daniel B. Williamson, Camron J. Sohn, Atsuko Ito, Robert S. Haltiwanger

**Affiliations:** 1 Complex Carbohydrate Research Center, University of Georgia, Athens, Georgia, USA; 2 Department of Biochemistry and Molecular Biology, University of Georgia, Athens, Georgia, USA

**Keywords:** glycosylation, extracellular matrix protein, mass spectrometry, glycoprotein secretion, protein folding, EGF repeats, *O*-glucose, ANOVA, analysis of variance, DMEM, Dulbecco’s modified Eagle’s medium, ECM, extracellular matrix, EGF, epidermal growth factor-like, EIC, extracted ion chromatogram, FBN1, fibrillin-1, FBN2, fibrillin-2, HEK293T, human embryonic kidney 293T, IgG, immunoglobulin G, LTBP1, latent transforming growth factor β-binding protein 1, MEM, minimal essential medium, MFS, Marfan syndrome, PBS, phosphate-buffered saline, PEI, polethylenimine, POGLUT2, protein *O*-glucosyltransferase 2, POGLUT3, protein *O*-glucosyltransferase 3, SD, standard deviation, TBS, Tris-buffered saline, TGF-β, transforming growth factor β

## Abstract

Fibrillin-1 (FBN1) is the major component of extracellular matrix microfibrils, which are required for proper development of elastic tissues, including the heart and lungs. Through protein–protein interactions with latent transforming growth factor (TGF) β-binding protein 1 (LTBP1), microfibrils regulate TGF-β signaling. Mutations within the 47 epidermal growth factor-like (EGF) repeats of FBN1 cause autosomal dominant disorders including Marfan Syndrome, which is characterized by disrupted TGF-β signaling. We recently identified two novel protein *O*-glucosyltransferases, Protein *O*-glucosyltransferase 2 (POGLUT2) and 3 (POGLUT3), that modify a small fraction of EGF repeats on Notch. Here, using mass spectral analysis, we show that POGLUT2 and POGLUT3 also modify over half of the EGF repeats on FBN1, fibrillin-2 (FBN2), and LTBP1. While most sites are modified by both enzymes, some sites show a preference for either POGLUT2 or POGLUT3. POGLUT2 and POGLUT3 are homologs of POGLUT1, which stabilizes Notch proteins by addition of *O*-glucose to Notch EGF repeats. Like POGLUT1, POGLUT2 and 3 can discern a folded *versus* unfolded EGF repeat, suggesting POGLUT2 and 3 are involved in a protein folding pathway. *In vitro* secretion assays using the N-terminal portion of recombinant FBN1 revealed reduced FBN1 secretion in *POGLUT2* knockout, *POGLUT3* knockout, and *POGLUT2* and *3* double-knockout HEK293T cells compared with wild type. These results illustrate that POGLUT2 and 3 function together to *O*-glucosylate protein substrates and that these modifications play a role in the secretion of substrate proteins. It will be interesting to see how disease variants in these proteins affect their *O*-glucosylation.

Fibrillins are extracellular matrix (ECM) glycoproteins that form microfibrils, which act as a scaffold for elastin deposition (1). Depending on developmental stage, microfibrils consist of fibrillin-1 (FBN1) and/or fibrillin-2 (FBN2) and are commonly found in connective tissues such as the heart, lungs, and skin (1, 2). Through interactions with latent TGFβ-binding proteins (LTBPs) such as LTBP1, microfibrils also act as a reservoir of TGFβ (3, 4, 5). LTBP1 covalently binds the small latent complex (SLC) consisting of TGFβ bound to its latency-associated peptide (LAP). This interaction of LTBP1, LAP, and TGFβ comprises the large latent complex (LLC) and is necessary for secretion of TGFβ (6, 7). Previous studies have confirmed interactions between LTBP1 and FBN1 (4, 8, 9). FBN1 microfibrils bind the LLC, leading to a large reservoir of latent TGFβ in the ECM (10, 11). Once sequestered in the ECM, TGFβ can be activated through interactions between LTBP1 and integrins (12). Fibrillins and LTBPs comprise a protein superfamily, and mutations within this protein family cause various human developmental disorders including Marfan syndrome (MFS), which has been linked to dysregulated TGFβ signaling (13, 14). Fibrillins and LTBPs have similar domain structures consisting heavily of sequential calcium-binding epidermal growth factor repeats (cbEGFs). Each EGF repeat contains six conserved cysteine residues that form three disulfide bonds in a conserved pattern. A large percentage of EGF repeats in these proteins contain a consensus sequence for modification with an *O*-glucose sugar by two recently described enzymes: POGLUT2 and POGLUT3 (15).

Decades of research have been performed analyzing *O*-glycosylation of EGF repeats and the significance of the *O*-glycans. This research has been dominated by investigations of the Notch receptors (16, 17, 18, 19), which structurally resemble fibrillins and LTBPs in that they have extracellular domains with as many as 36 tandem EGF repeats. Notch EGF repeats are heavily *O*-glycosylated by three enzymes: Protein *O*-Glucosyltransferase 1 (POGLUT1), Protein *O*-Fucosyltransferase 1 (POFUT1), and EGF Domain-Specific *O*-Linked *N*-Acetylglucosamine Transferase (EOGT) (Fig. 1*A*) (20, 21, 22, 23, 24, 25, 26, 27). Elimination of either *Pofut1* or *Poglut1* in mice results in embryonic lethality with Notch-associated phenotypes (28, 29), illustrating the significance *O*-glycans have on Notch function.

**Figure 1:**
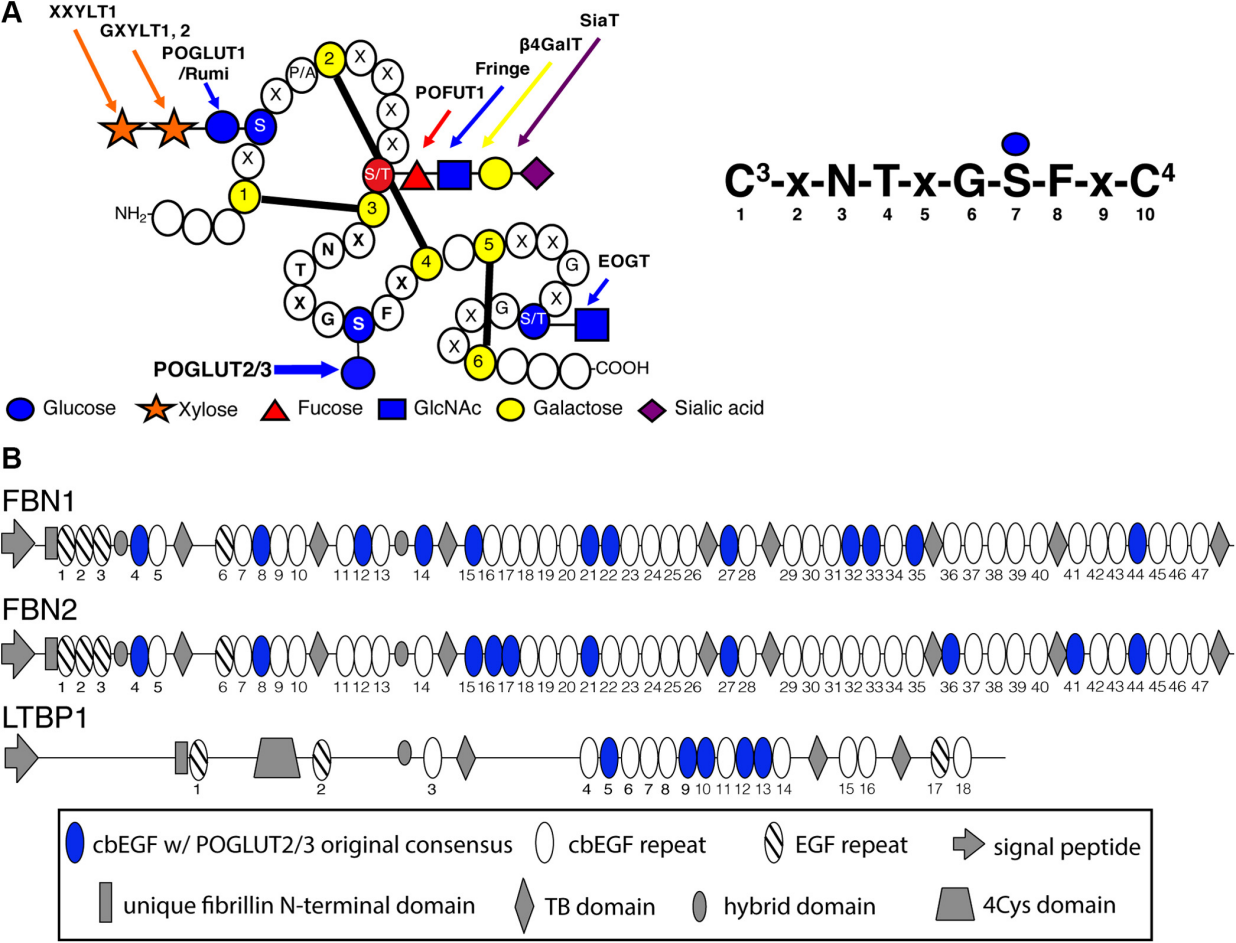
**Predicted sites of modification by POGLUT2 and/or POGLUT3 on EGF repeats of human FBN1, FBN2, and LTBP1 based on original consensus sequence.***A*, *left*, a *cartoon* of an EGF repeat showing modification sites of several *O*-glycosyltransferases. POGLUT2 and 3 modify EGF repeats at a site distinct from POGLUT1, POFUT1, and EOGT. *Yellow*, cysteines. Adapted with permission from (15). *A*, *right*, the original consensus sequence with positions between cysteines 3 and 4 numbered. *B*, domain maps of human FBN1, FBN2, and LTBP1. *Blue ovals*, EGF repeat with original POGLUT2 and 3 consensus sequence, C^3^-X-N-T-X-G-S-F-X-C^4^. *White ovals*, EGF repeat without original POGLUT2 and 3 consensus sequence.

POGLUT2 and 3 were identified as two *O*-glucosyltransferases that modify Notch EGF repeats between cysteines 3 and 4 of the EGFs with the consensus sequence C^3^-x-N-T-x-G-S-F-x-C^4^, in which an *O*-glucose is added to serine (15) (Fig. 1*A*). This site of modification is between cysteines 3 and 4 of an EGF repeat, distinct from POGLUT1 modifications that occur between cysteines 1 and 2. Only two sites on Notch proteins were modified by POGLUT 2 and 3 (15). FBN1 and -2 consist of 47 EGFs repeats, and LTBP1 has 18 EGF repeats. Using the original POGLUT2 and 3 consensus sequence, human FBN1, FBN2, and LTBP1 are predicted to have 12, 10, and 5 EGF repeats modified by POGLUT2 and 3, respectively (Fig. 1*B*). The small number of POGLUT2 and 3 sites on Notch receptors compared with the number of predicted sites on fibrillins and LTBP suggests POGLUT2 and 3 may have a more significant role in the function of these proteins compared with Notch. It has previously been shown that posttranslational modifications exist on several serine residues located between FBN1 EGFs29 to 35 (30), but neither the modifications nor the enzymes responsible were identified. Interestingly, these sites match the POGLUT2 and 3 consensus sequence.

The consensus sequence for POGLUT2 and 3 mediated *O*-glucosylation overlaps with the consensus sequence for β-hydroxylation, C^3^-x-(ND)-x-x-x-x-(FY)-x-C^4^, which has been linked to calcium binding that is essential for the structure and function of FBN1 (30, 31, 32, 33, 34, 35). Our work presents the first demonstration that FBN1, FBN2, and LTBP1 are extensively modified by *O*-glucose residues at high stoichiometry, added by POGLUT2 and/or POGLUT3. Semiquantitative mass spectral analysis was performed to determine sites of *O*-glucosylation and β-hydroxylation as well as the efficiency of modification at each site. Our results show that approximately half of the EGFs in these proteins are modified by POGLUT2 and/or 3, indicating that our original consensus sequence was too narrow. Additionally, we have identified site-specific preferences between POGLUT2 and 3 and demonstrated that POGLUT2 and 3 are possible regulators of protein folding and secretion.

## Results

### Endogenous human FBN1 is extensively *O*-glucosylated at high stoichiometry

**Figure 2:**
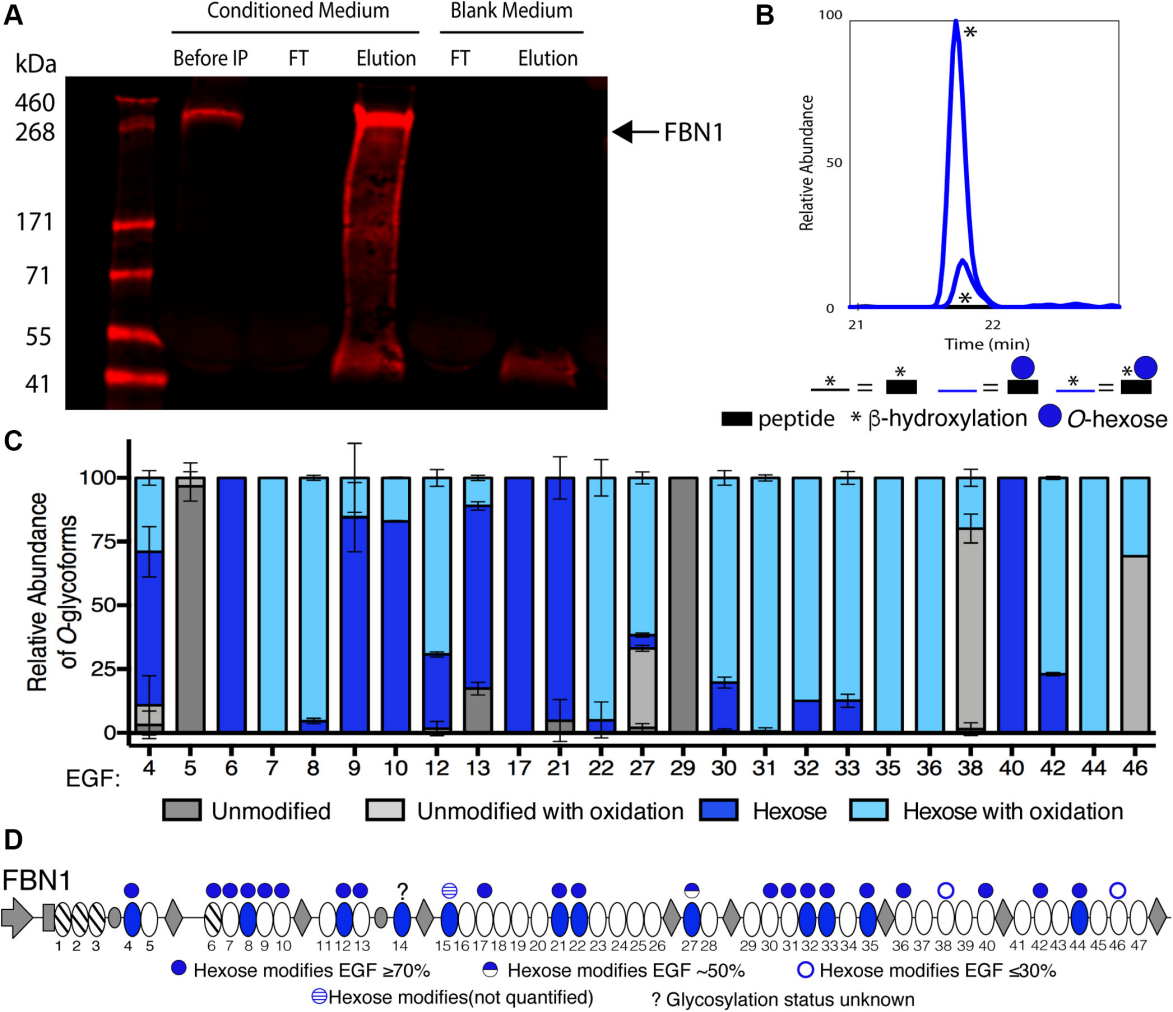
**Endogenous FBN1 is modified at high stoichiometry with hexose at more than the predicted number of EGF repeats.***A*, western blot illustrating successful immunoprecipitation of endogenous FBN1 from conditioned medium of human dermal fibroblasts. *Before* IP, fibroblast conditioned medium before immunoprecipitation. *FT*, flow-through after antibody application. *Elution*, human FBN1 eluted off magnetic beads. *Red channel*, anti-proline-rich N-terminal region of human FBN1. *Arrow* marks migration position of FBN1. *B*, representative EIC of the peptide from FBN1 EGF33 modified by a hexose at high stoichiometry. EICs were generated by searching for m/z values of glycoforms of the tryptic peptide from EGF33, R.CVNTYGSYECK.C. Mass spectral data in Table S5 and Fig. S22. *C*, relative quantification of hexose modification and β-hydroxylation on peptides derived from the indicated FBN1 EGFs averaged from three biological replicates. Error bars show ±SD. Mass spectral data in Table S5 and Figs. S3–S29. *D*, domain map of FBN1 illustrating predicted (based on original consensus sequence C^3^-X-N-T-X-G-S-F-X-C^4^) and confirmed sites of modification by hexose. *Blue oval*, EGF with original POGLUT2 and 3 consensus sequences. *Blue circle*, EGF confirmed modified with hexose. ?, glycosylation status is unknown due to lack of sequence coverage from mass spectral data. EGF15 is modified, but quantification was not possible because the unmodified peptide m/z is too small for the mass spectrometer to detect.

To confirm whether human FBN1 is modified with *O*-glucose, we first analyzed endogenous human FBN1 secreted from human dermal fibroblasts. Endogenous FBN1 was purified by immunoprecipitation of conditioned medium from 7-day postconfluent fibroblasts using a well-characterized polyclonal antibody that recognizes the unique N-terminal proline-rich domain of human FBN1 (36). A representative western blot illustrating FBN1 purification from conditioned media is shown in Figure 2*A*.

An established protocol for preparing purified proteins and digesting with trypsin for mass spectral analysis was used to identify *O*-glucosylation sites on purified human FBN1 (15). We obtained peptide matches covering greater than 70% of endogenous full-length FBN1. All peptides were identified using Byonic (Table S5 and Figs. S3–S29). For quantification of *O*-glycoforms, extracted ion chromatograms (EICs) were generated for each peptide modified with *O*-Hexose (Fig. 2*B*). Four glycoforms were searched based on mass to charge ratio (*m/z*) for each EIC: unmodified peptide, unmodified peptide plus β-hydroxylation, peptide plus *O*-hexose, and peptide plus *O*-hexose and β-hydroxylation. The relative abundance of each glycoform of a specific peptide was calculated and plotted as a percentage of the total abundance (Fig. 2*C*).

Based on the original consensus sequence, 11 of 12 predicted sites were modified with *O*-hexose at high stoichiometry. EGF14 did not have sequence coverage, so it was not analyzed. Further analysis led to the identification of 13 additional EGFs modified with *O*-hexose, most of which were modified at high stoichiometry. Some EGFs had lower amounts of *O*-hexose modification (EGF38 and 46). We also identified EGFs that had a serine at position 7 of the POGLUT2/3 consensus sequence (Fig. 1*A*) but were unmodified (EGF5 and 29). The 13 additional modification sites included amino acids that were not identified in the original POGLUT2 and 3 consensus sequence, broadening the consensus sequence for POGLUT2 and 3 by allowing for a tyrosine at position 8 of the consensus sequence. In total, over half of the 47 EGF repeats of endogenous human FBN1 were modified by POGLUT2 and 3 (Fig. 2*D*). Many of the cbEGFs are also modified more than 50% by β-hydroxylation, with some notable exceptions (EGFs 4, 6, 9, 10, 13, 17, 21, and 40; Fig. 2*C*).

### POGLUT2 and/or 3 are responsible for addition of *O*-glucose to FBN1

**Figure 3:**
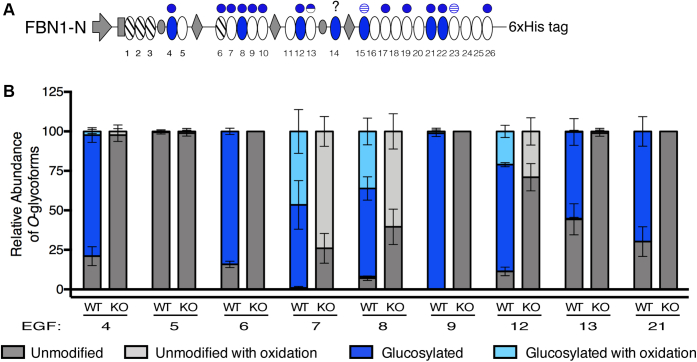
**POGLUT2 and 3 extensively *O*-glucosylate EGF repeats on recombinant human FBN1 EGFs 1 to 26 (FBN1-N) expressed in HEK293T cells.***A*, schematic of recombinant protein expressed in HEK293T cells as in Figures 1*B* and 2*D*. *B*, relative quantification of major *O*-glycoforms and β-hydroxylation of peptides derived from EGFs of FBN1-N overexpressed in wild-type or *POGLUT2/3* double knockout HEK293T cells. Only EGFs with at least two replicate values are plotted in the bar graph. Error bars show ± SD. Mass spectral data in Tables S6–S10 and Figs. S3–S17. *KO*, *POGLUT2/3* double-knockout cells; *WT*, wild type.

To confirm that POGLUT2 and 3 are indeed responsible for the modifications found on FBN1, and that these modifications are *O*-glucose, transient transfections with constructs encoding His-tagged N- and C-terminal FBN1 fragments (37) were performed in wild-type and *POGLUT2/3* double-knockout HEK293T cells as previously described (15). The FBN1 N-terminal protein fragment (FBN1-N) is illustrated in Figure 3*A* with predicted and confirmed sites of POGLUT2 and 3 modification identified. Recombinant FBN1-N was purified from conditioned media over a Ni-NTA column, digested, and prepared for mass spectral analysis (Fig. S1). Fifteen of the 26 EGFs were confirmed to be modified at similar stoichiometries to those seen in endogenous FBN1, confirming that HEK293T cells are a good system to monitor this form of modification. In addition to the modified EGFs identified on the N-terminal region of endogenous FBN1 (Fig. 2*D*), three additional modified EGFs were identified on recombinant FBN1-N (EGF19, 23, 26, Fig. 3*A*). These additional sites were identified due to a higher protein sequence coverage in our mass spectral analysis of overexpressed FBN1-N *versus* endogenous FBN1. POGLUT2 and 3 mediated modifications were lost when FBN1-N was expressed in *POGLUT2* and *3* double-knockout HEK293T cells, confirming that these two enzymes are responsible for *O*-glucosylation of FBN1 and that the modifications are *O*-glucose (Fig. 3*B*). Interestingly, the level of β-hydroxylation is lower on recombinant FBN1-N compared with endogenous FBN1 (compare EGFs 7, 8, and 12 in Figs. 2*C* and 3*B*), suggesting that β-hydroxylation is less efficient in HEK293T cells than in dermal fibroblasts. All peptides were identified using Byonic (Tables S6–S10 and Figs. S3–S17).

The C-terminal half of FBN1 (FBN1-C) was also expressed in wild-type and *POGLUT2/3* double-knockout HEK293T cells, purified from media (Fig. S1), and analyzed by mass spectrometry. The same POGLUT2 and 3 modified EGFs were identified between the C-terminal region of endogenous FBN1 and recombinant FBN1-C; however, the POGLUT2 and 3 modification efficiency deviated from what was observed with endogenous FBN1. It is possible there was a folding defect in FBN1-C protein, leading to lower stoichiometry of POGLUT2 and 3 modified peptides compared with endogenous FBN1. Due to this, we did not quantify the efficiency of modification for *O*-glucosylation by POGLUT2 and 3 on FBN1-C. All *O*-glucose modifications on FBN1-C were lost when expressed in the *POGLUT2/3* double-knockout cells. POGLUT2 and 3 modified peptides of recombinant FBN1-C can be found in Tables S11 and S12 (Figs. S18–S29).

### FBN2 and LTBP1 are *O*-glucosylated at multiple sites by POGLUT2 and 3 in HEK293T cells

**Figure 4:**
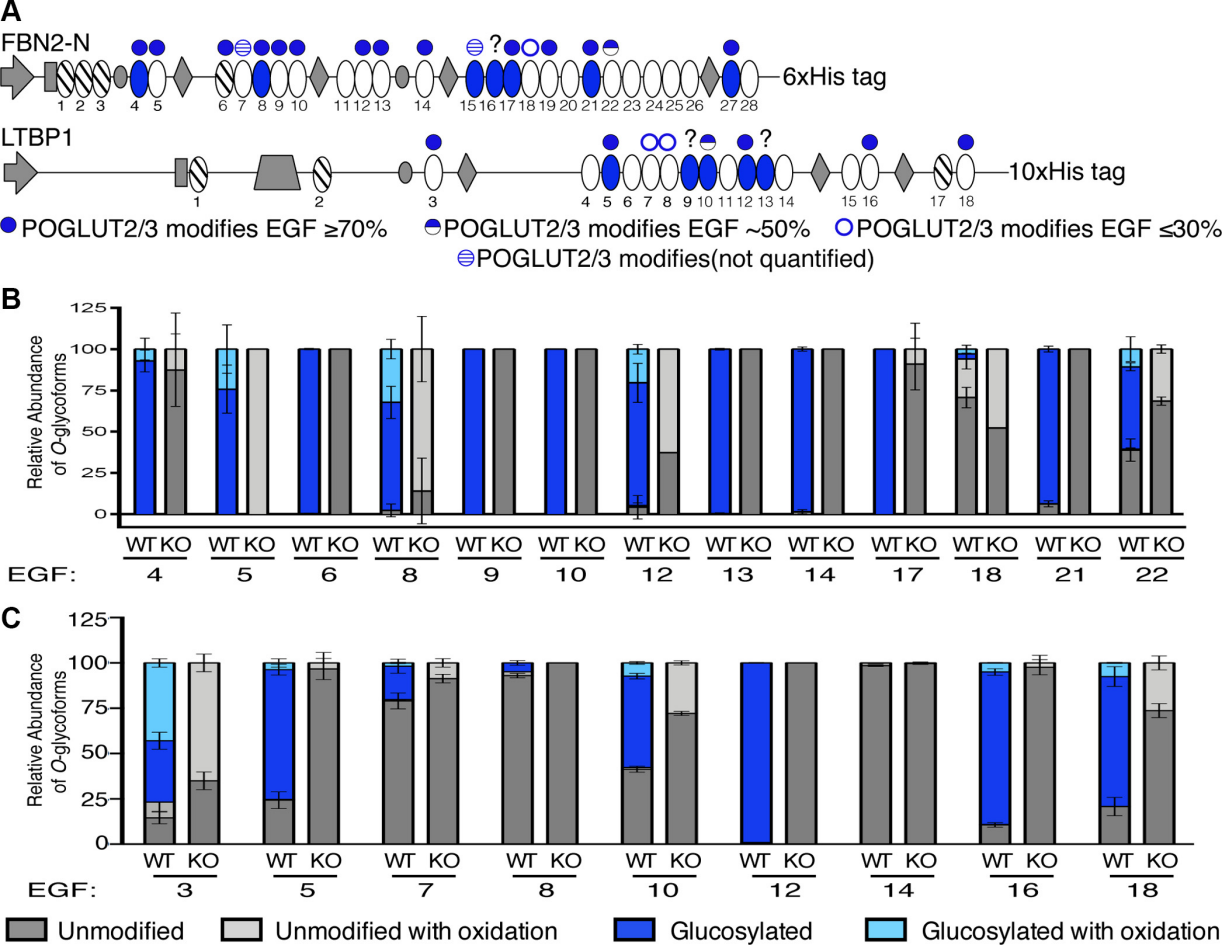
**POGLUT2 and 3 extensively *O*-glucosylate EGF repeats on recombinant human FBN2 EGFs 1 to 28 (FBN2-N) and LTBP1 in HEK293T cells.***A*, schematic of recombinant FBN2-N and LTBP1 expressed HEK293T cells as in Figures 1*B* and 2*D*. Relative quantification of major *O*-glycoforms and β-hydroxylation of peptides derived from EGFs of FBN2-N (*B*) and LTBP1 (*C*) overexpressed in wild-type and *POGLUT2/3* double-knockout HEK 293T cells. Only EGFs with at least two replicate values are plotted in the bar graph. Error bars show ± SD. Mass spectral data for FNB2-N are in Tables S13–S15 and Figs. S30–S46, and for LTBP1 are in Tables S16–S19 and Figs. S57–S64. *KO*, *POGLUT2/3* double-knockout cells; *WT*, wild type.

A FBN2 N-terminal fragment (FBN2-N) (38) and full-length LTBP1 (3) were expressed in wild-type and *POGLUT2/3* double-knockout HEK293T cells (Fig. 4*A*). Each recombinant protein was purified using a Ni-NTA column from conditioned medium (Fig. S1), digested, and analyzed by mass spectrometry. As with FBN1, a greater number of EGFs than predicted with the original consensus sequence were modified by POGLUT2 and/or 3 for both proteins. For FBN2-N, 17 out of 28 total EGFs were *O*-glucosylated, and for LTBP1, eight out of 18 total EGFs were *O*-glucosylated (Fig. 4*A*). The majority of these POGLUT2 and 3 modifications were at high stoichiometry (Fig. 4, *B* and *C*). FBN2 EGF18 is an interesting site that has very low levels of POGLUT2 and/or 3 modification. This site almost matches the original POGLUT2 and 3 consensus sequence, but instead of a serine at position 7 of the consensus sequence, there is a threonine. This suggests that POGLUT2 and 3 can modify a threonine, but serine is highly preferred. All *O*-glucose modifications were lost when FBN2-N or LTBP1 were expressed in *POGLUT2/3* double-knockout HEK293T cells, confirming that POGLUT2 and 3 are responsible for *O*-glucosylating FBN2 and LTBP1 (Fig. 4, *B* and *C*). Peptides identified using Byonic for FBN2-N can be found in Tables S13–S15 (Figs. S30–S46) and those for LTBP1 in Tables S16–S19 (Figs. S57–S64).

FBN2-C (38) was also expressed in wild-type and *POGLUT2/3* double-knockout cells, and the resulting peptides identified for FBN2-C can be found in Tables S20 and S21 (Figs. S47–S56). As with FBN1-C, most of the EGFs were modified 50% or less by POGLUT2 and 3. As previously mentioned, FBN1-C recombinant data did not correspond with endogenous FBN1 mass spectral data. Due to this, we did not quantify stoichiometry at each modified EGF, but instead only identified FBN2-C EGFs that were modified by POGLUT2 and/or 3. All *O*-glucose residues were lost when FBN2-C was expressed in *POGLUT2/3* double-knockout cells (Table S21).

### POGLUT2 and POGLUT3 modify EGF repeats with the consensus sequence C^3^-x-N-T-x-G-S-(FY)-x-C^4^

**Figure 5:**
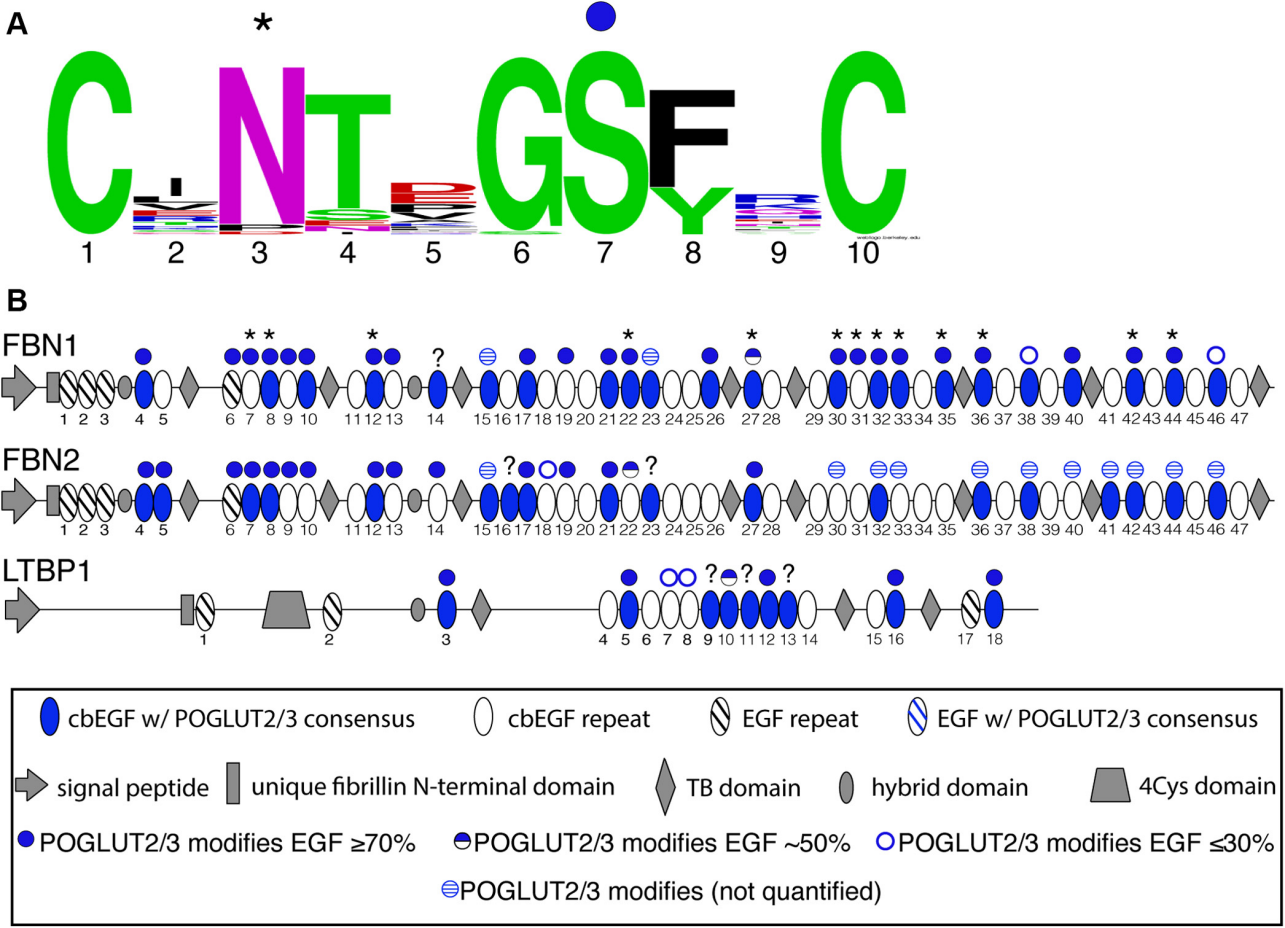
**Summary of POGLUT2 and 3 sites mapped on endogenous FBN1 from dermal fibroblasts and recombinant FBN1, FBN2, and LTBP1 expressed in HEK293T cells.***A*, WebLogo summary of all identified POGLUT2 and 3 modification sites. *Numbers* below amino acid indicate position within consensus sequence between cysteines 3 and 4 of an EGF repeat. ∗, predicted site for β-hydroxylation. *Blue circle*, *O*-glucose modified site. *B*, domain maps of FBN1, FBN2, and LTBP1 illustrating predicted and confirmed sites of POGLUT2 and 3 modification. *Blue ovals*, EGF with revised consensus sequence, C^3^-x-N-T-x-G-S-(FY)-x-C^4^. ∗, FBN1 EGFs that are *O*-glucosylated by POGLUT2/3 and β-hydroxylated more than 50%. ?, glycosylation status unknown due to lack of sequence coverage from mass spectral data.

After compiling all confirmed POGLUT2 and 3 modification sites on NOTCH1, NOTCH3, FBN1, FBN2, and LTBP1, we generated a WebLogo summarizing sequences between cysteines 3 and 4 of all modified sites (Fig. 5*A*). The most notable difference compared with the original consensus sequence is the addition of a tyrosine at the eighth position in the sequence, C^3^-x-N-T-x-G-S-(FY)-x-C^4^. A summary of the predicted POGLUT2 and 3 *O*-glucosylation sites using the highly conserved residues of the new consensus sequence and confirmed modification sites on FBN1, -2, and LTBP1 is shown in Figure 5*B*. For all three proteins, approximately half of their EGF repeats are *O*-glucosylated by POGLUT2 and 3.

While all sites with the C^3^-x-N-T-x-G-S-(FY)-x-C^4^ sequence were at least partially modified, other sites contain nonconserved residues at the third, fourth, and sixth position (Fig. 5*A*). For example, at the third position where asparagine is mostly conserved, FBN1 EGF6 has a proline and FBN1 EGF31 has an aspartic acid. Both of these EGFs were modified at high stoichiometry by POGLUT2 and/or 3. At the fourth position where threonine is mostly conserved, we also observed EGFs with asparagine, glutamate, serine, methionine, leucine, isoleucine, and valine. *O*-glucosylation generally occurs at high stoichiometry with asparagine, glutamine, or serine at the fourth position. A significant decrease or no modification at all was observed when methionine, leucine, isoleucine, or valine was at the fourth position (Table S2). This suggests that hydrophobic amino acids at this position are not preferred by POGLUT2 and 3 for *O*-glucosylation of an EGF repeat. A serine in position 4 is usually highly modified (FBN1 EGF13; FBN2 EGF10, 14, 19), but LTBP1 EGF7 is poorly modified, suggesting that other factors in LTBP1 are reducing the efficiency of modification at EGF7. At the sixth position of the consensus sequence where glycine is mostly conserved, FBN1 EGF5 and FBN2 EGF6 had a serine. Interestingly, FBN1 EGF5 was modified at very low stoichiometry (<1%), but FBN2 EGF6 was modified at high stoichiometry, again indicating that additional unknown factors are contributing to efficiency of modification at individual EGFs.

**Figure 6:**
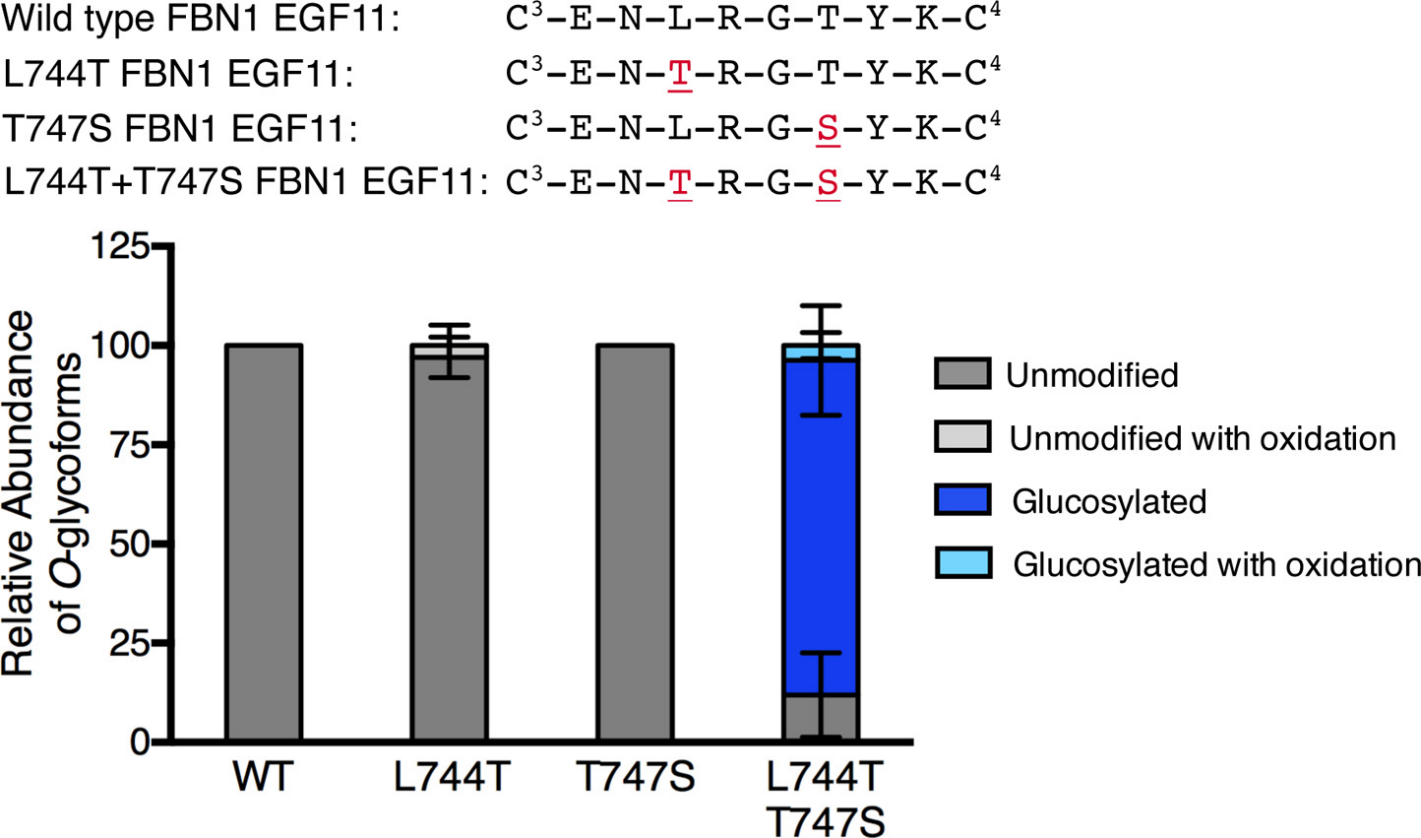
**POGLUT2 and 3 consensus sequence can be used to convert an unmodified EGF to a POGLUT2/3 modified EGF.** Wild-type and mutant amino acid sequence between cysteines 3 and 4 of FBN1 EGF11. *Red underlined*, mutated sites. Relative quantification of *O*-glycoforms and β-hydroxylation of peptides from wild type and mutant FBN1 EGF11. Error bars show ± SD. Mass spectral data are in Tables S22–S25.

To further analyze the POGLUT2 and 3 consensus sequence, we attempted to convert an unmodified EGF to a POGLUT2 and/or 3 modified EGF. We selected FBN1 EGF11, which has the sequence C^3^-x-N-L-x-G-T-Y-x-C^4^. Mass spectral analysis confirmed this site is not modified by POGLUT2 or 3 in WT cells (Fig. 6). This site may be unmodified due to the hydrophobic leucine residue at position 4 and/or because threonine is at position 7 instead of serine. Threonine and serine differ by a single methyl group. *O*-glycosyltransferases such as POFUT1 and EOGT have been shown to modify both a serine and threonine (20, 21), while POGLUT1 only modifies serine (20, 39). With site-directed mutagenesis we generated the following three mutants using FBN1-N plasmid: L744T (position 4), T747S (position 7, potential site of modification), and double-mutant L744T/T474S (Fig. 6). Each mutant was expressed in wild-type HEK293T cells and purified protein was analyzed by mass spectrometry. FBN1 EGF11 remained unmodified in the single mutants but was modified at high stoichiometry in the L744T/T747S double mutant (Fig. 6). This further supports POGLUT2 and 3 do not prefer a hydrophobic residue at position 4 of the consensus sequence, and it also demonstrates that serine is preferred over threonine at position 7. The ability to convert an unmodified EGF to a POGLUT2 and/or 3 modified EGF further supports the importance of the highly conserved residues in the consensus sequence for POGLUT2 and 3. Peptides identified by Byonic for FBN1 EGF11 are in Tables S22–S25.

### POGLUT2 and POGLUT3 display some site-specific differences

**Figure 7:**
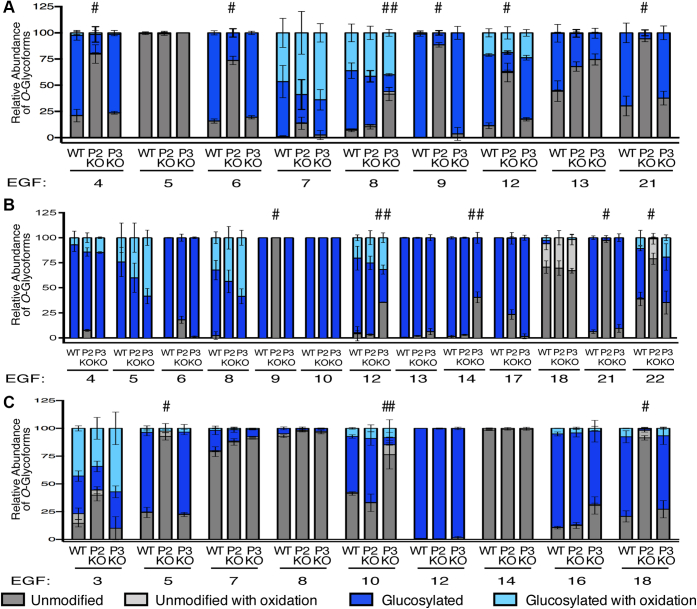
**POGLUT2 and POGLUT3 exhibit site specificity on EGF repeats of recombinant FBN1-N, FBN2-N, and LTBP1.** Relative quantification of major *O*-glycoforms and β-hydroxylation of peptides from EGF repeats. *A*, FBN1-N. Mass spectral data in Tables S26 and S27. *B*, FBN2-N. Mass spectral data in Tables S28 and S29. *C*, LTBP1. Mass spectral data in Tables S30–S33. Proteins were overexpressed in wild-type (WT), *POGLUT2* knockout (P2 KO), or *POGLUT3* knockout (P3 KO) HEK293T cells. #, *O*-glucosylation decreased in *POGLUT2* knockout cells by more than 30%. ##, *O*-glucosylation decreased in *POGLUT3* knockout cells by more than 30%. Only EGFs with at least two replicate values are plotted in the bar graph. Error bars show ± SD.

**Figure 8:**
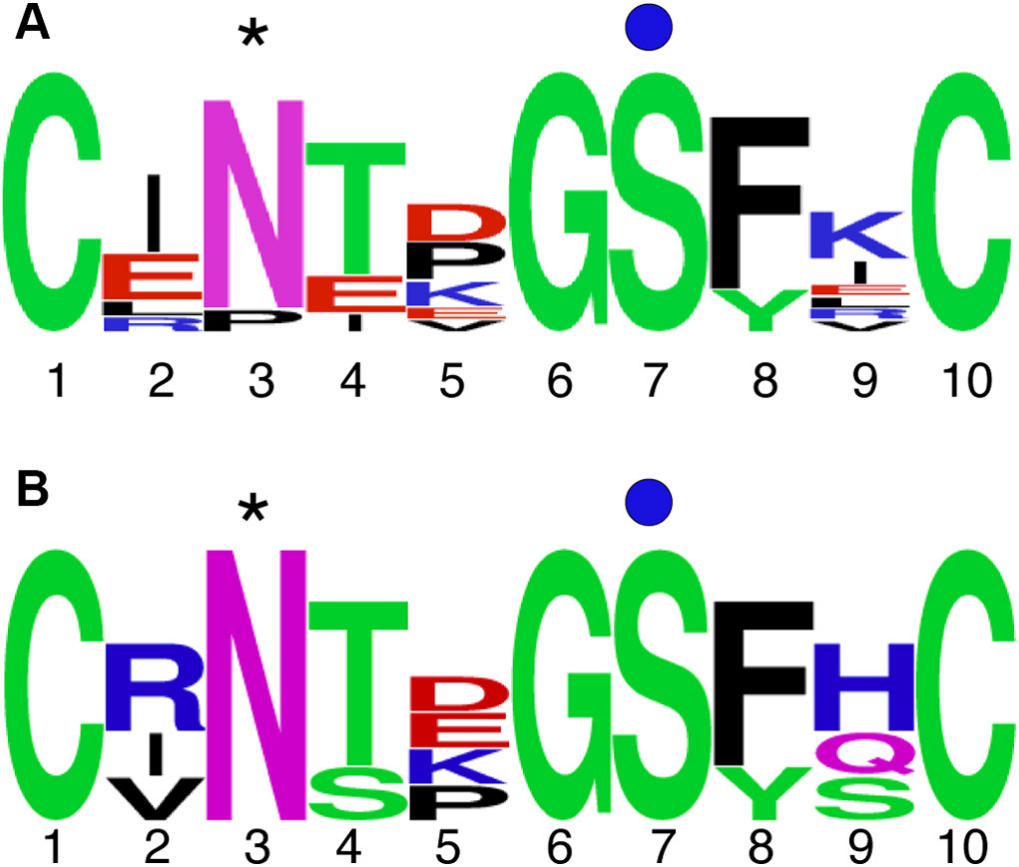
**POGLUT2 *versus* POGLUT3 consensus sequence.***A*, WebLogo summarizing sequences between cysteines 3 and 4 of the EGF repeat preferred by POGLUT2 based on mass spectral analysis of FBN1-N, FBN2-N, and LTBP1. *B*, WebLogo summarizing sequences preferred by POGLUT3 based on mass spectral analysis of FBN1-N, FBN2-N, and LTBP1. ∗, predicted site for β-hydroxylation. *Blue circle*, *O*-glucose modification site.

Our previous studies have indicated that POGLUT2 and 3 show a unique preference for different EGFs of Notch proteins (15). To further investigate this site specificity, FBN1-N, FBN2-N, and LTBP1 were expressed in *POGLUT2* single-knockout and *POGLUT3* single-knockout cells and mass spectral analysis was performed (Tables S26–S33). These results were compared to wild-type data to identify sites where *O*-glucosylation was reduced by either knockout condition (Fig. 7). *O*-glucosylation needed to be reduced by 30% or more compared with wild type to be considered significant. This cutoff value was used to account for sample variation and mass spectrometer conditions that typically affect mass spectral quantification in the 10 to 20% range. Several EGFs were identified as POGLUT2 or POGLUT3 preferred sites on FBN1-N (Fig. 7*A*), FBN2-N (Fig. 7*B*), and LTBP1 (Fig. 7*C*). For example, FBN1 EGF9 is *O*-glucosylated at high stoichiometry in both wild-type and *POGLUT3* knockout cells, but in *POGLUT2* knockout cells the *O*-glucose modification is almost completely lost (Fig. 7*A*). Since *O*-glucosylation is significantly reduced only when *POGLUT2* is knocked out, this suggests FBN1 EGF9 cannot be modified efficiently by POGLUT3 and is a POGLUT2 preferred EGF repeat. Conversely, LTBP1 EGF 10 is approximately 50% modified in wild-type and *POGLUT2* knockout cells, but in *POGLUT3* knockout cells the *O*-glucose modification is almost completely lost (Fig. 7*C*). This suggests that LTBP1 EGF10 is a POGLUT3 preferred EGF repeat since *O*-glucosylation is significantly reduced only when *POGLUT3* is knocked out. Some EGF repeats showed no preference by either enzyme. FBN2 EGF10 and 13 maintained *O*-glucosylation at high stoichiometry regardless of knockout condition (Fig. 7*B*). Ten POGLUT2 preferred EGFs were identified: FBN1 EGFs 4, 6, 9, 12, 21; FBN2 EGFs 9, 21, 22; and LTBP1 EGFs 5, and 18 (Table S3). Four POGLUT3 preferred sites were identified: FBN1 EGF8; FBN2 EGF 12 and 14; and LTBP1 EGF 10 (Table S3). Weblogo summaries of the sites modified by each enzyme are shown in Figure 8. There are no significant differences between the two consensus sequences, indicating that factors other than the amino acid sequence between cysteines 3 and 4 of an EGF repeat may contribute to site specificity. Overall, these data support our previous results (15) that while partially redundant, there is site specificity between POGLUT2 and 3 and that these enzymes work in a collaborative fashion to ensure *O*-glycosylation of substrate proteins.

**Figure 9:**
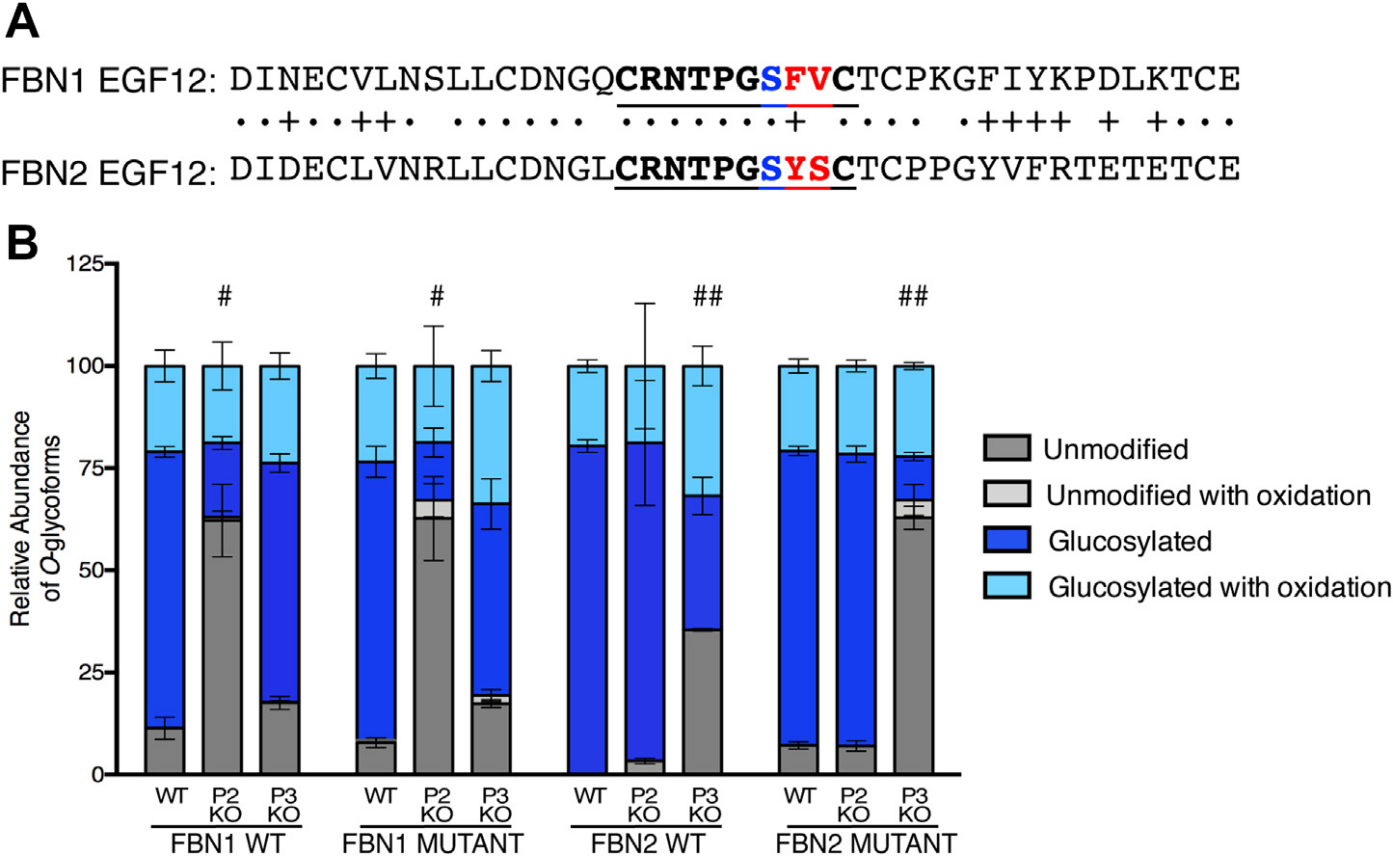
**POGLUT2 and POGLUT3 site specificity is not strictly defined by amino acid sequence between cysteines 3 and 4 of EGF repeats.***A*, protein sequence alignment of FBN1 EGF12 and FBN2 EGF12 (NCBI blastp). •, Identical amino acid. +, Accepted amino acid substitutions due to similar amino acid properties. *Bold*, POGLUT2/3 consensus sequence. *Blue*, modified residue. *Red*, amino acids that were swapped between wild type FBN1 and FBN2. *B*, relative quantification of major *O*-glycoforms and β-hydroxylation of peptides from EGF12 for WT FBN1-N, WT FBN2-N, and FBN1-N EGF12 mutant overexpressed in wild-type, *POGLUT2* knockout, or *POGLUT3* knockout HEK 293T cells. Error bars show ±SD. Mass spectral data in Tables S34–S39. #, EGF *O*-glucosylation decreased in *POGLUT2* knockout cells by more than 30%. ##, EGF *O*-glucosylation decreased in *POGLUT3* knockout cells by more than 30%.

Our site-mapping data showed the POGLUT2 and 3 consensus sequence can be used to accurately predict *O*-glucosylation sites, but we wanted to directly analyze whether changes in any position between cysteines 3 and 4 can drive site specificity between POGLUT2 and 3. For this study, FBN1 EGF12 and FBN2 EGF12 were selected. FBN1 EGF12 is POGLUT2 preferred, and FBN2 EGF 12 is POGLUT3 preferred (Fig. 7, *A* and *B*). These EGF repeats were selected because they only differ by two amino acids between cysteines 3 and 4, and they are in the same relative position of each protein (Fig. 9*A*, *red*). We found it interesting that two sites so similar exhibited variable enzyme specificity. F788Y/V789S double mutations were generated for FBN1-N, and Y832F/S833V double mutations were generated for FBN2-N. Each mutant was expressed in wild-type, *POGLUT2* knockout, and *POGLUT3* knockout cells and mass spectral analysis was performed on purified proteins (Tables S34–S39). Results were compared with wild-type FBN1-N and FBN2-N EGF12 data to determine changes in *O*-glucosylation by POGLUT2 and 3. No significant *O*-glucosylation differences were observed for either mutant compared with wild type, and only slight changes were seen in the *POGLUT2* or *POGLUT3* knockouts (Fig. 9*B*). These data strongly suggest that the site specificity of POGLUT2 and 3 is dictated by sequences outside of those between cysteines 3 and 4, and the context of an entire EGF repeat needs to be considered. We used Clustal Omega to align whole EGF sequences of POGLUT2 and POGLUT3 preferred EGFs (Fig. S2). No significant differences were identified when comparing whole EGF sequences. This suggests the primary sequence alone cannot be used to predict site specificity, but instead, other factors such as the position of an EGF in the context of protein structure need to be considered as well.

### Knockout of POGLUT2 and 3 reduces secretion of FBN1-N in HEK293T cells

**Figure 10:**
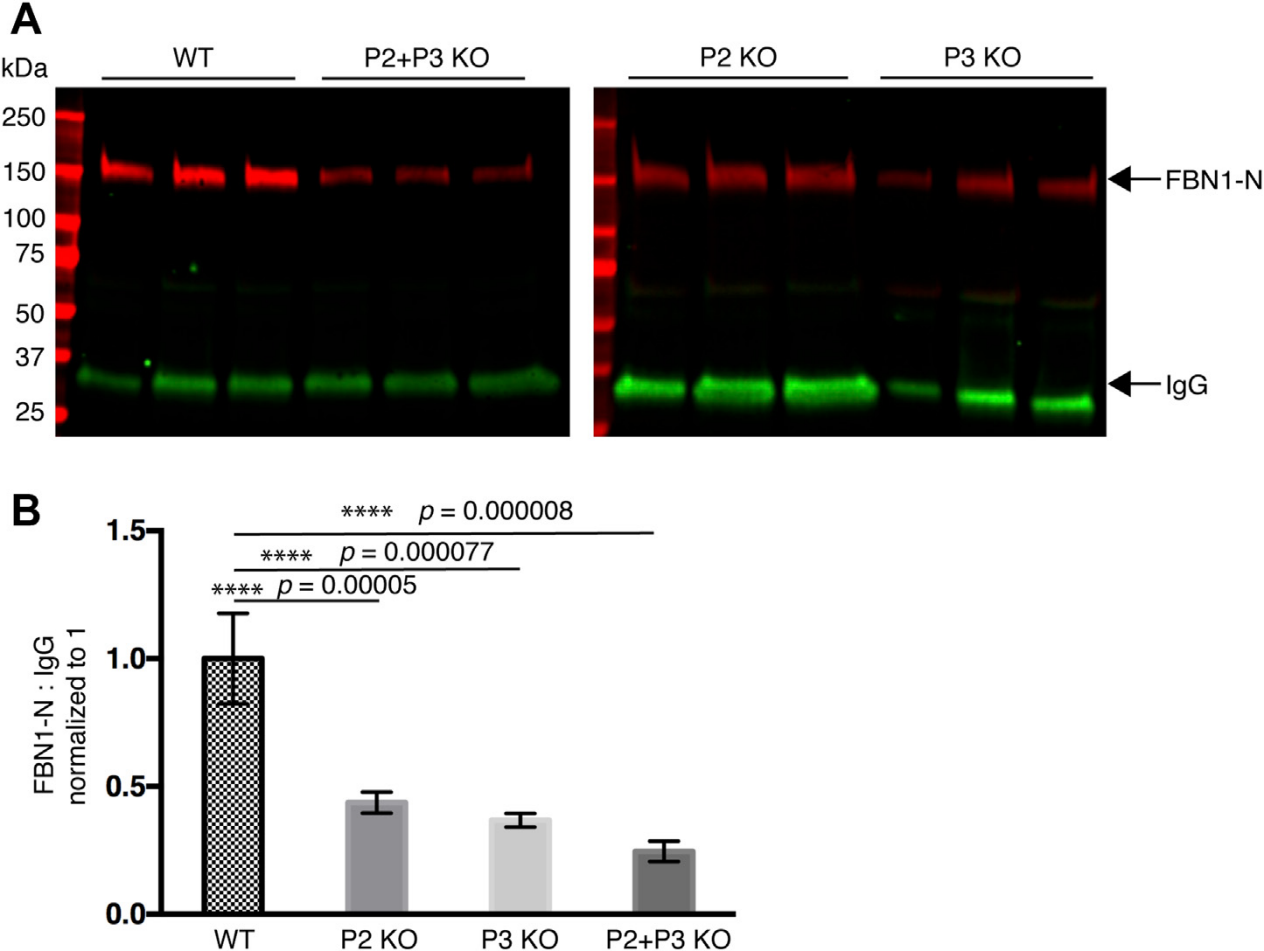
**Knockout of *POGLUT2* and/or *POGLUT3* leads to reduced secretion of recombinant human FBN1-N from HEK293T cells.***A*, secretion assay with hFBN1-N expressed in wild type, *POGLUT2* knockout, *POGLUT3* knockout, or *POGLUT2/3* double knockout HEK293T cells. IgG was cotransfected as a transfection and secretion control. *Red channel*, anti-His antibody. *Green channel*, anti-IgG antibody. *B*, quantification of western blot replicates. FBN1-N signal was normalized to IgG signal. One-way ANOVA in Excel was used to calculate significance. ∗∗∗∗*p* < 0.0001. Three biological replicates were performed, and three technical triplicates were performed on wild-type and double-knockout samples. Two biological replicates and three technical triplicates were performed on single-knockout samples. Error bars show ± SD.

We have previously shown that *O*-glycosylation by enzymes such as POFUT1 and POGLUT1 stabilizes EGF repeats and assists secretion of substrate proteins such as Notch (40). An established cell-based secretion assay protocol (41) was used to analyze whether *O*-glucosylation by POGLUT2 and 3 is necessary for efficient secretion of FBN1-N. The same FBN1-N construct used for mass spectral analysis was used for secretion assays. We attempted secretion assays using full-length protein, but it was difficult to consistently express for accurate quantification. Figure 10*A* is a representative western blot illustrating secretion of FBN1-N and coexpressed human IgG. Compared with wild-type HEK293T cells, secretion of FBN1-N was reduced approximately twofold in *POGLUT2* knockout or *POGLUT3* knockout cells, while secretion of FBN1-N was reduced approximately fourfold in *POGLUT2/3* double-knockout cells (Fig. 10*B*). Secretion of human IgG, a control protein without EGF repeats, was unchanged across all conditions. These data suggest that *O*-glucose addition by POGLUT2 and/or POGLUT3 is necessary for efficient secretion of FBN1-N.

## Discussion

Our work is the first to analyze *O*-glucosylation of FBN1, FBN2, and LTBP1. Through mass spectral analysis, we confirmed FBN1, FBN2, and LTBP1 are heavily modified with *O*-glucose by POGLUT2 and/or POGLUT3. We also identified site specificity between POGLUT2 and POGLUT3, which provides a potential rationale for why mammals possess two enzymes with functional redundancy. Finally, our data show that addition of *O*-glucose is necessary for proper secretion of FBN1-N, suggesting a biologically significant role for these enzymes.

In total, we mapped 62 POGLUT2 and 3 mediated *O*-glucosylation sites out of 112 EGF repeats of FBN1, -2, and LTBP1. The original POGLUT2 and 3 consensus sequence, C^3^-x-N-T-x-G-S-F-x-C^4^, predicted less than half these many *O*-glucose modification sites (compare Fig. 1 to Fig. 5) (15). Based on our analysis, most of the residues in the original consensus sequence were highly conserved, except that tyrosine was found in place of phenyalanine at nearly half of the modified sites. All sites containing the broadened consensus sequence, C^3^-x-N-T-x-G-S-(FY)-x-C^4^, were at least partially modified, suggesting that this sequence accurately predicts modification of an EGF repeat. Database searches with this sequence reveal that many other proteins also had increases in the predicted number of modification sites including hemicentin-1, fibulin-1, fibulin-2, and LTBP-4 (Table S4). The total number of human proteins predicted to be modified by POGLUT2 and 3 increased from 34 to 56, and the total number of predicted sites increased from 86 to 167 (Table S4). Nonetheless, our data show that residues other than N (position 3), T (position 4), or G (position 6) occur between cysteines 3 and 4 of modified sites, and there are likely additional factors outside of this sequence that play a role, indicating that more work needs to be done to define exactly what POGLUT2 and 3 recognize in an EGF repeat. To avoid missing additional proteins or sites modified by POGLUT2 and 3, a more general search string of C^3^-x-x-x-x-x-S-x-x-C^4^ can be used, although this sequence will also identify sites that are not modified.

Sequence logo graphs have previously been generated using over 1000 human EGF-like domain sequences found in the PROSITE database (42). Asparagine at position 3, glycine at position 6, serine or glycine at position 7, and tyrosine or phenylalanine at position 8 are the most commonly observed amino acids between cysteines 3 and 4 (42). Interestingly, this is very similar to the POGLUT2 and 3 consensus sequence. This suggests that all amino acids within the POGLUT2 and 3 consensus sequence may not be required for *O*-glucosylation, but instead, some of these amino acids exist within the consensus sequence simply because they are common in most EGFs. This is supported by the exceptions we found to the POGLUT2 and 3 consensus sequence.

Our analyses using *POGLUT2* and *POGLUT3* single-knockout cells provided insight into site-specific preferences between these two enzymes. We identified ten EGFs between FBN1, FBN2, and LTBP1 that are preferred by POGLUT2 and four preferred by POGLUT3. This suggests that POGLUT2 could have a more significant role in modifying FBN1, -2, and LTBP1, whereas POGLUT3 may be targeted toward other proteins yet to be identified as *O*-glucosylated. Thus, POGLUT2 and POGLUT3 may not be strictly redundant. Comparison of sequences between cysteines 3 and 4 modified by POGLUT2 or POGLUT3 did not reveal any significant differences, indicating that sequences outside of the consensus sequence may be influencing specificity (Fig. 8). This was further supported by our attempt to change site specificity of EGF12 of FBN1 and -2 (Fig. 9). Transcriptional analysis of POGLUT2 and POGLUT3 in embryonic stem cells and epiblast-like cells further highlights these enzymes are not completely redundant: POGLUT3 mRNA transcript levels were greatly increased in epiblast-like cells compared with POGLUT2 (43).

The POGLUT2 and 3 consensus sequence, C^3^-x-N-T-x-G-S-(FY)-x-C^4^, overlaps the consensus sequence for β-hydroxylation, C^3^-x-(ND)-x-x-x-x-(FY)-x-C^4^ (35). The majority of POGLUT2 and/or 3 modified EGFs on endogenous FBN1 were also β-hydroxylated. Interestingly, β-hydroxylation was reduced in recombinant FBN1 expressed in HEK293T cells compared with endogenous FBN1 secreted from human dermal fibroblasts. Previous studies show that β-hydroxylation is necessary for efficient calcium binding to EGF repeats (31, 32, 33). Calcium-binding affinities are highly variable across FBN1 (44). Binding of calcium provides rigidity to the structure of FBN1 and affords protection from proteolysis (44). A subset of MFS patient mutations in FBN1 affect residues involved in calcium binding, and these mutations have been linked to increased susceptibility to proteolysis (45, 46). FBN2 and LTBP1 have similar domain organization to FBN1 and are primarily composed of calcium-binding EGF repeats. It is possible that POGLUT2 and 3 *O*-glucosylation of EGF repeats is connected to β-hydroxylation and calcium binding of FBN1, FBN2, and LTBP1. Some of the defects in FBN1 structure or function related to MFS mutations could be due to loss of *O*-glucosylation rather than changes in β-hydroxylation. Further studies will need to be performed to investigate this.

Lastly, we have shown that secretion of FBN1-N is reduced in *POGLUT2*, *POGLUT3*, and *POGLUT2* and *3* knockout cells. This coincides with previous work demonstrating that *O*-glycosylation by POGLUT1 and POFUT1 stabilizes Notch EGF repeats and promotes cell-surface expression of the receptor (40). Like POGLUT1 and POFUT1, POGLUT2 and 3 have an endoplasmic reticulum (ER) retention sequence, localizing them to the ER where protein folding for the secretory pathway occurs (47). POGLUT2 and POGLUT2 also only modify folded EGF repeats, indicating that they can differentiate a folded from an unfolded structure (15). Combined with our secretion assay results, this indicates that *O*-glucosylation by POGLUT2 and 3 assists folding of substrate proteins and is necessary for efficient secretion. It is also possible that these *O*-glucose modifications are involved in the many binding interactions that occur in the ECM between FBN1, -2, and LTBP1 and other proteins required for proper development. *O*-glycans such as *O*-fucose modifications found on Notch receptor are directly involved in Notch-ligand binding, affecting Notch signaling (26, 27, 48, 49, 50). This provides a precedent for the involvement *O*-glycans on EGF repeats in protein–protein interactions. Microfibrils are a complex network of many protein–protein interactions that *O*-glucosylation by POGLUT2 and 3 may affect (1, 51). Further work is needed to understand the role of such *O*-glycans in the context of the ECM.

## Experimental procedures

### Plasmids and mutagenesis

pDNSP-rf16 (FBN1-N), pcDNA-rf6h (FBN1-C), pcDNA-rFBN2-2 (FBN2-N), pDNSP-rFBN2-1 (FBN2-C), and pCEP-Pu hLTBP1 FL-His have been described previously (30, 31, 32). The FBN1 and 2 plasmids were kindly gifted by Dr Dieter Reinhardt (McGill University), and the LTBP1 plasmid was kindly gifted by Dr Clair Baldock (The University of Manchester). All FBN1-N and FBN2-N mutants were generated using site-directed mutagenesis with CloneAmp HiFi Premix (Takara Bio). Primers used to generate each mutant are listed in Table S1. PCR products were digested with DpnI for 1 h at 37 °C and transformed into DH5α-competent cells (Invitrogen). All mutated plasmids were confirmed by sequencing. pKR5-IgG plasmid encoding human IgG was used as a transfection control for secretion assays as previously described (40, 41).

### Cell culture

All cells were maintained at 37 °C and 5% CO_2_ in DMEM high-glucose media supplemented with 10% bovine calf serum and 1% penicillin/streptomycin. HEK293T cells were obtained from ATCC. *POGLUT2* and *POGLUT3* single- and double-knockout HEK293T cells have been previously described (15). Primary human dermal fibroblasts derived from adult foreskin were purchased from ATCC.

### Antibodies

Rabbit-derived IgG against the proline-rich N-terminal region of human FBN1 (52) was generously provided by Dr Penny Handford (Oxford University).

### Immunoprecipitation of endogenous FBN1 from primary fibroblasts

Fibroblasts were cultured to confluency in a 10 cm dish without changing media. Approximately 7 days post confluency, culture media was collected and cleared. For immunoprecipitation, 50 μl of magnetic beads (Protein G Dynabeads, Invitrogen) was incubated with 4 μl anti-FBN1 antibody (diluted in 200 μl 1× PBS, 0.02% Tween) for 20 min rotating at room temperature. One wash was performed with 200 μl 1× PBS, 0.02% Tween. Beads were resuspended in 1 ml conditioned culture media and incubated for 1 h rotating at room temp. Three washes were performed with 1× PBS, 0.02% Tween. FBN1 was eluted from beads in 30 μl of 8 M urea in 400 mM ammonium bicarbonate at 37 °C for 15 min. Samples were stored at −20 °C.

### Protein expression and purification

For each replicate, five 10 cm dishes of approximately 90% confluent HEK293T cells were transiently transfected. This was done for each plasmid and cell condition (wild-type, single-knockout, and double-knockout cells). Prior to transfection, complete media was removed, cells were washed once with 1× PBS, and 6 ml Opti-MEM (Invitrogen) reduced serum media was added. Cells were transfected using PEI and 5 μg plasmid per 10 cm dish (DNA:PEI 1:6) in 500 μl OPTI-MEM. Media was harvested after approximately 3 days. Media was cleared by centrifugation followed by a 0.45 μm syringe filter. 5 M NaCl and 1 M imidazole were added to a final concentration of 500 mM and 10 mM, respectively. For purification, a 350 to 400 μl Ni-NTA bead volume (700–800 μl 50% slurry) was used (Qiagen). Wash buffer consisted of 500 mM NaCl and 10 mM imidazole in 1× TBS. Proteins were eluted using 250 mM imidazole in 1× TBS. Purified proteins were analyzed by SDS-gel electrophoresis (Fig. S1).

### Mass spectral analysis

Purified protein (15–20 μl) was transferred to Protein LoBind tubes. Tubes were placed in speed vacuum for 10 to 15 min to concentrate samples. Proteins were denatured and reduced using 10 μl of reducing buffer containing 8 M Urea, 400 mM ammonium bicarbonate, and 10 mM TCEP at 50 °C for 5 min. Alkylation was performed at room temperature in the dark with 100 mM iodoacetamide in 50 mM TrisHCl for 30 min to 1 h. Mass spectral grade water (45 μl) was added to each sample. Trypsin (cleaves C-terminal to lysine and arginine, Thermo Scientific Pierce 90057) and/or V8 (cleaves C-terminal glutamic and aspartic acid, Millipore Sigma P6181) or chymotrypsin (cleaves C-terminal to tyrosine, phenyalanine, tryptophan, leucine, or isoleucine, Sigma 90056) protease was added without exceeding 500 ng of total enzyme per sample. Samples were incubated in 37 °C water bath for 4 to 6 h. Formic acid (7 μl of 5%) was added and samples were sonicated for 20 min. Samples were desalted with Millipore C18 Zip Tip Pipette Tips. After elution in 50% acetonitrile, 0.1% acetic acid, samples were diluted to an approximate concentration of 10 ng/μl, 17% acetonitrile, and 0.1% formic acid. Approximately 10 ng of each sample was injected on a Q-Exactive Plus Orbitrap mass spectrometer (Thermo Fisher) with an Easy nano-LC HPLC system with a C18 EasySpray PepMap RSLC C18 column (50 μm × 15 cm, Thermo Fisher Scientific). A 30 min binary gradient solvent system (Solvent A: 0.1% formic acid in water and Solvent B: 90% acetonitrile, 0.1% formic acid in water) with a constant flow of 300 nl/min was used. Positive polarity mode was used with an m/z range of 350 to 2000 at a resolution of 35,000 and automatic gain control set to 1 × 10^6^. Higher-energy collisional dissociation–tandem mass spectrometry (HCD-MS/MS) was used on the top ten precursor ions in each full scan (collision energy set to 27%, 2 × 10^5^ gain control, isolation window m/z 3.0, dynamic exclusion enabled, and 17,500 fragment resolution). PMi-Byonic (v.2.10.5), and Proteome Discoverer (v2.1) was used to identify peptides. Fixed modifications: Carbamidomethyl +57.021464 at C. Variable modifications: Oxidation +15.994915 at M,H,N,D, Deamidated +0.984016 at N, and Ammonia-loss −17.026549 at N-Term C. Precursor mass tolerance was set to 20 ppm and fragment mass tolerance was set to 30 ppm. Two missed cleavages were allowed. Protein and peptide false discovery rates were set to a threshold of 1% and calculated in Byonic software version 2.10.5 (Protein Metrics) using the two-dimensional target decoy strategy as described (53). All data was searched against either a human FBN1 database (Uniprot accession number P35555 version 4 updated April 10, 2019, one entry), FBN2 (Uniprot accession number P35556 version 3 updated May 26, 2009, one entry), or LTBP1 (Uniprot accession number Q14766 version 4 updated March 2, 2010, one entry). Xcalibur Qual Browser (v2.0.3) was used to generate EICs for all identified peptides. For each peptide, area under the curve was calculated for each peak corresponding to searched glycoforms. Relative abundance was calculated by comparing area under the curve for a single glycoform to the total area under curve for all searched glycoforms of a specific peptide. Glycoforms searched: unmodified peptide, unmodified peptide plus β-hydroxylation, modified peptide with *O*-hexose, and modified peptide with *O*-hexose plus β-hydroxylation.

### Cell-based secretion assays

Wild-type, single-knockout, and double-knockout HEK293T cells were transiently cotransfected with the plasmid encoding human FBN1-N and human IgG as described (40, 41). Approximately 8 to 9 × 10^5^ cells in DMEM High-Glucose media supplemented with 10% BCS were seeded into six well plates approximately 24 h prior to transfection. Media was changed to 800 μl of Opti-MEM reduced serum media before adding transfection mix. Each transfection mix consisted of 1.5 μg FBN-N plasmid, 0.1 μg of IgG plasmid, 9 μl PEI, and 150 μl Opti-MEM. Cells were incubated for approximately 48 h after transfection. Media was harvested and cleared by centrifugation.

### Western blotting

Cleared conditioned medium (36 μl) was mixed with 12 μl 4× reducing sample buffer containing 2-mercaptoethanol. Samples were incubated on heat block at 100 °C for 10 min. Gradient gels (10-lane, 4–20%, Bio-Rad) were used to resolve proteins. Semidry transfer was performed using Invitrogen Power Blotter XL, nitrocellulose power blotter select transfer stacks, and 1× power blotter transfer buffer. Membranes were blocked at 4 °C overnight in 5% milk in 1× TBS 0.1% Tween 20. Membranes were incubated with mouse anti-His antibody (Bio-Rad catalog number MCA1396) at 1:1000 dilution in blocking solution for 1 h at room temp. Secondary antibodies, IRDye680RD goat (polyclonal) anti-mouse IgG (LI-COR catalog number 925-68070), and IRDye800CW goat (polyclonal) anti-human IgG (H + L) (LI-COR catalog number 925-32232), were incubated with membrane at 1:10,000 dilution for 1 h at room temperature. Blots were imaged and quantified using an LI-COR Odyssey CLx and Odyssey Imager software.

### Experimental design and statistical rationale

See Table S40.

## Data availability

The mass spectrometry proteomics data have been deposited to the ProteomeXchange Consortium *via* the PRIDE (54) partner repository with the data set identifier PXD025725.

## Supporting information

This article contains supporting information.

## Conflict of interest

The authors declare that they have no conflicts of interest with the contents of this article.
