# Supplementary material for: POGLUT2 and POGLUT3 O-glucosylate multiple EGF repeats in fibrillin-1, -2, and LTBP1 and promote secretion of fibrillin-1
Source: J Biol Chem. 2021 Aug 17;297(3):101055. doi: 10.1016/j.jbc.2021.101055 (PMC8405936; doi:10.1016/j.jbc.2021.101055)

Supporting Information for:

POGLUT2 and POGLUT3 *O*-glucosylate multiple EGF repeats in fibrillin-1, -2, and LTBP1 and promote secretion of FBN1

Daniel B. Williamson, Camron J. Sohn, Atsuko Ito and Robert S. Haltiwanger

Contents:

Supporting Information Tables S1-S40
Supporting Information Figures S1-S64

Supporting Information Tables:

Table S1. Primers used for site-directed mutagenesis of FBN1-N and FBN2-N.

Template	Mutation	Direction	Sequence	T_m
rhFBN1-N	F788Y V789S	FWD	5'-TGGAAGTTATAGCTGTACCTGCCCCAAGGGATTTATC-3'	74 C
		REV	5'-AGGTACAGCTATAACTTCCAGGAGTATTTCTACATTGTCCA-3'	73.7 C
rhFBN2-N	Y832F S833V	FWD	5'-AGGAAGTTTCGTCTGTACGTGCCCACCAGGGTAT-3'	74.5 C
		REV	5'-ACGTACAGACGAAACTTCCTGGCGTGTTTCGGCA-3'	74.5 C
rhFBN1-N	L744T	FWD	5'-GTGAAAACACTCGTGGGACCTATAAATGTATATGCAATTCAGGATATGAAG-3'	78 C
		REV	5'-GGTCCCACGAGTGTTTTCACAGATTCCATTTGGGCAAATATC-3'	77.3 C
rhFBN1-N	T747S	FWD	5'-CTTCGTGGGTCCTATAAATGTATATGCAATTCAGGATATGAAGTG-3'	79.8 C
		REV	5'-CATTTATAGGACCCACGAAGGTTTTCACAGATTCCATTTGG-3'	79.6 C
rhFBN1-N L744T	L744T + T747S	FWD	5'-ACTCGTGGGTCCTATAAATGTATATGCAATTCAGGATATGAAGTG-3'	79.8 C
		REV	5'-CATTTATAGGACCCACGAGTGTTTTCACAGATTCCATTTGG-3'	79.6 C

Table S2: FBN1, FBN2, and LTBP1 EGFs with variable residues at position 4 of the POGLUT2 and 3 consensus sequence. Sequence at the bottom is a WebLogo summary for EGFs in the table with low or no modification by POGLUT2 and 3**.**

Protein	EGF repeat	Sequence C^3-C^4	*O*-glucosylation Efficiency
**FBN-1**	7	CINNQGSYTC	High
	9	CINEDGSFKC	High
	13	CKNSPGSFIC	High
	29	CINMVGSFRC	unmodified
	34	CQNLDGSYRC	unmodified
**FBN-2**	9	CINEDGSFKC	High
	10	CINSEGSFRC	High
	13	CRNNLGSFNC	High
	14	CVNSKGSFHC	High
	19	CTNSEGSYEC	High
	22	CLNIPGSFKC	medium
**LTBP1**	7	CVNSPGSYQC	low
	8	CSNLEGSYMC	low
	14	CENVEGSFLC	unmodified
**Consensus Sequence:**	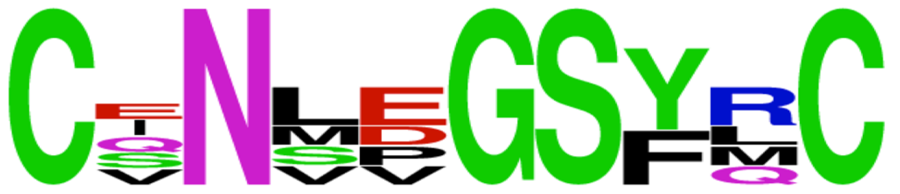		

Table S3. POGLUT2 versus POGLUT3 preferred modification sites on FBN1, FBN2, and LTBP1.

POGLUT2-preferred sites	Protein	EGF	Sequence (C^3-C^4)
	FBN1	4	CINTVGSFEC
		6	CIPTPGSYRC
		9	CINEDGSFKC
		12	CRNTPGSFVC
		21	CENTKGSFIC
	FBN2	9	CINEDGSFKC
		21	CENTKGSFIC
		22	CLNIPGSFKC
	LTBP1	5	CENTEGSFLC
		18	CINTDGSYKC
**POGLUT3-preferred sites**	FBN1	8	CINTDGSFHC
	FBN2	12	CRNTPGSYSC
		14	CVNSKGSFHC
	LTBP1	10	CRNTEGSFQC

Table S4: Human protein database search results of proteins containing the old versus new POGLUT2 and 3 consensus sequence (Expasy Scan Prosite).

Table S5: Trypsin digestion of endogenous human FBN1 from conditioned medium of adult dermal fibroblasts.

Table S6: Trypsin digestion of recombinant human FBN1-N expressed in WT HEK293T cells.

Table S7: Trypsin and V8 double digestion of recombinant human FBN1-N expressed in WT HEK293T cells.

Table S8: Chymotrypsin digestion of recombinant human FBN1-N expressed in WT HEK293T cells.

Table S9: Trypsin digestion of recombinant human FBN1-N expressed in *POGLUT2/3* double knockout HEK293T cells.

Table S10: Trypsin and V8 double digestion of recombinant human FBN1-N expressed in *POGLUT2/3* double knockout HEK293T cells.

Table S11: Trypsin digestion of recombinant human FBN1-C expressed in WT HEK293T cells.

Table S12: Trypsin digestion of recombinant human FBN1-C expressed in *POGLUT2/3* double knockout HEK293T cells.

Table S13: Trypsin digestion of recombinant human FBN2-N expressed in WT HEK293T cells.

Table S14: V8 digestion of recombinant human FBN2-N expressed in WT HEK293T cells.

Table S15: Trypsin digestion of recombinant human FBN2-N expressed in *POGLUT2/3* double knockout HEK293T cells.

Table S16: Trypsin digestion of recombinant human LTBP1 expressed in WT HEK293T cells.

Table S17: Trypsin and V8 double digestion of recombinant human LTBP1 expressed in WT HEK293T cells.

Table S18: Trypsin digestion of recombinant human LTBP1 expressed in *POGLUT2/3* double knockout HEK293T cells.

Table S19: Trypsin and V8 double digestion of recombinant human LTBP1 expressed in *POGLUT2/3* double knockout HEK293T cells.

Table S20: Trypsin digestion of recombinant human FBN2-C expressed in WT HEK293T cells.

Table S21: Trypsin digestion of recombinant human FBN2-C expressed in *POGLUT2/3* double knockout HEK293T cells.

Table S22: V8 digestion of recombinant human FBN1-N expressed in WT HEK293T cells (analyzing EGF11 only).

Table S23: V8 digestion of recombinant human FBN1-N L744T expressed in WT HEK293T cells (analyzing EGF11 only).

Table S24: V8 digestion of recombinant human FBN1-N T747S expressed in WT HEK293T cells (analyzing EGF11 only).

Table S25: V8 digestion of recombinant human FBN1-N L744T/T477S expressed in WT HEK293T cells (analyzing EGF11 only).

Table S26: Trypsin digestion of recombinant human FBN1-N expressed in *POGLUT2* knockout HEK293T cells.

Table S27: Trypsin digestion of recombinant human FBN1-N expressed in *POGLUT3* knockout HEK293T cells.

Table S28: Trypsin digestion of recombinant human FBN2-N expressed in *POGLUT2* knockout HEK293T cells.

Table S29: Trypsin digestion of recombinant human FBN2-N expressed in *POGLUT3* knockout HEK293T cells.

Table S30: Trypsin digestion of recombinant human LTBP1 expressed in *POGLUT2* knockout HEK293T cells.

Table S31: Trypsin and V8 double digestion of recombinant human LTBP1 expressed in *POGLUT2* knockout HEK293T cells.

Table S32: Trypsin digestion of recombinant human LTBP1 expressed in *POGLUT3* knockout HEK293T cells.

Table S33: Trypsin and V8 double digestion of recombinant human LTBP1 expressed in *POGLUT3* knockout HEK293T cells.

Table S34: Trypsin digestion of recombinant human FBN1-N EGF12 mutant expressed in WT HEK293T cells.

Table S35: Trypsin digestion of recombinant human FBN1-N EGF12 mutant expressed in *POGLUT2* knockout HEK293T cells.

Table S36: Trypsin digestion of recombinant human FBN1-N EGF12 mutant expressed in *POGLUT3* knockout HEK293T cells.

Table S37: Trypsin digestion of recombinant human FBN2-N EGF12 mutant expressed in WT HEK293T cells.

Table S38: Trypsin digestion of recombinant human FBN2-N EGF12 mutant expressed in *POGLUT2* knockout HEK293T cells.

Table S39: Trypsin digestion of recombinant human FBN2-N EGF12 mutant expressed in *POGLUT3* knockout HEK293T cells.

Table S40: Number of biological replicates for mass spectral analysis and secretion assays.

Supporting Information Figures:

Figure S1. Purification of recombinant FBN1-N, FBN1-C, FBN2-N, FBN2-C, and LTBP1 expressed in HEK293T cells. Purified proteins were separated on 4-20% gradient gel under reducing conditions followed by Gel Code Blue staining (Invitrogen). Approximate, predicted molecular weights of each protein: FBN1-N (165 kDa), FBN1-C (140 kDa), FBN2-N (186 kDa), FBN2-C (137 kDa), and LTBP1 (187 kDA). Band between 50 and 75 kDa is bovine serum albumin leftover from culture medium supplemented with bovine calf serum.

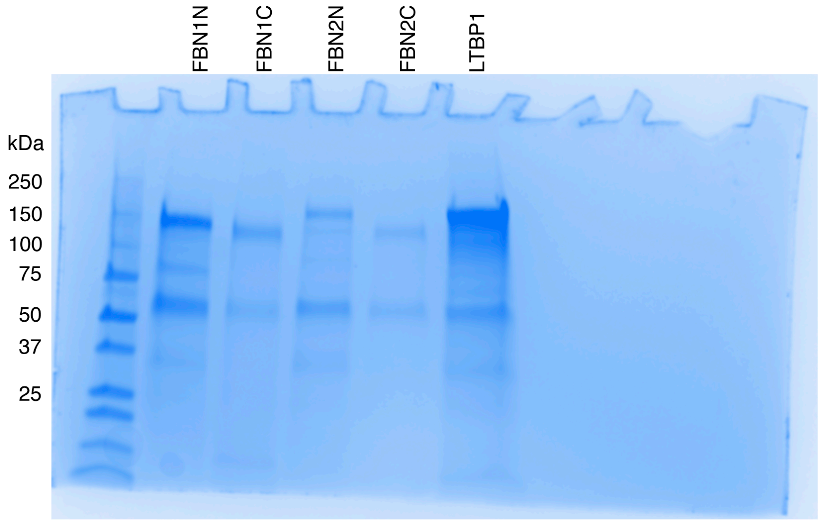

Figure S2. Clustal Omega protein sequence alignment of POGLUT2 versus POGLUT3 preferred EGF modification sites. EGFs with POGLUT3 preferred sites are underlined. Bold line above section of alignment indicates position of POGLUT2 and 3 consensus sequence. *****, single, fully conserved residue. **:** (colon), conserved amino acids of strongly similar properties. **.** (period), conserved amino acids of weakly similar properties. *A*, raw alignment. *B*, alignment with Clustal X default colors. *C*, alignment with Zappo colors. Jalview was used for color schemes.

A.

```
                       10        20        30        40
                   |    |    |    |    |    |    |    |
FBN1 EGF 4      DVDECQA---IPGLC-QGGNCINTVGSFECKCPAGHKLNEVSQKCE 42
FBN1 EGF 6      VTDYCQL---VRYLC-QNGRCIPTPGSYRCECNKGFQLDLRGE-CI 41
FBN1 EGF 9      DMDECS----IRNMC-LNGMCINEDGSFKCICKPGFQLASDGRYCK 41
FBN1 EGF 12     DINECVL---NSLLC-DNGQCRNTPGSFVCTCPKGFIYKPDLKTCE 42
FBN1 EGF 21     DVNECDL---NPNIC-LSGTCENTKGSFICHCDMGYSGKKGKTGCT 42
FBN2 EGF 9      DHDECT----TTNMC-LNGMCINEDGSFKCICKPGFVLAPNGRYCT 41
FBN2 EGF 21     DVNECDL---NSNIC-MFGECENTKGSFICHCQLGYSVKKGTTGCT 42
FBN2 EGF 22     DVDECEI---GAHNCDMHASCLNIPGSFKCSCREGWIGNGIK--CI 41
LTBP1 EGF 5     DIDECTQ---VQHLC-SQGRCENTEGSFLCICPAGFMASEEGTNCI 42
LTBP1 EGF 18    DVNECDELNNRMSLC-KNAKCINTDGSYKCLCLPGYVPSDKPNYCT 45
FBN1 EGF 8      DIDECLQ---NGRIC-NNGRCINTDGSFHCVCNAGFHVTRDGKNCE 42
FBN2 EGF 12     DIDECLV---NRLLC-DNGLCRNTPGSYSCTCPPGYVFRTETETCE 42
FBN2 EGF 14     DVNECEV---FPGVC-PNGRCVNSKGSFHCECPEGLTLDGTGRVCL 42
LTBP1 EGF10     DIDECQ----HRHLC-AHGQCRNTEGSFQCVCDQGYRASGLGDHCE 41
                  : *         *   . *    **: * *  *         *
```

B.

```
                10        20        30        40
FBN1  EGF 4     DVDECQA---IPGLC-QGGNCINTVGSFECKCPAGHKLNEVSQKCE 42
FBN1  EGF 6     VTDYCQL---VRYLC-QNGRCIPTPGSYRCECNKGFQLDLRGE-CI 41
FBN1  EGF 9     DMDECS----IRNMC-LNGMCINEDGSFKCICKPGFQLASDGRYCK 41
FBN1  EGF 12    DINECVL---NSLLC-DNGQCRNTPGSFVCTCPKGFIYKPDLKTCE 42
FBN1  EGF 21    DVNECDL---NPNIC-LSGTCENTKGSFICHCDMGYSGKKGKTGCT 42
FBN2  EGF 9     DHDECT----TTNMC-LNGMCINEDGSFKCICKPGFVLAPNGRYCT 41
FBN2  EGF 21    DVNECDL---NSNIC-MFGECENTKGSFICHCQLGYSVKKGTTGCT 42
FBN2  EGF 22    DVDECEI---GAHNCDMHASCLNIPGSFKCSCREGWIGNGIK--CI 41
LTBP1  EGF 5    DIDECTQ---VQHLC-SQGRCENTEGSFLCICPAGFMASEEGTNCI 42
LTBP1  EGF 18   DVNECDELNNRMSLC-KNAKCINTDGSYKCLCLPGYVPSDKPNYCT 45
FBN1  EGF 8     DIDECLQ---NGRIC-NNGRCINTDGSFHCVCNAGFHVTRDGKNCE 42
FBN2  EGF 12    DIDECLV---NRLLC-DNGLCRNTPGSYSCTCPPGYVFRTETETCE 42
FBN2  EGF 14    DVNECEV---FPGVC-PNGRCVNSKGSFHCECPEGLTLDGTGRVCL 42
LTBP1  EGF10    DIDECQ----HRHLC-AHGQCRNTEGSFQCVCDQGYRASGLGDHCE 41
                  : *         *   . *    **: * *  *         *
```

Clustal X Default Colouring

Category	Colour	Residue at position	{ Threshold, Residue group }
Hydrophobic	BLUE	A,I,L,M,F,W,V	{>60%, WLVIMAFCHP}
		C	{>60%, WLVIMAFCHP}
Positive charge	RED	K,R	{>60%,KR},{>80%, K,R,Q}
Negative charge	MAGENTA	E	{>60%,KR},{>50%,QE},{>85%,E,Q,D}
		D	{>60%,KR}, {>85%, K,R,Q}, {>50%,ED}
Polar	GREEN	N	{>50%, N}, {>85%, N,Y}
		Q	{>60%,KR},{>50%,QE},{>85%,Q,E,K,R}
		S,T	{>60%, WLVIMAFCHP}, {>50%, TS}, {>85%,S,T}
Cysteines	PINK	C	{>85%, C}
Glycines	ORANGE	G	{>0%, G}
Prolines	YELLOW	P	{>0%, P}
Aromatic	CYAN	H,Y	{>60%, WLVIMAFCHP}, {>85%, W,Y,A,C,P,Q,F,H,I,L,M,V}
Unconserved	WHITE	any / gap	If none of the above criteria are met

C.

```
                         10        20        30        40
FBN1  EGF  4     DVDECQA---IPGLC-QGGNCINTVGSFECKCPAGHKLNEVSQKCE  42
FBN1  EGF  6     VTDYCQL---VRYLC-QNGRCIPTPGSYRCECNKGFQLDLRGE-CI  41
FBN1  EGF  9     DMDECS----IRNMC-LNGMCINEDGSFKCICKPGFQLASDGRYCK  41
FBN1  EGF  12    DINECVL---NSLLC-DNGQCRNTPGSFVCTCPKGFIYKPDLKTCE  42
FBN1  EGF  21    DVNECDL---NPNIC-LSGTCENTKGSFICHCDMGYSGKKGKTGCT  42
FBN2  EGF  9     DHDECT----TTNMC-LNGMCINEDGSFKCICKPGFVLAPNGRYCT  41
FBN2  EGF  21    DVNECDL---NSNIC-MFGECENTKGSFICHCQLGYSVKKGTTGCT  42
FBN2  EGF  22    DVDECEI---GAHNCDMHASCLNIPGSFKCSCREGWIGNGIK--CI  41
LTBP1  EGF  5    DIDECTQ---VQHLC-SQGRCENTEGSFLCICPAGFMASEEGTNCI  42
LTBP1  EGF  18   DVNECDELNNRMSLC-KNAKCINTDGSYKCLCLPGYVPSDKPNYCT  45
FBN1  EGF  8     DIDECLQ---NGRIC-NNGRCINTDGSFHCVCNAGFHVTRDGKNCE  42
FBN2  EGF  12    DIDECLV---NRLLC-DNGLCRNTPGSYSCTCPPGYVFRTETETCE  42
FBN2  EGF  14    DVNECEV---FPGVC-PNGRCVNSKGSFHCECPEGLTLDGTGRVCL  42
LTBP1  EGF10     DIDECQ----HRHLC-AHGQCRNTEGSFQCVCDQGYRASGLGDHCE  41
                   : *         *   . *    **: * *  *         *
```

Aliphatic/hydrophobic	ILVAM
Aromatic	FWY
Positive	KRH
Negative	DE
Hydrophilic	STNQ
conformationally special	PG
Cysteine	C

Figures S3-S64: Annotated MS/MS spectra for all identified *O*-glucosylated peptides from FBN1, FBN2, and LTBP1. FBN1, FBN2, and LTBP1 were purified, digested, and analyzed by nano-LC-MS/MS as described in Experimental Procedures. Glycopeptides were identified using Byonic. Due to the lability of the fucose-peptide bond in HCD experiments, Byonic is occasionally unable to correctly assign the *O*-fucosylated Ser/Thr residue in a peptide. This is indicated in individual figures where the assignment is incorrect. MS/MS spectra for glycopeptides from FBN1 can be found in Figures S3-S29, for those from FBN2 in Figures S30-S56, and for those from LTPB1 in Figures S57-S64.

Figure S3
FBN1 EGF4

TGACQDVDECQAIPGLCQGGNCINTVG<u>S</u>FECK

● Hexose/Glucose

#	a obs.	b obs.	b-18 obs.	b++ obs.	Seq.	y obs.	y++ obs.	#
1					T			32
2					G			31
3		230.1138			A			30
4		390.1449			C			29
5		518.2044			Q			28
6		633.2289	615.2206		D			27
7	704.2938	732.2986	714.291		V			26
8	819.3124	847.3246	829.3102		D			25
9	948.3623	976.3675	958.3537	488.6883	E			24
10		1136.3804			C			23
11		1264.4512	1246.4553		Q			22
12		1335.4932	1317.476	668.251	A			21
13	1420.582	1448.5784	1430.585		I			20
14					P		1130.4869	19
15					G			18
16					L			17
17					C			16
18					Q	1832.7833	916.8813	15
19					G			14
20					G			13
21					N			12
22					C			11
23					I			10
24					N	1203.5056		9
25					T	1089.4867		8
26					V	988.431		7
27					G	889.3593		6
28					S-Hex(1)	832.3469		5
29					F	583.2547		4
30					E	436.1836		3
31					C	307.1434		2
32					K	147.1126		1

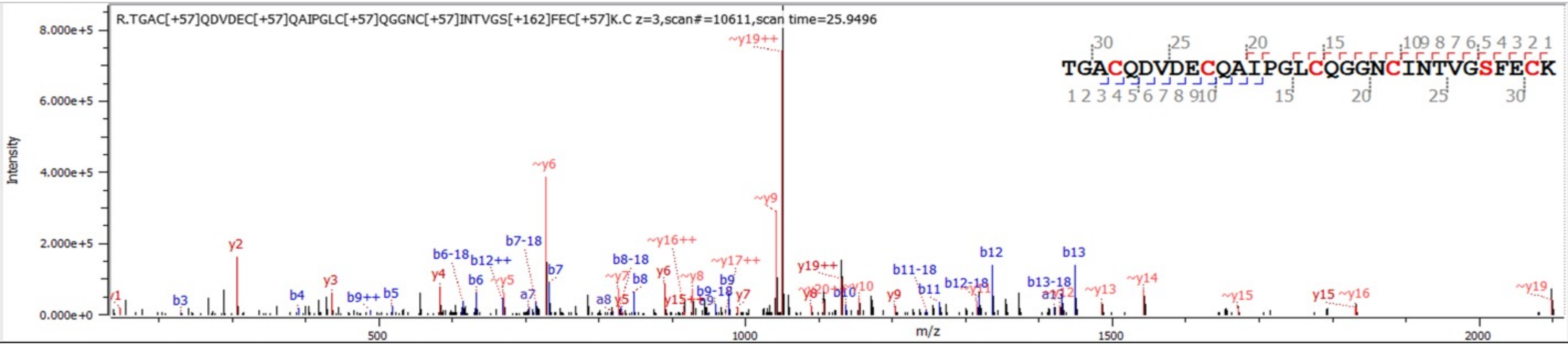

Figure S4
FBN1 EGF6

CIPTGSYR

Hexose/Glucose

#	a obs.	b obs.	Seq.	y obs.	y++ obs.	#
1		161.0381	C			9
2	246.1271	274.122	I			8
3		371.1736	P	939.4423	470.2251	7
4			T	842.3911	421.6971	6
5			P	741.3418		5
6			G	644.288		4
7			S-Hex(1)			3
8			Y	338.1793		2
9			R	175.1191		1

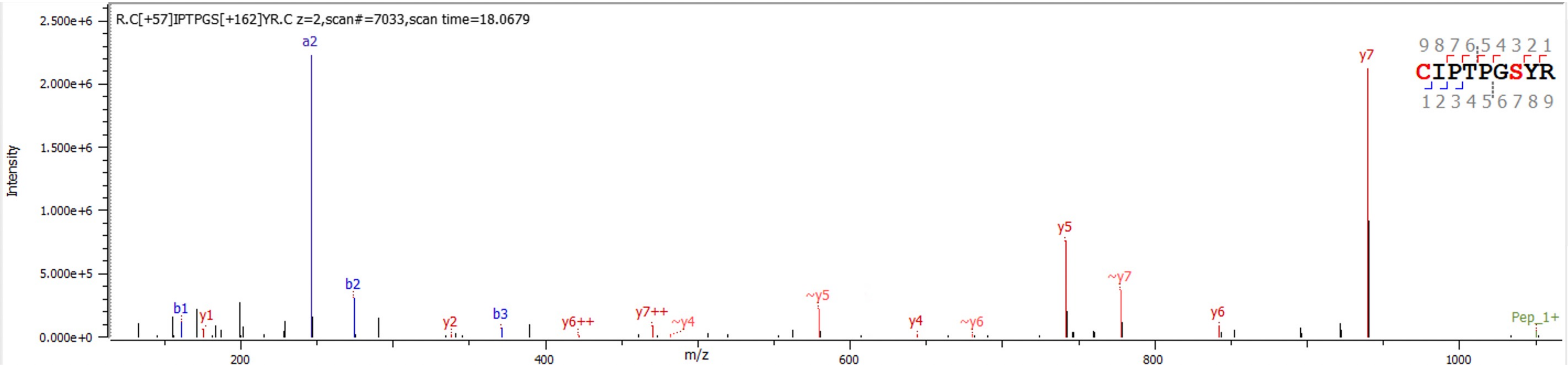

Figure S5
FBN1 EGF7

GECIDVDECEKNPCAGGECINNQGSYTCQCR

● Hexose/Glucose

#	a obs.	b obs.	b-18 obs.	b++ obs.	Seq.	y obs.	y++ obs.	#
1					G			31
2	159.0763	187.0716			E			30
3	319.1064	347.102	329.0901		C			29
4	432.1913	460.1786	442.1789		I			28
5	547.2209	575.2134	557.2032		D		1700.1442	27
6	646.2836	674.2816	656.267		V		1642.6339	26
7	761.3038	789.3123	771.2977		D		1593.12	25
8	890.3445	918.353	900.3378		E			24
9		1078.3826	1060.3942		C			23
10		1207.4247	1189.4352		E		1391.0447	22
11		1335.5182	1317.5073		K		1326.5123	21
12		1449.562	1431.5532		N			20
13		1546.5992		773.8121	P		1205.4688	19
14		1706.6584		853.8266	C			18
15	1749.6816	1777.6829		889.3486	A			17
16	1806.7063	1834.6909			G			16
17	1863.7161			946.3757	G			15
18	1992.7618	2020.7406		1010.8825	E			14
19				1090.9131	C	1838.6962		13
20				1147.4358	I	1678.6547	839.834	12
21					N-Oxidation	1565.5952		11
22					N	1435.5604		10
23				1333.5189	Q	1321.5159		9
24					G	1193.4596		8
25					S-Hex(1)	1136.4478		7
26					Y	887.3496		6
27					T	724.2864		5
28					C	623.2389		4
29					Q	463.2075		3
30					C	335.1497		2
31					R	175.1192		1

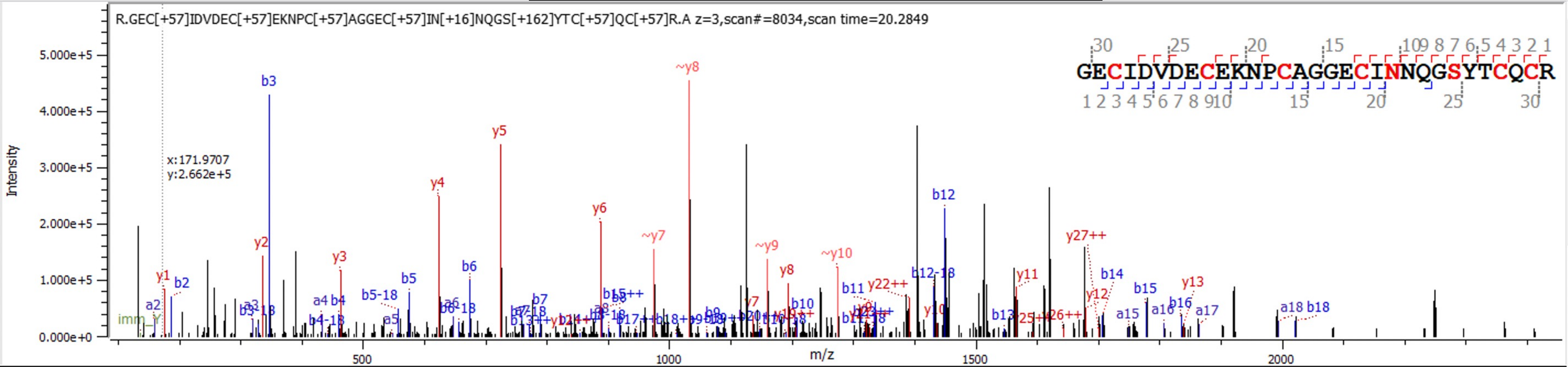

Figure S6
FBN1 EGF8

CINTDG<u>S</u>FHCVCNAGFHVTR (S marked with a blue circle)

● Hexose/Glucose

#	a obs.	b obs.	b-18 obs.	Seq.	y obs.	y++ obs.	#
1		161.038		C			20
2	246.1271	274.1225		I			19
3		404.1582		N-Oxidation			18
4				T			17
5		620.2342	602.2212	D			16
6			659.2599	G			15
7				S-Hex(1)			14
8				F		802.866	13
9				H	1457.6586	729.3309	12
10				C	1320.5939	660.8002	11
11				V	1160.5651	580.787	10
12				C	1061.4952	531.2509	9
13				N	901.4637	451.2338	8
14				A	787.4218	394.2144	7
15				G	716.3839	358.6954	6
16				F	659.3638		5
17				H	512.2944		4
18				V	375.2348		3
19				T	276.1666		2
20				R	175.1189		1

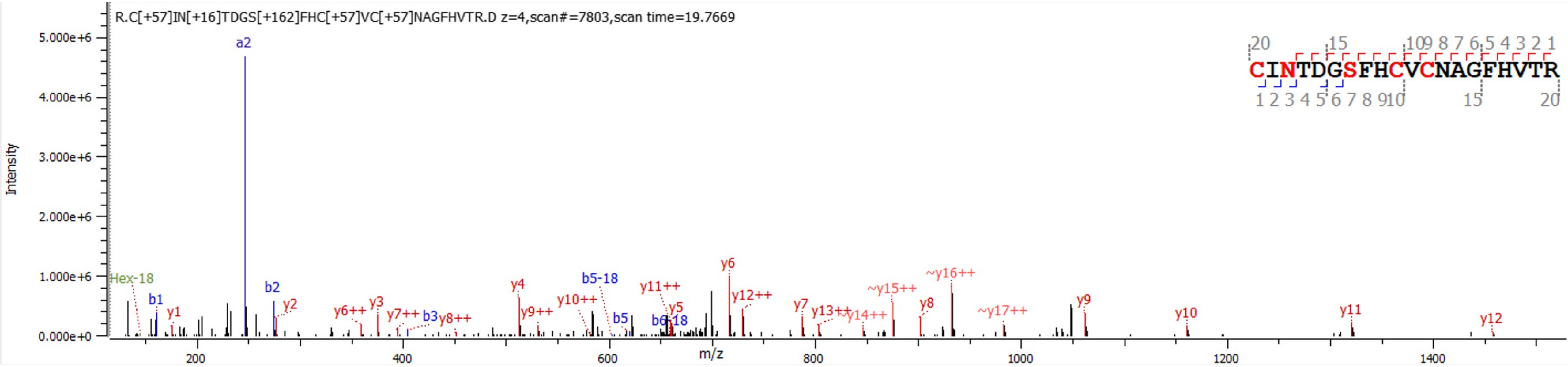

Figure S7
FBN1 EGF9

NMCLNGMCINEDGSFK

● Hexose/Glucose

#	a obs.	b obs.	Seq.	y obs.	#
1			N		16
2	218.0962	246.0912	M		15
3		406.1222	C		14
4		519.2045	L		13
5		649.2452	N		12
6			G	1419.5895	11
7			M		10
8			C		9
9			I		8
10			N	958.3941	7
11			E	844.3426	6
12			D	715.3066	5
13			G	600.2903	4
14			S-Hex(1)		3
15			F	294.1819	2
16			K	147.1127	1

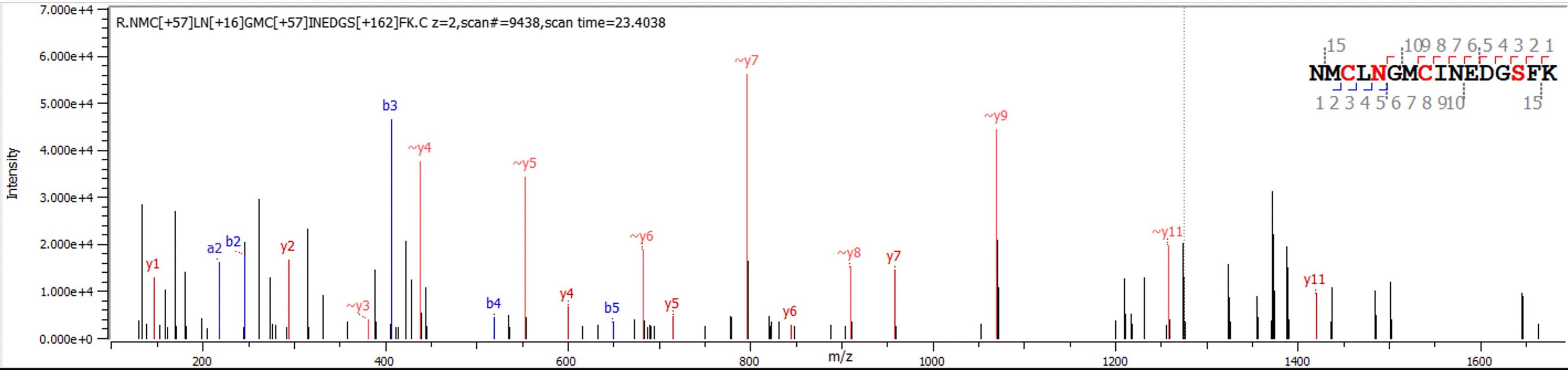

Figure S8
FBN1 EGF10

CVNTDGSYR

Hexose/Glucose

#	a obs.	b obs.	Seq.	y obs.	#
1		161.0381	C		9
2	232.1116	260.1063	V		8
3		374.1476	N	974.4066	7
4			T	860.3662	6
5			D	759.3134	5
6			G	644.2888	4
7			S-Hex(1)		3
8			Y	338.1828	2
9			R	175.1193	1

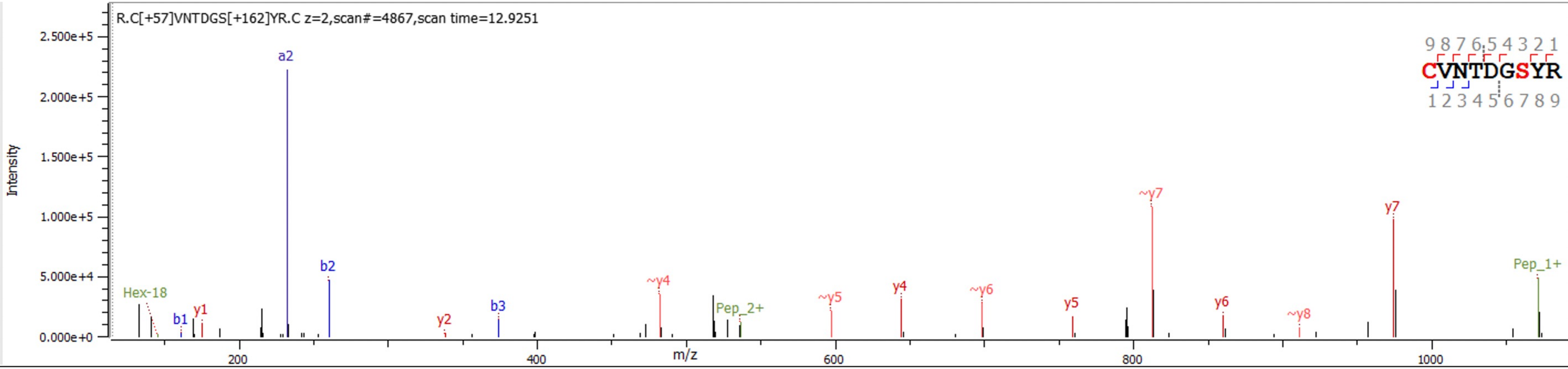

Figure S9
FBN1 EGF12

NTPGSFVCTCPK (S underlined, marked with a blue circle)

● Hexose/Glucose

#	a obs.	b obs.	b-18 obs.	Seq.	y obs.	y++ obs.	#
1				N-Oxidation			12
2	204.0981	232.0929	214.0818	T			11
3		329.1438	311.1344	P	1314.574		10
4				G	1217.5176		9
5				S-Hex(1)	1160.4976		8
6				F	911.4102		7
7		881.3702		V		382.6735	6
8				C	665.2748		5
9				T	505.2437		4
10				C	404.1969		3
11				P	244.1658		2
12				K	147.1131		1

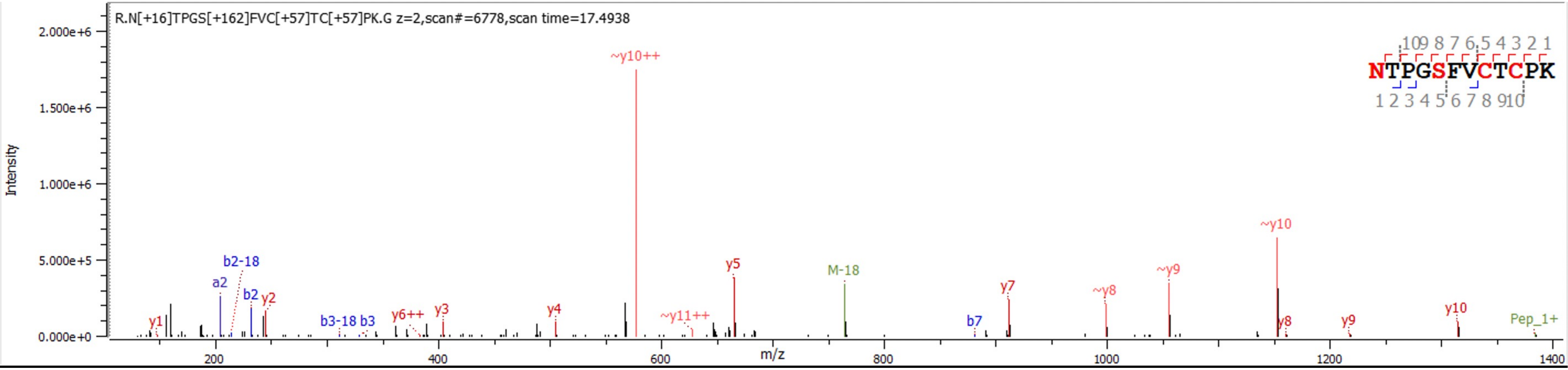

Figure S10
FBN1 EGF13

NSPGSFICECSSESTLDPTK

● Hexose/Glucose

*Hexose is assigned to wrong residue. This is not uncommon to find with certain peptides. Double knockout data confirmed this modification is being added by POGLUT2 and 3.

#	a obs.	b obs.	b-18 obs.	b++ obs.	Seq.	y obs.	#
1					N		20
2	174.0874	202.0823			S		19
3		299.1353	281.1244		P		18
4		356.1569	338.1472		G		17
5		443.1853	425.1789		S		16
6		590.2559	572.2442		F		15
7		703.3419	685.3306		I		14
8		863.373	845.3616	432.192	C		13
9		992.4092	974.4	496.7118	E		12
10		1152.4377			C		11
11					S-Hex(1)		10
12					S	977.4796	9
13					E	890.447	8
14					S	761.4039	7
15					T	674.3711	6
16					L	573.3253	5
17					D	460.2403	4
18					P	345.2134	3
19					T	248.1603	2
20					K	147.113	1

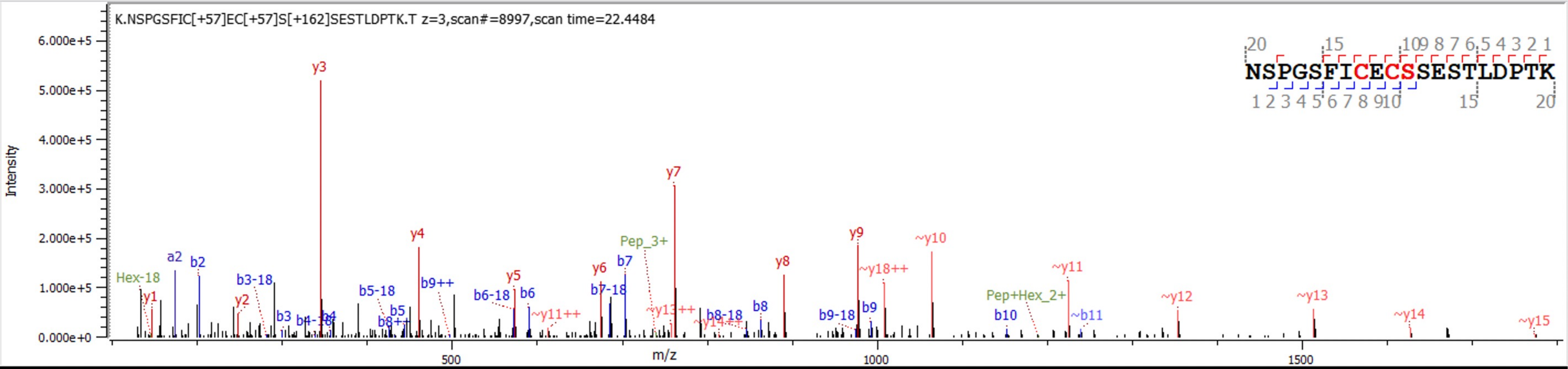

Figure S11
FBN1 EGF15

NTIGSFK

Hexose/Glucose

#	a obs.	b obs.	b-18 obs.	Seq.	y obs.	#
1				N		7
2	204.0984	232.0927	214.0822	T		6
3				I	713.375	5
4				G	600.2872	4
5				S-Hex(1)		3
6				F	294.1812	2
7				K	147.1128	1

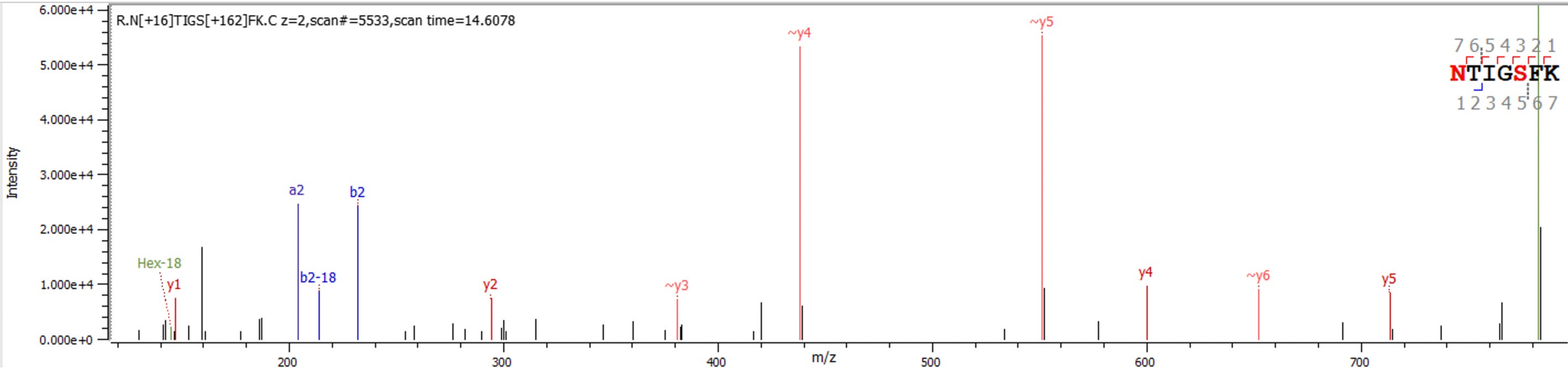

Figure S12
FBN1 EGF17

GGVCHNTEGSYR

● Hexose/Glucose

#	a obs.	b obs.	b-18 obs.	b++ obs.	Seq.	y obs.	#
1					G		12
2					G		11
3	186.1235	214.1187			V		10
4		374.151			C		9
5		511.2091			H		8
6		625.2543			N	988.4259	7
7		726.301	708.2871		T		6
8		855.3386	837.331	428.1793	E	773.3369	5
9					G	644.2907	4
10					S-Hex(1)	587.2703	3
11					Y	338.1821	2
12					R	175.1193	1

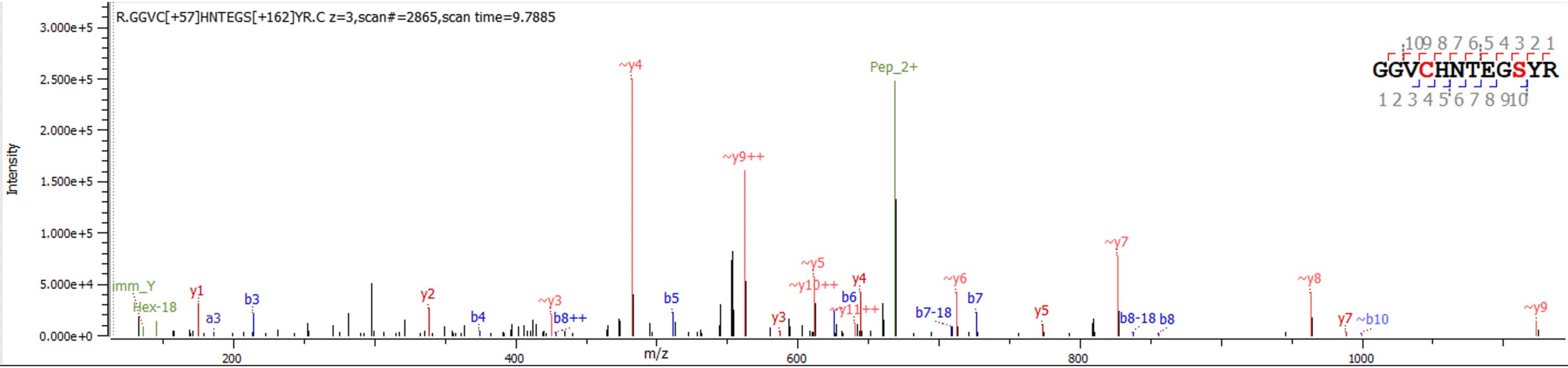

Figure S13
FBN1 EGF19

CTNSEGSYECSCQPGF

● Hexose/Glucose

*Hexose is assigned to wrong residue. This is not uncommon to find with certain peptides. Double knockout data confirmed this modification is being added by POGLUT2 and 3.

#	a obs.	b obs.	b-18 obs.	Seq.	y obs.	#
1		161.0382		C		16
2	234.0899	262.085	244.0741	T		15
3	348.1332	376.128	358.1173	N		14
4		625.2036		S-Hex(1)		13
5	726.2432	754.2474	736.2548	E		12
6				G	1291.4395	11
7		898.2851	880.2838	S		10
8		1061.3441		Y	1147.3947	9
9		1190.3944		E	984.3546	8
10				C	855.3119	7
11		1437.4808	1419.4343	S	695.2855	6
12				C	608.2488	5
13				Q	448.2186	4
14				P	320.16	3
15				G	223.1074	2
16				F	166.0861	1

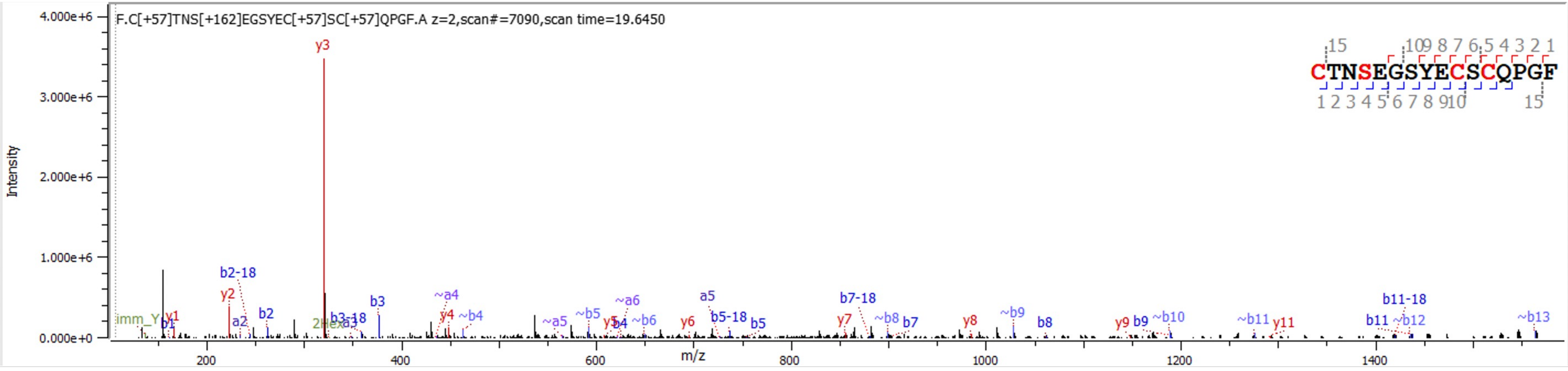

Figure S14
FBN1 EGF21

GSFICHCDMGYSGK

Hexose/Glucose

*Hexose is assigned to wrong residue. This is not uncommon to find with certain peptides. Double knockout data confirmed this modification is being added by POGLUT2 and 3.

#	a obs.	b obs.	b-18 obs.	Seq.	y obs.	#
1				G		14
2		145.0607		S		13
3		292.1289	274.119	F		12
4	377.2208	405.2128		I		11
5			547.2253	C		10
6	674.3032	702.3038		H		9
7				C		8
8		977.3536		D	919.3499	7
9				M		6
10		1165.4137		G		5
11		1328.49		Y		4
12				S-Hex(1)		3
13				G	204.1345	2
14				K	147.1128	1

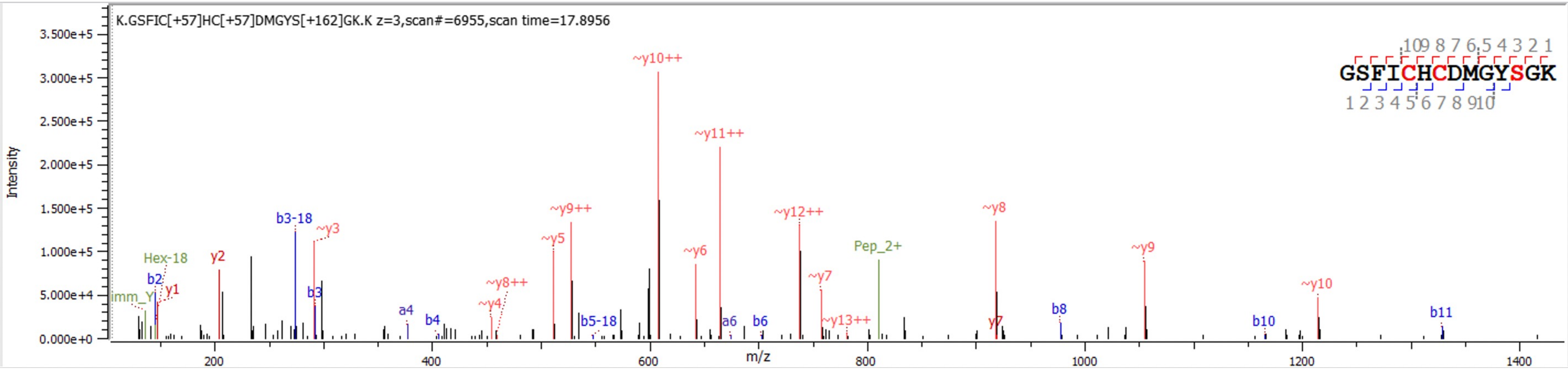

Figure S15
FBN1 EGF22

HAVCTNTAGSFK

● Hexose/Glucose

#	a obs.	b obs.	b-18 obs.	Seq.	y obs.	#
1		138.0664		H		12
2	181.1086	209.1035		A	1333.5991	11
3		308.1722		V	1262.5552	10
4	440.2109	468.2002		C	1163.4855	9
5		569.2487		T		8
6		699.2954	681.2723	N-Oxidation		7
7		800.3387	782.3177	T		6
8	843.3805	871.3713	853.3647	A		5
9		928.4021		G	600.2918	4
10				S-Hex(1)		3
11				F	294.1819	2
12				K	147.1131	1

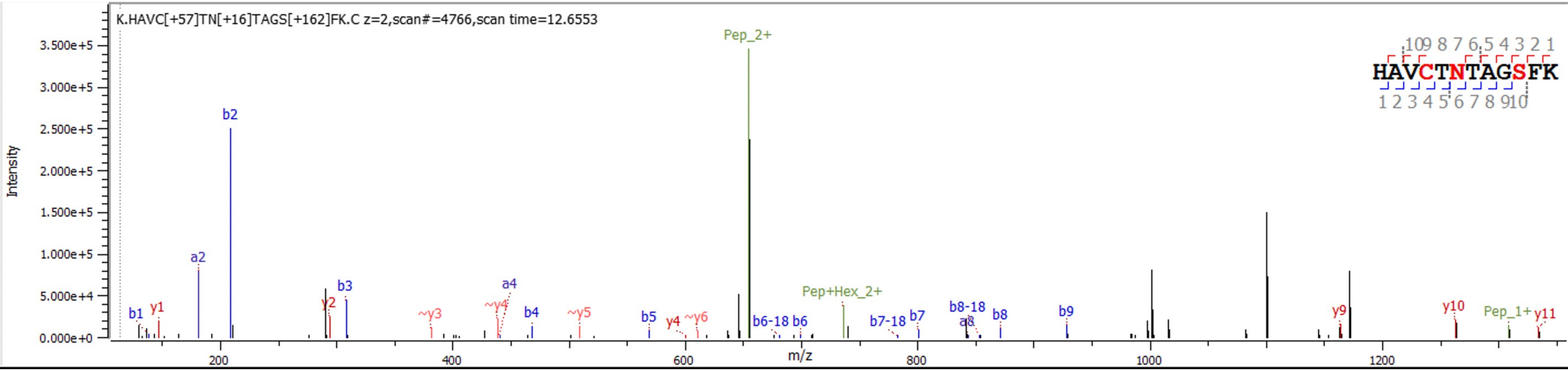

Figure S16
FBN1 EGF23

NTMGSYR

Hexose/Glucose

#	a obs.	b obs.	b-18 obs.	Seq.	y obs.	#
1				N		7
2	188.1032	216.0981	198.0872	T		6
3		347.139	329.1287	M	775.3292	5
4				G	644.2894	4
5				S-Hex(1)		3
6				Y	338.1819	2
7				R	175.1186	1

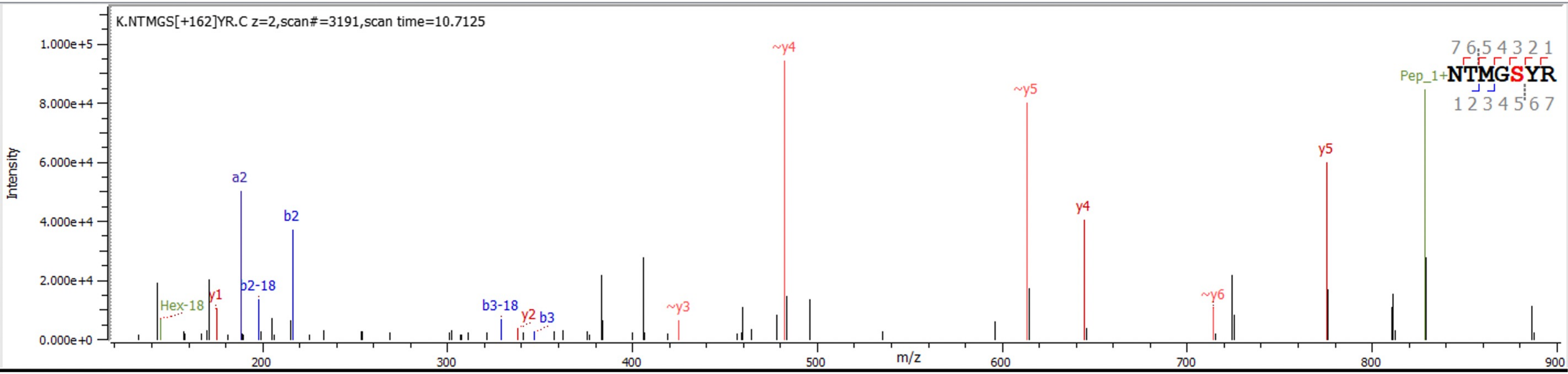

Figure S17
FBN1 EGF26

CLDPTTCISGNCVNTPG<u>S</u>YICDCPPDFE

● Hexose/Glucose

#	a obs.	b obs.	b-18 obs.	b++ obs.	Seq.	y obs.	#
1					C		28
2	246.1272	274.1221			L		27
3	361.1566	389.1483	371.1382		D		26
4	458.2155	486.2019			P		25
5		587.2512	569.2384		T		24
6	660.2981	688.2994	670.2855		T		23
7	820.3457	848.3301	830.3232		C		22
8	933.3953	961.4116	943.4029		I		21
9		1048.4434	1030.4233		S		20
10		1105.4662			G		19
11		1219.5133	1201.4862		N		18
12		1379.5409		690.2674	C		17
13		1478.6044	1460.5822		V		16
14		1592.632			N		15
15		1693.6943	1675.6794	847.3542	T		14
16					P		13
17					G		12
18					S-Hex(1)		11
19					Y		10
20					I	1152.4341	9
21					C	1039.3484	8
22					D	879.3344	7
23					C	764.2928	6
24					P	604.2617	5
25					P	507.2087	4
26					D	410.1608	3
27					F	295.129	2
28					E	148.0606	1

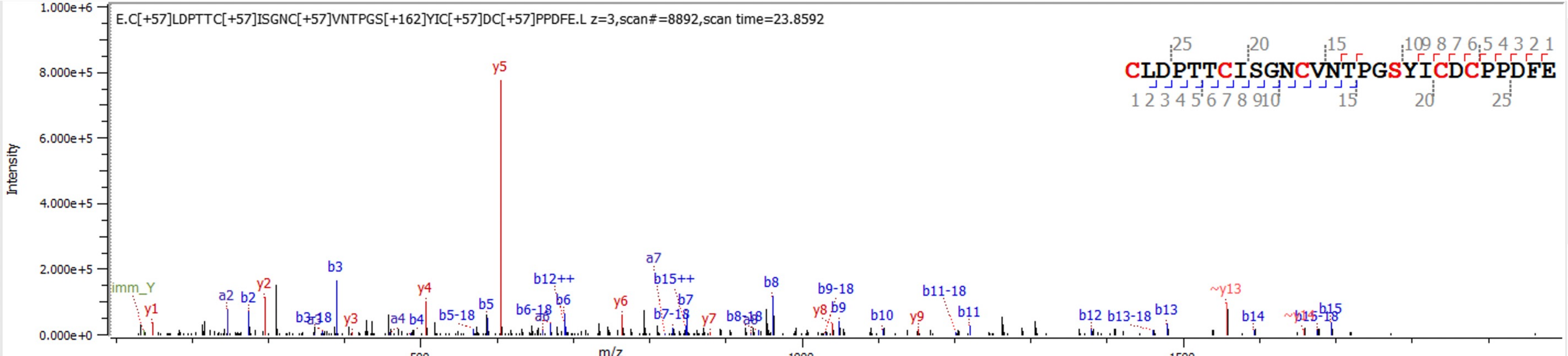

Figure S18
FBN1 EGF27

CINTFG<u>S</u>FQCR

● Hexose/Glucose

#	a obs.	b obs.	b-18 obs.	Seq.	y obs.	#
1		161.0382		C		11
2	246.1271	274.122		I		10
3	376.1674	404.1594		N-Oxidation	1294.5385	9
4	477.2105	505.2085	487.1966	T	1164.5013	8
5	624.2798	652.2755	634.2654	F	1063.4519	7
6				G	916.3832	6
7				S-Hex(1)	859.3575	5
8			1087.4628	F	610.2778	4
9				Q	463.2085	3
10				C	335.1499	2
11				R	175.1189	1

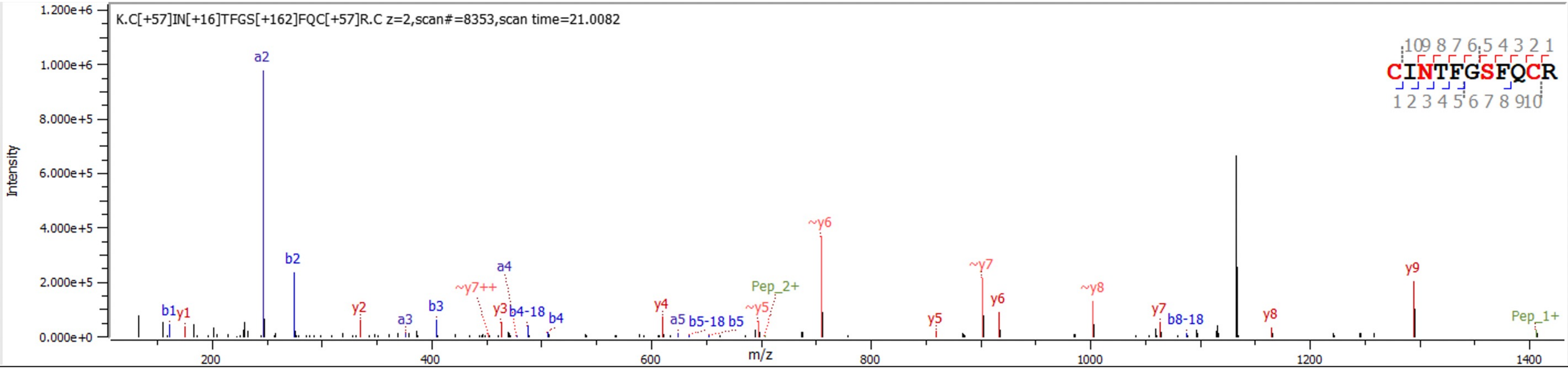

Figure S19
FBN1 EGF30

NAECINTAGSYR

● Hexose/Glucose

#	a obs.	b obs.	b-18 obs.	Seq.	y obs.	#
1				N		12
2	158.0925	186.0874		A		11
3		315.1301	297.1197	E	1348.5699	10
4	447.1663	475.161	457.1495	C	1219.5269	9
5	560.2501	588.2448	570.2346	I	1059.4967	8
6			700.2736	N-Oxidation	946.4116	7
7				T	816.375	6
8				A	715.3274	5
9		947.4143	929.3979	G	644.2874	4
10	1168.5016			S-Hex(1)	587.268	3
11				Y	338.1822	2
12				R	175.1191	1

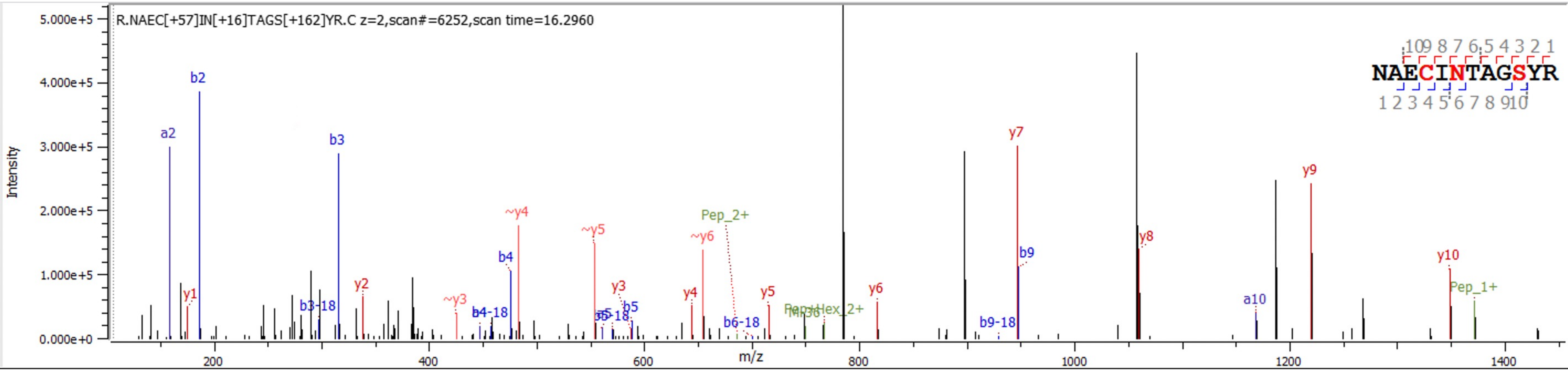

Figure S20
FBN1 EGF31

FTSTGQCNDRNECQEIPNICSHGQCIDTVG<u>S</u>FYCLCHTGFK

● Hexose/Glucose

#	a obs.	b obs.	b-18 obs.	b++ obs.	Seq.	y obs.	y++ obs.	y_3+ obs.	y_4+ obs.	#
1					F					41
2	221.1286	249.1234	231.1129		T					40
3		336.1552	318.1451		S					39
4	409.2105	437.2027	419.1917		T					38
5	466.2289	494.2249	476.2132		G					37
6		622.2821	604.2806		Q					36
7			764.2825		C					35
8		896.3544	878.3636		N				1074.7067	34
9					D					33
10		1167.493		584.2543	R					32
11		1281.5291		641.2719	N					31
12	1382.5795	1410.5721	1392.5342	705.7937	E					30
13				785.8086	C					29
14				849.8325	Q			1169.4962		28
15				914.3557	E					27
16		1940.8064		970.901	I					26
17					P		1568.678	1046.1133		25
18					N					24
19					I					23
20					C					22
21					S					21
22					H					20
23					G					19
24					Q					18
25					C					17
26					I					16
27					D-Oxidation					15
28					T					14
29					V					13
30					G		819.8435			12
31					S-Hex(1)					11
32					F	1332.5848	666.7975			10
33					Y	1185.5176	593.2633			9
34					C	1022.4551	511.731			8
35					L	862.4252	431.7145			7
36					C	749.3397	375.1716			6
37					H	589.3085				5
38					T	452.251				4
39					G	351.2025				3
40					F	294.1806				2
41					K	147.113				1

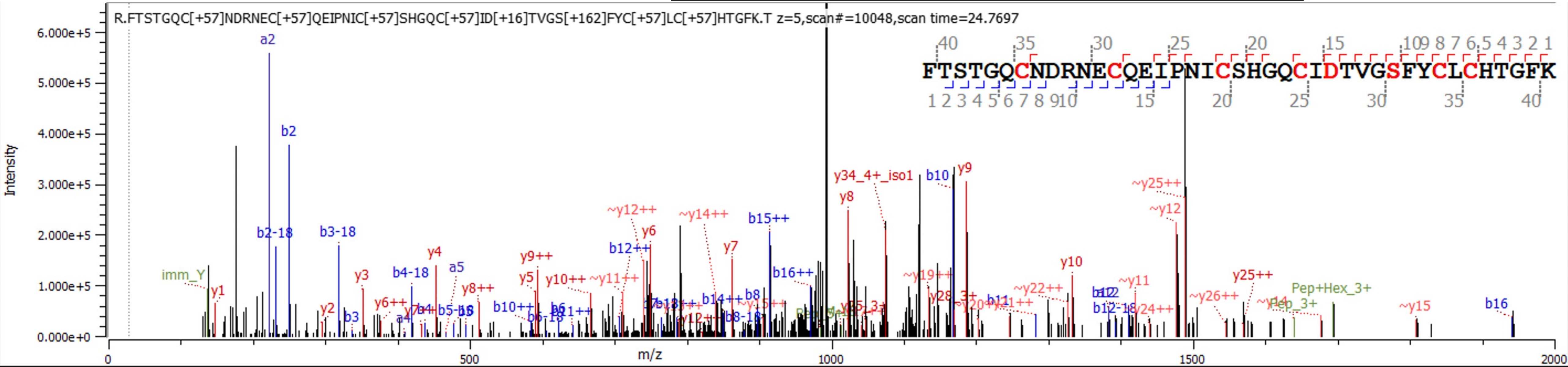

Figure S21
FBN1 EGF32

NTIG<u>S</u>FNCR

● Hexose/Glucose

#	a obs.	b obs.	b-18 obs.	Seq.	y obs.	#
1				N-Oxidation		9
2	204.098	232.0929	214.0823	T		8
3	317.1819	345.1769	327.1671	I	1015.458	7
4		402.1989	384.1878	G	902.3679	6
5	623.3046			S-Hex(1)	845.3478	5
6				F	596.2612	4
7				N	449.1933	3
8				C	335.1495	2
9				R	175.1191	1

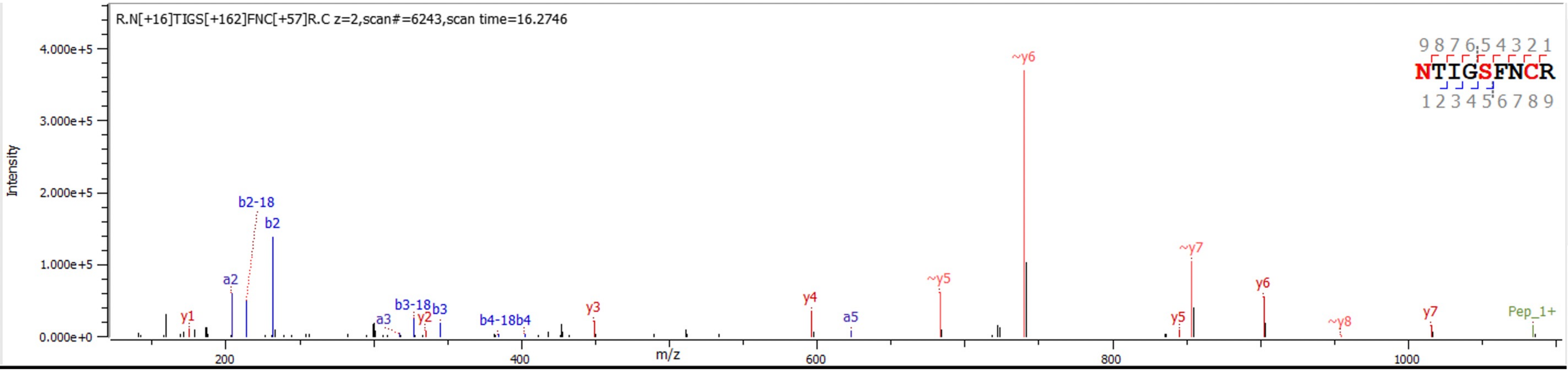

Figure S22
FBN1 EGF33

NGQCINTVGSFQCQCNEGYEVAPDGR

● Hexose/Glucose

#	a obs.	b obs.	b-18 obs.	b++ obs.	Seq.	y obs.	y++ obs.	#
1					N			26
2		172.0719			G			25
3		300.1302	282.1199		Q			24
4	432.1635	460.1606	442.1532		C			23
5	545.2509	573.2459	555.2341		I			22
6	675.2877	703.2842	685.2652		N-Oxidation			21
7		804.3278	786.3285		T			20
8	875.4053	903.4005	885.3919	452.2055	V			19
9		960.4227	942.406		G			18
10					S-Hex(1)			17
11					F			16
12					Q	1782.7035	891.8669	15
13					C	1654.6633	827.8344	14
14					Q	1494.6249	747.8156	13
15					C	1366.5692	683.7849	12
16					N	1206.5406		11
17					E	1092.4962		10
18					G	963.4539	482.2358	9
19					Y	906.4349		8
20					E	743.3689		7
21					V	614.3263		6
22					A	515.2578		5
23					P	444.2202		4
24					D	347.1671		3
25					G	232.1405		2
26					R	175.1194		1

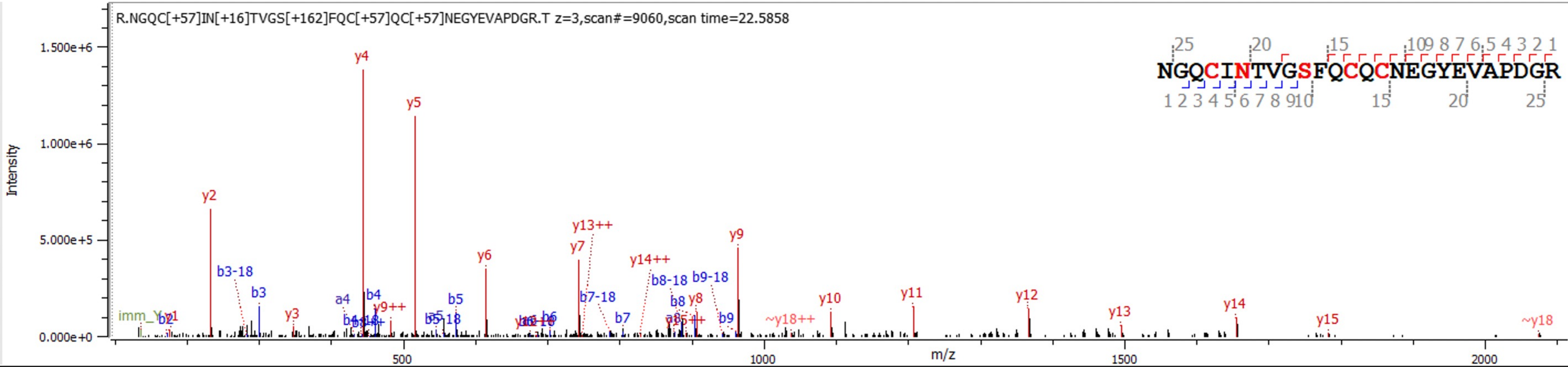

Figure S23
FBN1 EGF35

CICPPGYSLQNEKCEDIDECVEEPEICALGTCSNTEG<u>S</u>FK (hexose marker on S)

● Hexose/Glucose

#	a obs.	b obs.	b-18 obs.	b++ obs.	Seq.	y obs.	y++ obs.	#
1		161.0381			C			40
2	246.1272	274.1219			I			39
3	406.1599	434.1526			C			38
4		531.2056			P			37
5		628.2656			P			36
6					G			35
7	820.3527	848.3441			Y			34
8	1053.4471				S			33
9			1176.5082		L			32
10					Q			31
11					N			30
12			1547.6501		E			29
13					K			28
14					C			27
15					E		1584.1616	26
16				1049.4559	D			25
17					I			24
18					D		1405.5736	23
19					E			22
20					C			21
21					V			20
22					E			19
23					E		1089.4468	18
24					P		1024.9395	17
25					E		976.4006	16
26					I			15
27					C	1709.7139		14
28					A	1549.6501		13
29					L			12
30					G	1365.5428		11
31					T	1308.5238		10
32					C	1207.4921		9
33					S	1047.4348		8
34					N-Oxidation	960.4089		7
35					T	830.3636		6
36					E			5
37					G	600.29		4
38					S-Hex(1)	543.2687		3
39					F	294.181		2
40					K	147.113		1

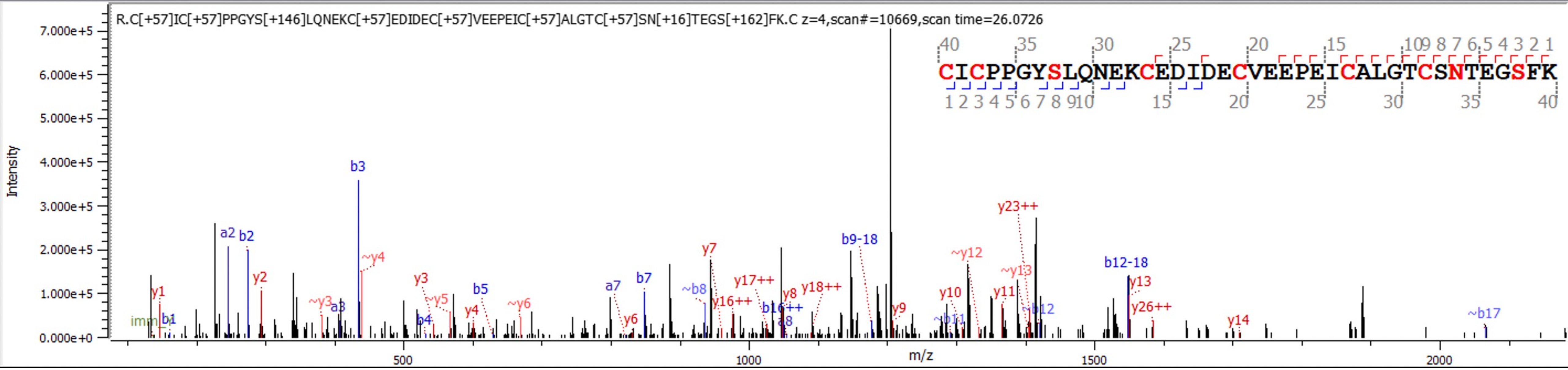

Figure S24
FBN1 EGF36

HGQCINTDGSYR (S underlined, marked with ●)

● Hexose/Glucose

#	a obs.	b obs.	b-18 obs.	Seq.	y obs.	#
1				H		12
2	167.0933	195.0879		G		11
3		323.146	305.1362	Q		10
4	455.1839	483.1772	465.1612	C		9
5	568.265	596.2607		I		8
6	698.3096	726.2966	708.2867	N-Oxidation	990.3987	7
7				T	860.3597	6
8		942.3856		D	759.3157	5
9				G	644.2892	4
10				S-Hex(1)		3
11				Y	338.182	2
12				R	175.1192	1

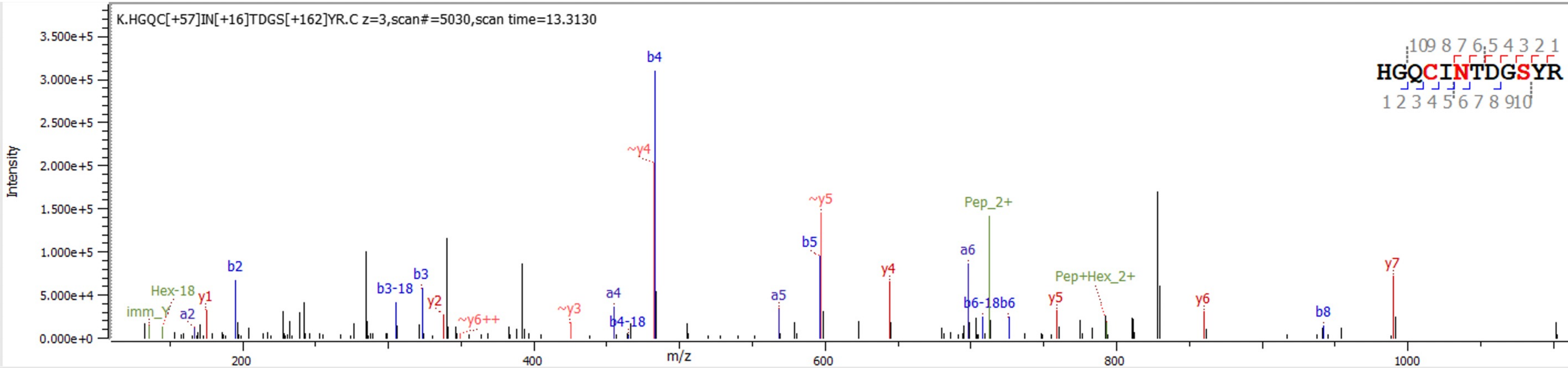

Figure S25
FBN1 EGF38

CVNTYGSYECK

Hexose/Glucose

#	a obs.	b obs.	b-18 obs.	Seq.	y obs.	#
1		161.0373		C		11
2	232.1117	260.1062		V		10
3		390.1445		N	1299.506	9
4			473.1824	T		8
5				Y		7
6				G	905.3625	6
7				S-Hex(1)		5
8				Y	599.2511	4
9				E	436.185	3
10				C	307.1438	2
11				K		1

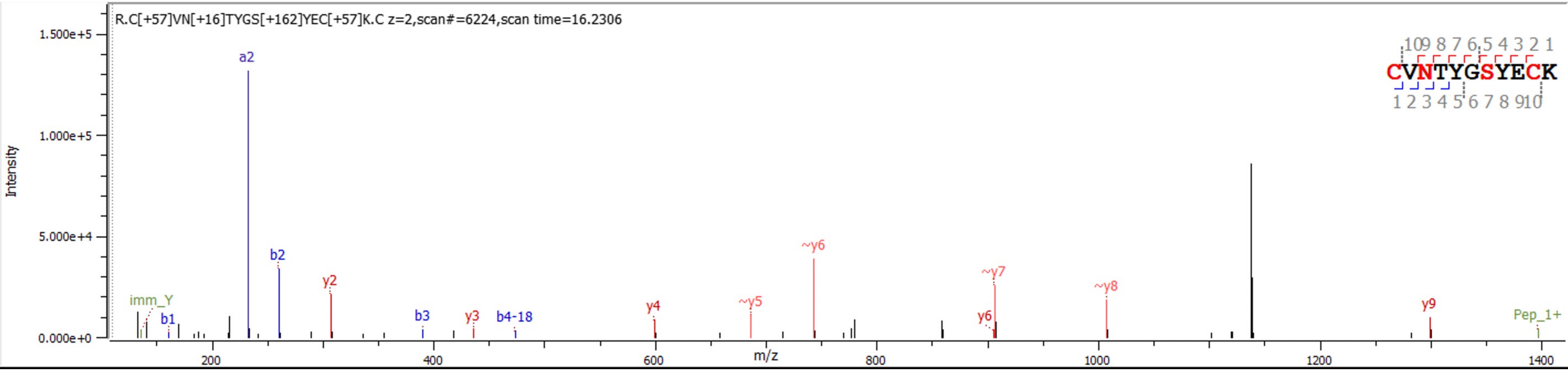

Figure S26
FBN1 EGF40

GSYTCECNDGFTASPNQDECLDNR

Hexose/Glucose

#	a obs.	b obs.	b-18 obs.	Seq.	y obs.	y++ obs.	#
1				G			24
2				S-Hex(1)			23
3				Y			22
4	543.2156	571.2147	553.2129	T			21
5		731.2474		C			20
6	832.2985	860.2814	842.2947	E			19
7				C			18
8				N			17
9				D			16
10				G	1723.7207		15
11				F			14
12				T	1519.649	760.3337	13
13				A	1418.5997	709.7968	12
14				S	1347.559	674.2845	11
15				P	1260.5281	630.7678	10
16				N	1163.478	582.2432	9
17				Q	1049.4337	525.222	8
18				D	921.3747		7
19				E	806.3516		6
20				C	677.3122		5
21				L	517.2739		4
22				D	404.1895		3
23				N	289.1622		2
24				R	175.1193		1

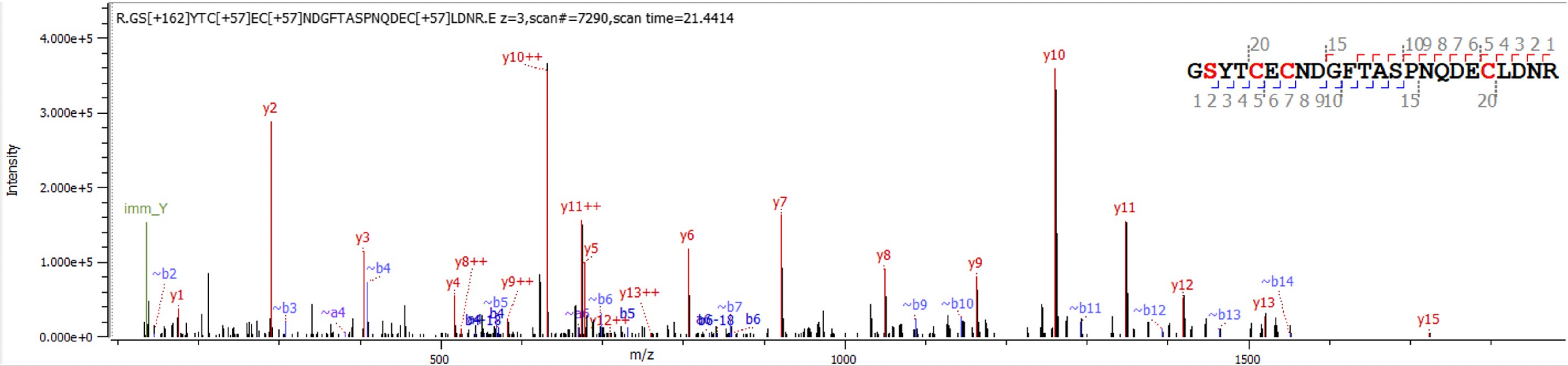

Figure S27
FBN1 EGF42

NTEGSYQCSCPK

● Hexose/Glucose

#	a obs.	b obs.	b-18 obs.	Seq.	y obs.	#
1				N-Oxidation		12
2	204.0981	232.0928	214.0831	T		11
3		361.135	343.1243	E		10
4			400.144	G		9
5				S-Hex(1)		8
6				Y	942.3771	7
7				Q	779.3163	6
8				C	651.2615	5
9				S	491.2274	4
10				C	404.1978	3
11				P	244.1658	2
12				K		1

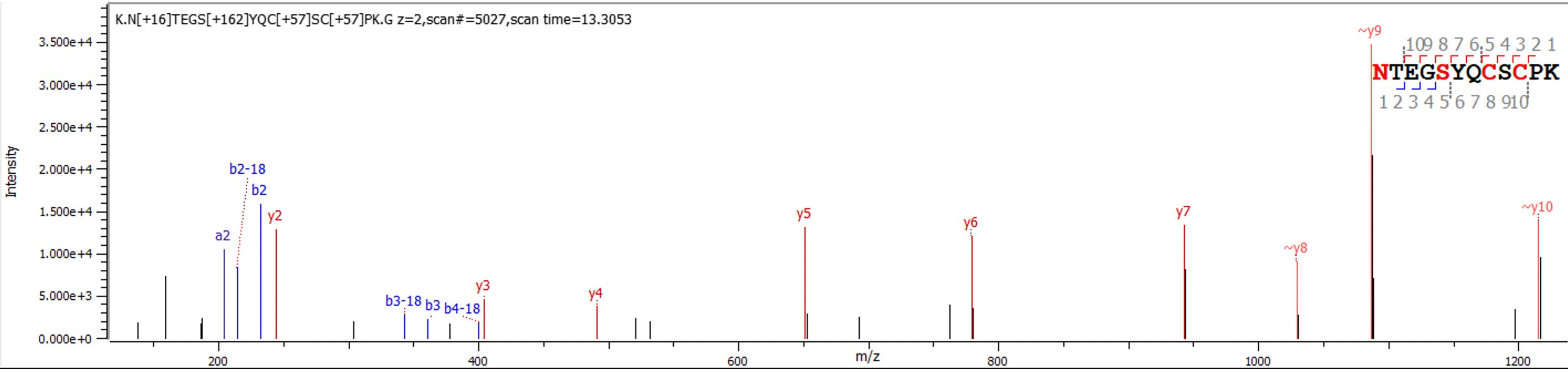

Figure S28
FBN1 EGF44

GICQNTPGSFTCECQR (hexose on S)

Hexose/Glucose

#	a obs.	b obs.	b-18 obs.	Seq.	y obs.	y++ obs.	#
1				G			16
2	143.118	171.113		I			15
3	303.1477	331.1436		C			14
4		459.2016		Q			13
5		589.2375		N-Oxidation			12
6	662.2887	690.2865	672.2766	T			11
7				P			10
8		844.3564		G			9
9				S-Hex(1)			8
10				F	1000.3979	500.7044	7
11				T	853.3292	427.1684	6
12				C	752.2816		5
13				E	592.2513		4
14				C	463.2077		3
15				Q	303.1774		2
16				R	175.119		1

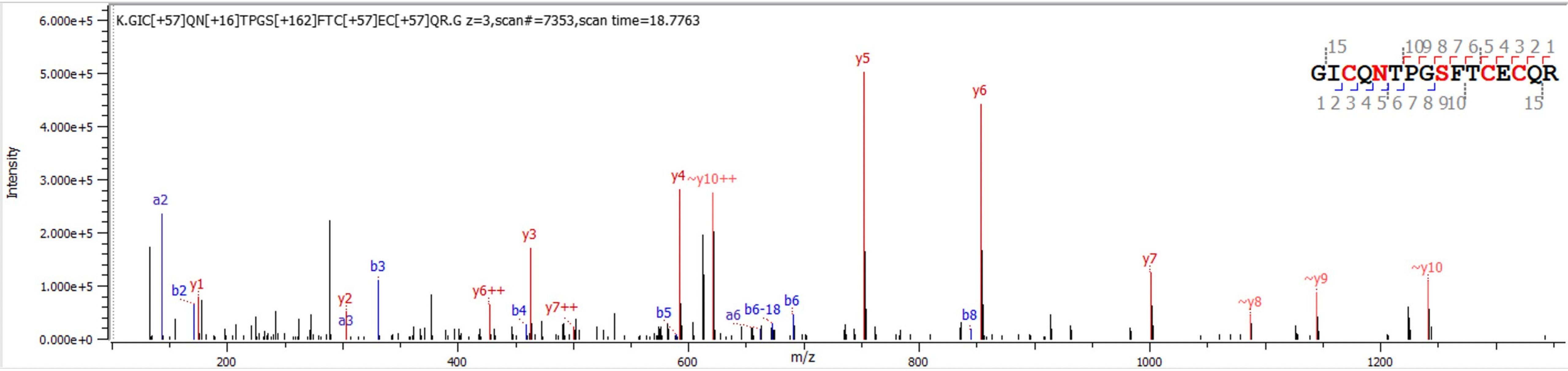

Figure S29
FBN1 EGF46

CSCPQGYLQHYQWNQCVDENECLSAHICGGASCHNTLG<u>S</u>YK

● Hexose/Glucose

#	a obs.	b obs.	b-18 obs.	b++ obs.	b_3+ obs.	Seq.	y obs.	y++ obs.	y_3+ obs.	y_4+ obs.	#
1		161.0373				C					41
2	220.0752	248.0701	230.0592			S					40
3	380.1053	408.1006	390.09			C				1205.9991	39
4		505.1497				P				1165.9963	38
5	605.2203	633.2068	615.2			Q					37
6		690.2219				G				1109.7335	36
7		853.2825				Y					35
8		966.3896	948.3944			L					34
9	1066.4385	1094.4448				Q					33
10						H					32
11		1394.5519				Y					31
12		1522.6364		761.8136		Q					30
13		1708.7046		854.8535		W					29
14				911.8792		N					28
15				975.899		Q					27
16				1055.9351		C		1559.1379	1039.7518		26
17				1105.4774	737.326	V			986.4165		25
18						D		1429.5957	953.3978		24
19				1227.5197		E					23
20						N		1307.5542			22
21				1349.059	899.7106	E		1250.5413			21
22						C		1186.0111			20
23						L					19
24						S		1049.4584			18
25						A					17
26						H		970.4316			16
27						I		901.8985			15
28						C		845.3446			14
29						G		765.3203			13
30						G					12
31						A		708.3229			11
32						S					10
33						C					9
34						H	1097.4846				8
35						N	960.4401				7
36						T	830.4101				6
37						L					5
38						G	616.2772				4
39						S-Hex(1)					3
40						Y	310.1766				2
41						K	147.1127				1

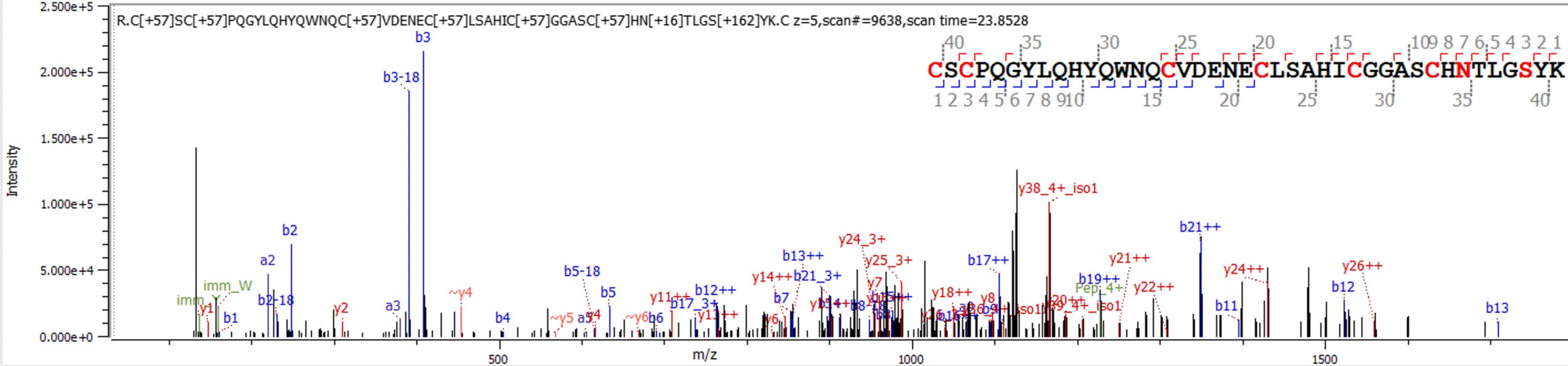

Figure S30
FBN2 EGF4

TGACQDVDECQAIPGICQGGNCINTVG<u>S</u>FECR (● on S)

● Hexose/Glucose

#	a obs.	b obs.	b-18 obs.	b++ obs.	Seq.	y obs.	y++ obs.	#
1					T			32
2					G			31
3		230.1123			A			30
4		390.1454			C			29
5		518.2031	500.1978		Q			28
6		633.2311	615.2173		D			27
7	704.3043	732.2983	714.2879		V			26
8	819.3136	847.3256	829.3175		D			25
9	948.3764	976.3671	958.3589	488.6878	E			24
10		1136.4124			C			23
11		1264.4564	1246.4603		Q			22
12	1307.5043	1335.4923	1317.4866	668.2513	A			21
13	1420.5897	1448.5779	1430.5704		I			20
14					P	2287.9602	1144.4926	19
15					G			18
16		1715.7335			I			17
17					C			16
18					Q	1860.7782	930.881	15
19					G	1732.7297		14
20					G			13
21					N			12
22					C	1504.6277		11
23					I	1344.6082		10
24					N	1231.5232		9
25					T	1117.4813		8
26					V	1016.4361		7
27					G	917.3674		6
28					S-Hex(1)	860.3431		5
29					F	611.2602		4
30					E	464.1916		3
31					C	335.1497		2
32					R	175.1191		1

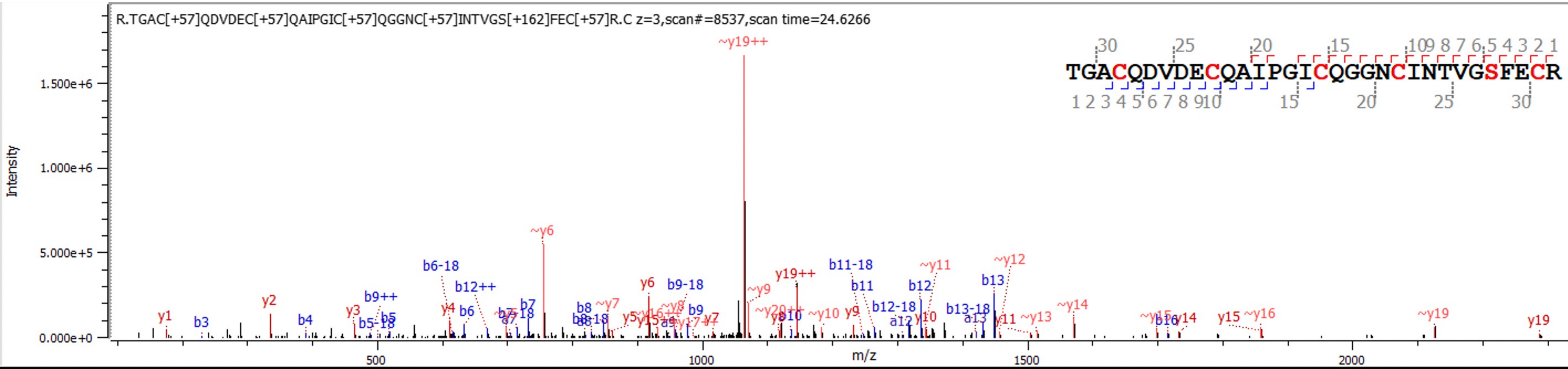

Figure S31
FBN2 EGF5

CEDIDECSIIPGICETGECSNTVGSYFCVCPR

● Hexose/Glucose

#	a obs.	b obs.	b-18 obs.	Seq.	y obs.	y++ obs.	#
1				C			32
2	262.0862	290.0806	272.07	E			31
3	377.1104	405.1078	387.0974	D			30
4	490.1984	518.1918	500.1825	I			29
5	605.2229	633.2178	615.205	D			28
6	734.2592	762.2585	744.2493	E			27
7	894.2983	922.2924	904.2744	C			26
8	981.3371	1009.3245	991.3101	S			25
9	1094.4155	1122.4088	1104.3955	I			24
10	1207.4775	1235.4922	1217.4823	I			23
11		1332.5752		P		1356.5674	22
12	1361.5597	1389.5698		G			21
13		1502.6477		I			20
14				C			19
15				E			18
16				T			17
17				G			16
18				E			15
19				C	1868.7478		14
20				S	1708.7183		13
21				N	1621.6831		12
22				T	1507.6483		11
23				V	1406.5864		10
24				G	1307.5336		9
25				S-Hex(1)	1250.5148		8
26				Y	1001.4347		7
27				F	838.3713		6
28				C	691.3024		5
29				V	531.2711		4
30				C	432.2026		3
31				P	272.172		2
32				R	175.1193		1

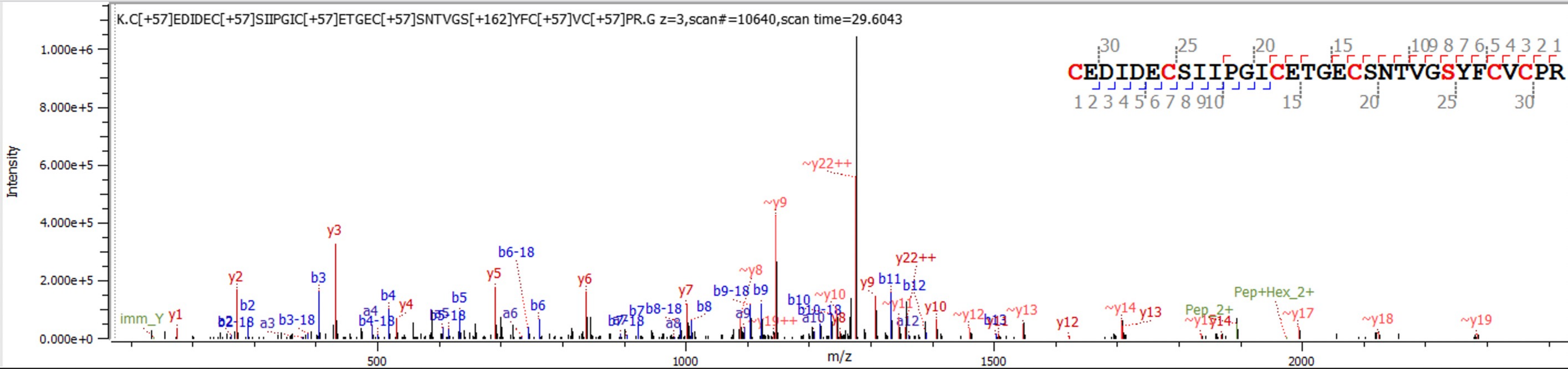

Figure S32
FBN2 EGF6

CIPTVSSYR

● Hexose/Glucose

#	a obs.	b obs.	b-18 obs.	Seq.	y obs.	y++ obs.	#
1		161.0381		C			9
2	246.1273	274.1222		I			8
3				P	971.4683	486.2388	7
4		472.232	454.2198	T	874.4178		6
5				V	773.368		5
6				S	674.3002		4
7				S-Hex(1)	587.2675		3
8				Y	338.1837		2
9				R	175.1192		1

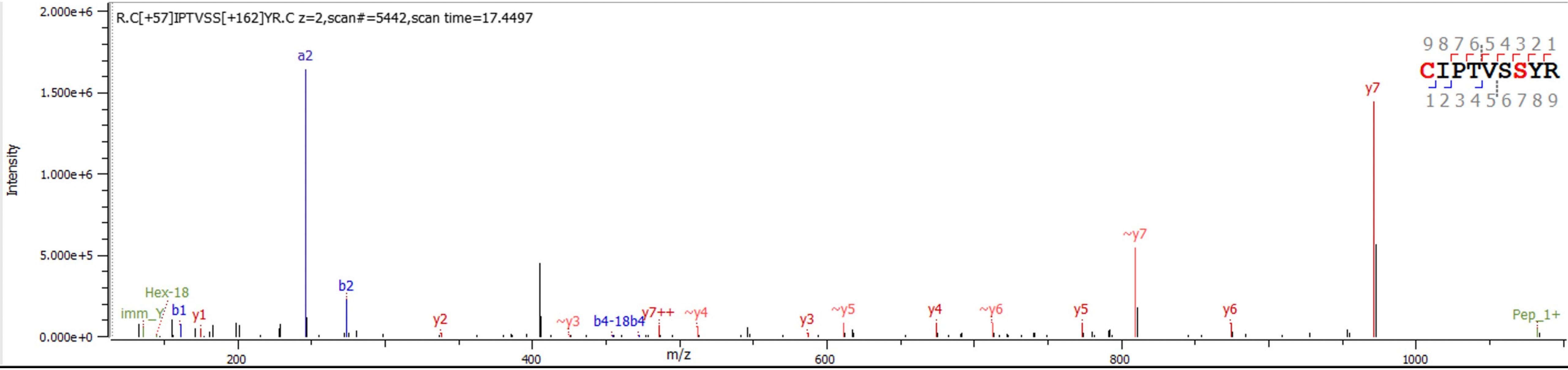

Figure S33
FBN2 EGF7

QDANGDCIDVDECTSNPCTNGDCVNTPG<u>S</u>YYCK

● Hexose/Glucose

*2 hexoses were detected on this peptide. The hexose at position 15 is a possible POGLUT1 modification site based on the consensus sequence.

#	a obs.	b obs.	b-18 obs.	Seq.	y obs.	y++ obs.	#
1				Q			33
2	216.098	244.0925		D			32
3		315.13		A			31
4		429.1703	411.1636	N			30
5				G			29
6		601.2219	583.2133	D			28
7		761.2737	743.2595	C			27
8	846.3342			I			26
9		989.373	971.3579	D			25
10		1088.4282	1070.421	V			24
11			1185.4463	D			23
12		1332.5135		E			22
13				C			21
14			1575.5696	T			20
15				S-Hex(1)			19
16				N			18
17				P		1077.9131	17
18				C			16
19				T			15
20				N			14
21				G			13
22				D			12
23				C			11
24				V			10
25				N	1251.5166		9
26				T			8
27				P	1036.4304		7
28				G			6
29				<u>S</u>-Hex(1)			5
30				Y	633.2742		4
31				Y	470.2077		3
32				C	307.1435		2
33				K	147.1127		1

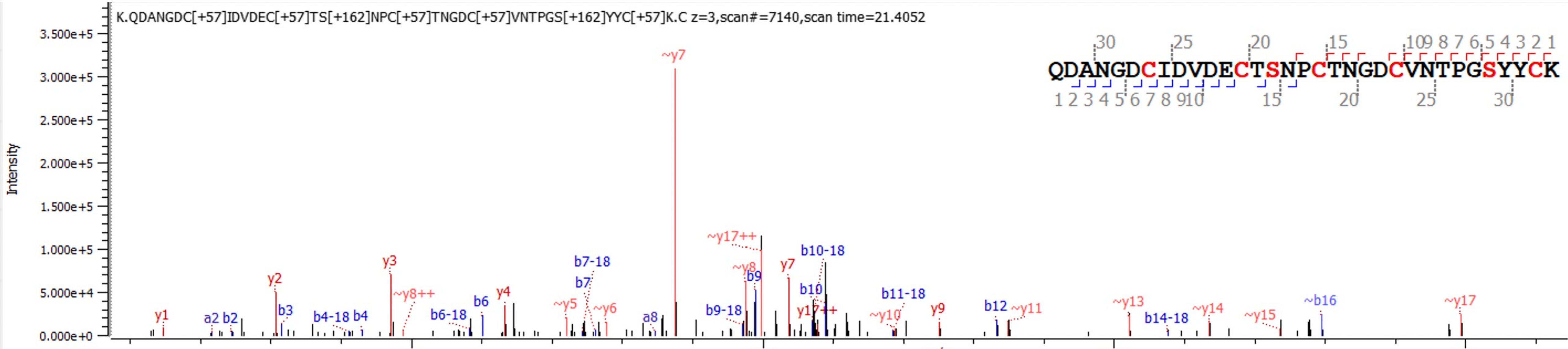

Figure S34
FBN2 EGF8

CVNTDG<u>S</u>FQCICNAGFELTTDGK

● Hexose/Glucose

#	a obs.	b obs.	b-18 obs.	Seq.	y obs.	y++ obs.	#
1		161.0375		C			23
2	232.1116	260.1069		V			22
3	346.1554	374.1487		N			21
4		475.1948	457.1801	T			20
5	562.2198	590.2238	572.2155	D			19
6	619.2337	647.2529	629.2357	G			18
7				S-Hex(1)			17
8				F			16
9				Q			15
10				C	1585.6974		14
11				I	1425.6661		13
12				C	1312.5845	656.796	12
13				N	1152.5712		11
14				A	1038.519		10
15				G	967.4744		9
16				F	910.4639		8
17				E	763.3836		7
18				L	634.3408		6
19				T	521.2573		5
20				T	420.2091		4
21				D	319.1618		3
22				G	204.1345		2
23				K	147.1125		1

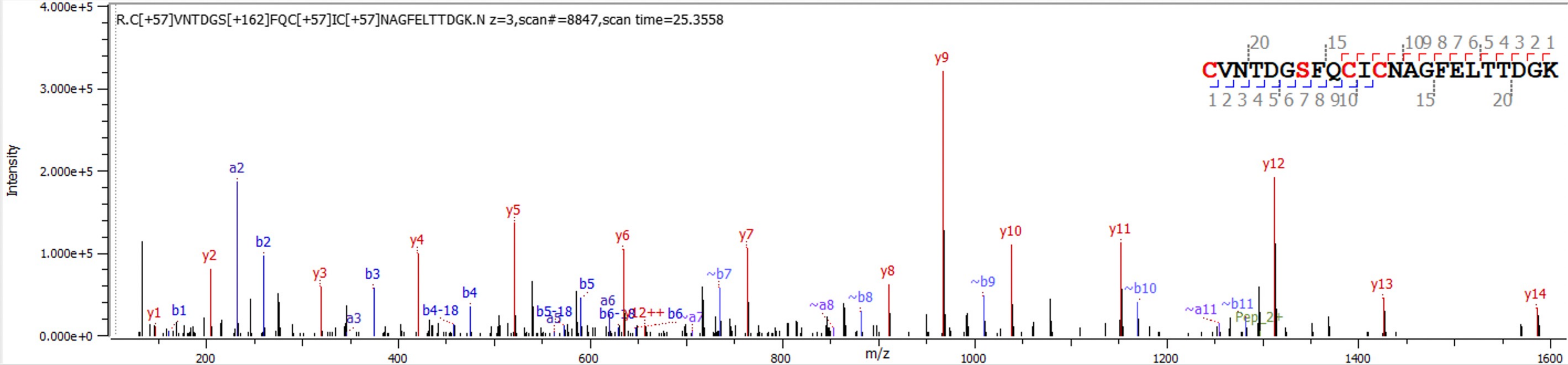

Figure S35
FBN2 EGF9

CVNTDGNCVDHDECTTTNMCLNGMCINEDGSFK

● Hexose/Glucose

#	a obs.	b obs.	b-18 obs.	b++ obs.	Seq.	y obs.	#
1					N		27
2	247.0869	275.0807			C		26
3		374.1494			V		25
4		489.1766			D		24
5	598.2435	626.2326	608.2151		H		23
6		741.2628			D		22
7	842.3076	870.304	852.2859		E		21
8	1002.3488	1030.337		515.6748	C		20
9	1103.3989	1131.3842	1113.3708	566.1967	T		19
10		1232.4402	1214.4227	616.7217	T		18
11		1333.4785	1315.4686	667.2436	T		17
12		1447.52	1429.5177	724.2625	N		16
13	1550.5658	1578.5642	1560.5658	789.7857	M		15
14		1738.5929			C	1806.7437	14
15		1851.6709		926.3484	L	1646.6891	13
16		1965.7045		983.3674	N	1533.6146	12
17				1011.8767	G	1419.573	11
18		2153.7864			M	1362.5421	10
19					C		9
20					I	1071.4756	8
21					N	958.3998	7
22					E	844.361	6
23					D		5
24					G	600.2911	4
25					S-Hex(1)	543.2679	3
26					F	294.1816	2
27					K	147.113	1

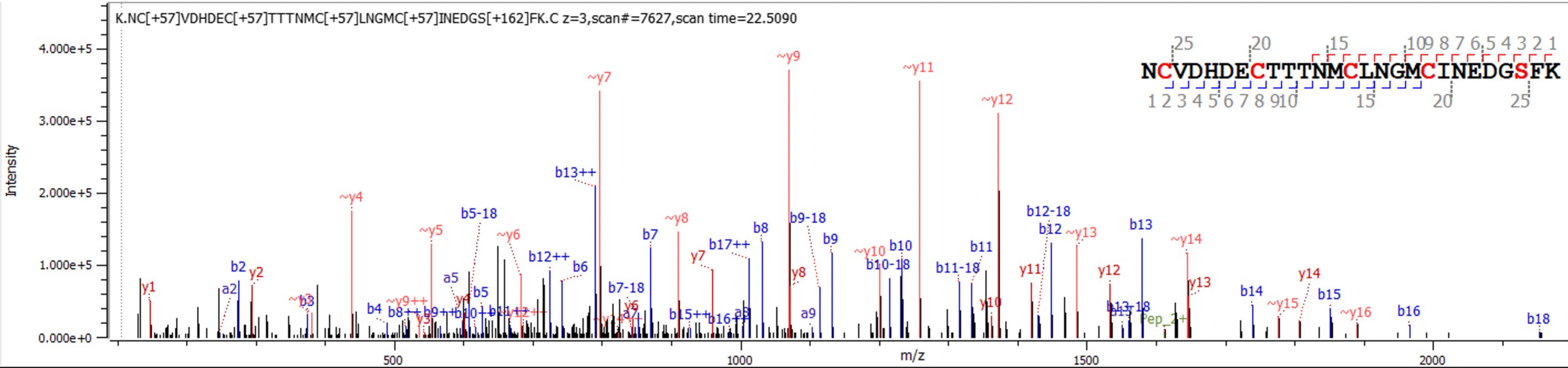

Figure S36
FBN2 EGF10

YCTDVDECQTPGICMNGHCINSEGSFR

● Hexose/Glucose

#	a obs.	b obs.	b-18 obs.	b++ obs.	Seq.	y obs.	y++ obs.	#
1		164.0699			Y			27
2	296.1068	324.1016			C			26
3		425.1483	407.1418		T			25
4		540.1765	522.1666		D		1473.1068	24
5	611.2488	639.2433	621.2297		V			23
6		754.2711	736.2569		D		1366.054	22
7	855.314	883.3144	865.303		E		1308.5536	21
8		1043.3542			C		1244.0326	20
9		1171.4066			Q		1163.9882	19
10		1272.4536	1254.4498		T	2198.9048	1099.9705	18
11					P	2097.8931	1049.4471	17
12					G	2000.826	1000.9108	16
13				770.3196	I		972.4041	15
14					C	1830.7192		14
15				915.8629	M	1670.6769	835.8436	13
16					N	1539.6418		12
17					G	1425.6005		11
18					H	1368.5845		10
19					C	1231.5255		9
20					I	1071.4883		8
21					N	958.415		7
22					S	844.3694		6
23					E	757.3365		5
24					G	628.2946		4
25				1524.6161	S-Hex(1)	571.2764		3
26					F	322.187		2
27					R	175.1192		1

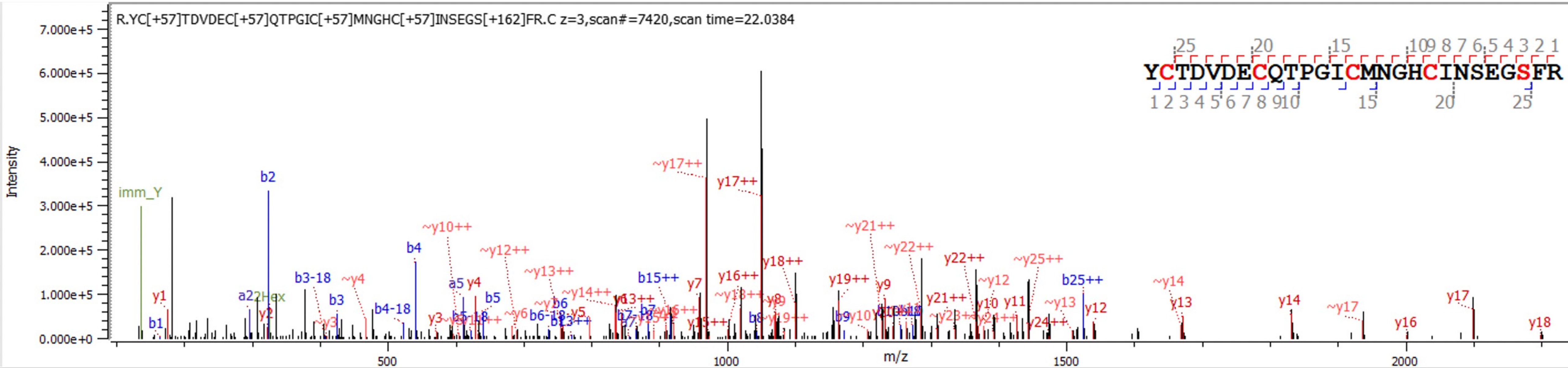

Figure S37
FBN2 EGF12

NTPGSYSCTCPPGYVFR

Hexose/Glucose

#	a obs.	b obs.	b-18 obs.	Seq.	y obs.	y++ obs.	#
1				N			17
2	188.1017	216.0965	198.0853	T			16
3			295.1383	P	1909.7609		15
4			352.1598	G			14
5				S-Hex(1)	1755.696		13
6				Y	1506.6394		12
7				S	1343.5784		11
8				C	1256.5468		10
9				T	1096.5165		9
10				C	995.4695		8
11				P	835.4399	418.2217	7
12				P	738.3879		6
13				G	641.3368		5
14				Y	584.3163		4
15				V	421.2525		3
16				F	322.1852		2
17				R	175.1178		1

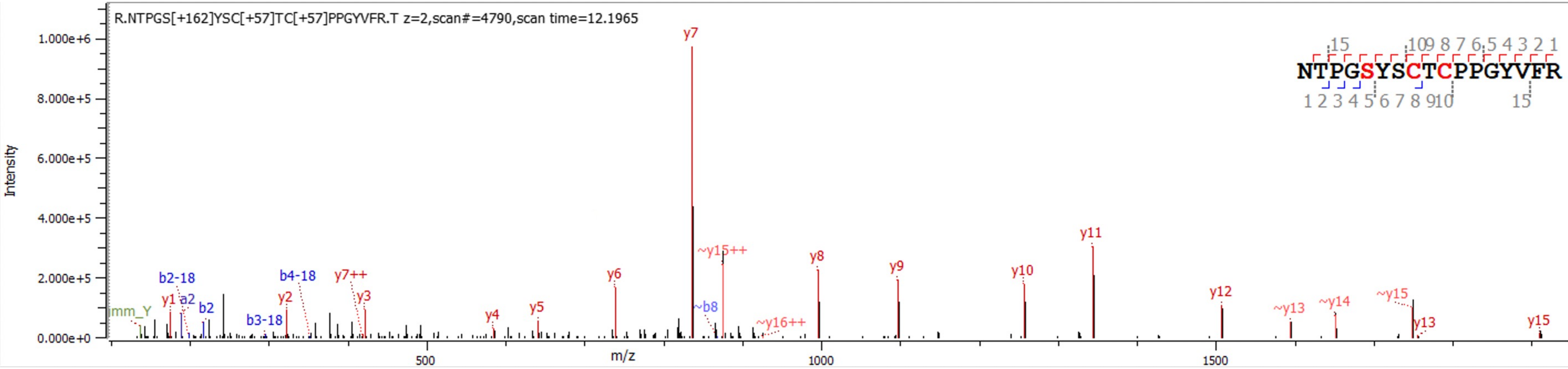

Figure S38
FBN2 EGF13

NNLGSFNCECSPGSK

Hexose/Glucose

#	a obs.	b obs.	Seq.	y obs.	#
1			N		15
2	201.0978	229.0934	N		14
3		342.1781	L		13
4		399.1983	G	1491.5453	12
5			S-Hex(1)		11
6			F	1185.4628	10
7			N	1038.4005	9
8			C	924.3493	8
9			E	764.3273	7
10			C	635.2842	6
11			S	475.2523	5
12			P	388.2192	4
13			G	291.1663	3
14			S	234.1454	2
15			K	147.1128	1

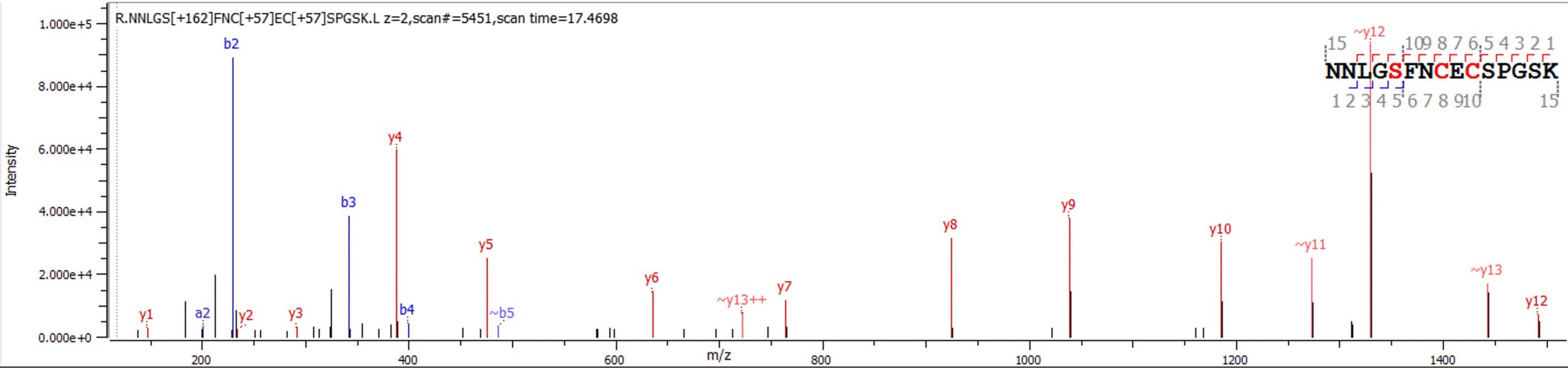

Figure S39
FBN2 EGF14

GSFHCECPEGLTLDGTGR

Hexose/Glucose

#	b obs.	b-18 obs.	Seq.	y obs.	y++ obs.	#
1			G			18
2			S-Hex(1)			17
3			F		924.8995	16
4	591.2318	573.2158	H		851.3618	15
5			C	1564.6868		14
6	880.2927	862.2808	E	1404.6373		13
7	1040.3407		C			12
8			P	1115.5723	558.2894	11
9			E	1018.5161		10
10	1323.4642		G	889.4744		9
11			L	832.4509		8
12			T	719.3692	360.1867	7
13			L	618.3209	309.6638	6
14			D	505.2368		5
15			G	390.2096		4
16			T	333.1884		3
17			G	232.1413		2
18			R	175.1196		1

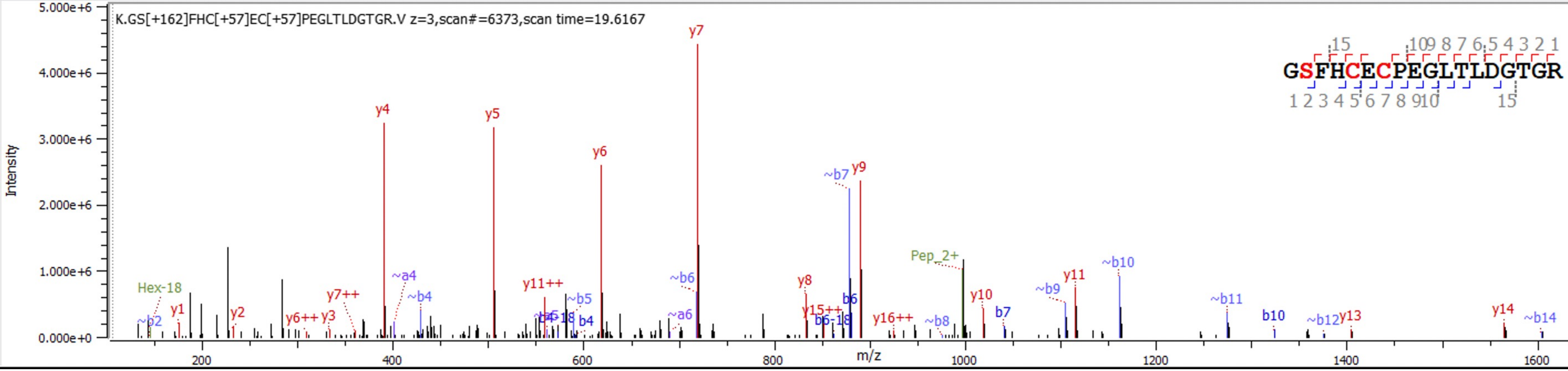

Figure S40
FBN2 EGF15

NTIGSFK

● Hexose/Glucose

#	a obs.	b obs.	b-18 obs.	Seq.	y obs.	#
1				N		7
2	188.1033	216.0978	198.0871	T		6
3			311.1724	I	713.371	5
4				G	600.2916	4
5				S-Hex(1)		3
6				F	294.1812	2
7				K	147.1124	1

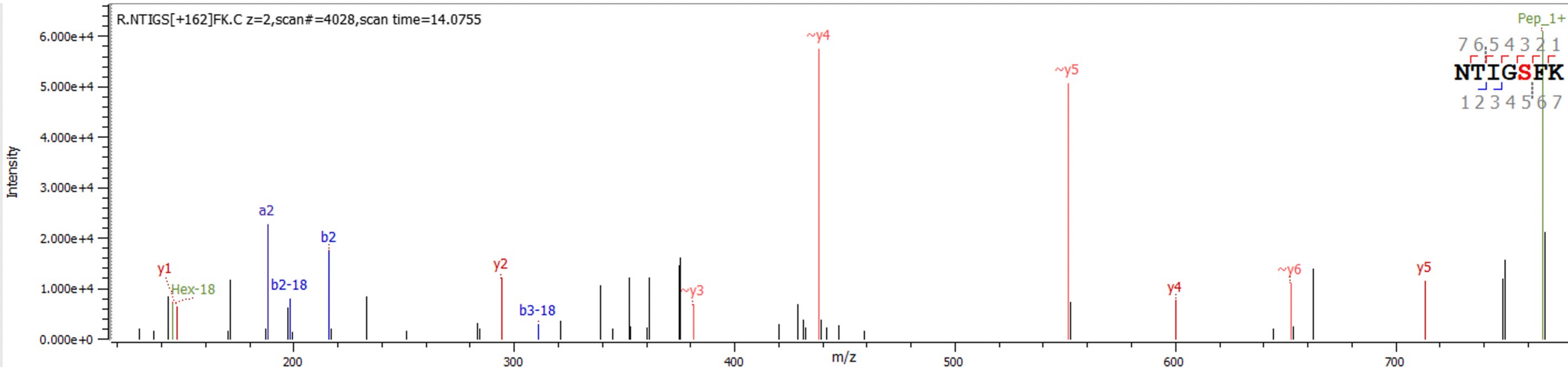

Figure S41
FBN2 EGF17

GGTCVNTEGSFQCDCPLGHELSPSREDCVDINECSLSDNLCR

● Hexose/Glucose

#	a obs.	b obs.	b-18 obs.	b++ obs.	b_3+ obs.	Seq.	y obs.	y++ obs.	y_3+ obs.	#
1						G				42
2						G				41
3	188.1012	216.0966	198.086			T				40
4	348.131	376.1258	358.1156			C				39
5	447.1987	475.1934	457.1829			V				38
6	561.2408	589.2332	571.2254			N				37
7	662.2863	690.2862	672.2778			T				36
8	791.3381	819.3281	801.3145			E				35
9			858.3339			G				34
10						S-Hex(1)				33
11						F				32
12						Q				31
13						C		1804.231		30
14						D		1724.2115		29
15						C		1666.708	1111.4719	28
16						P		1586.6921	1058.1412	27
17						L		1538.1595		26
18						G				25
19				1120.4457		H				24
20						E		1384.5906		23
21						L		1320.083		22
22						S		1263.5334		21
23						P		1220.0164		20
24						S				19
25						R		1127.9741		18
26						E				17
27						D	1969.7848			16
28						C	1854.7616			15
29						V	1694.7251			14
30						D	1595.6685			13
31						I	1480.6398			12
32						N	1367.5599			11
33					1295.2067	E	1253.5121			10
34						C	1124.4746			9
35						S	964.4451			8
36						L	877.4152			7
37						S	764.3301			6
38						D	677.3024			5
39						N	562.2728			4
40						L	448.2361			3
41						C	335.1475			2
42						R	175.1178			1

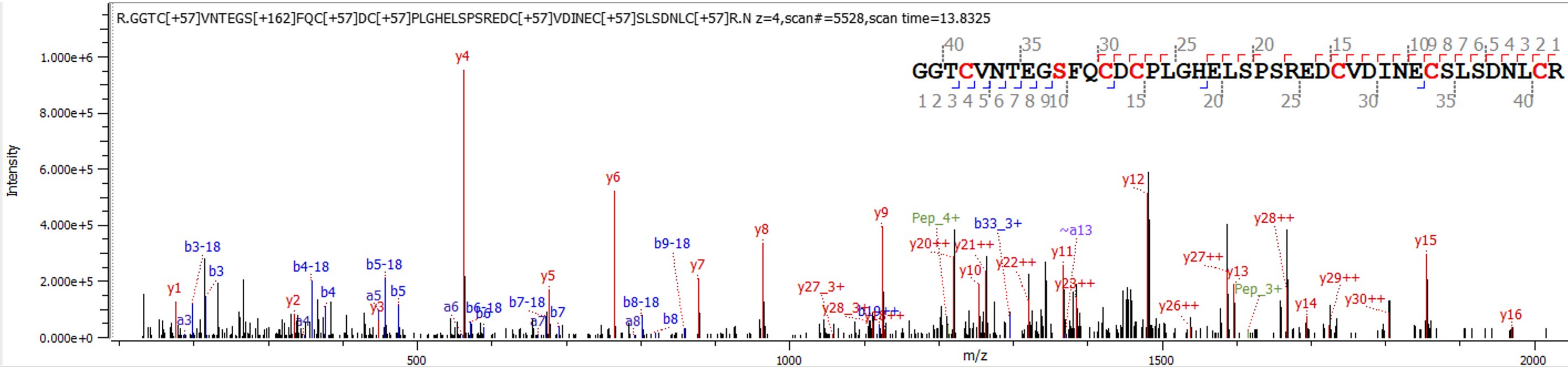

Figure S42
FBN2 EGF18

CVNMIG<u>T</u>YQCSCNPGYQATPDR

● Hexose/Glucose

#	a obs.	b obs.	Seq.	y obs.	y++ obs.	#
1			C			22
2	232.1116	260.1058	V			21
3		374.1487	N			20
4		505.1917	M			19
5	590.2829	618.279	I			18
6			G			17
7			T-Hex(1)			16
8			Y			15
9			Q			14
10			C	1525.602		13
11			S	1365.593		12
12			C	1278.552		11
13			N	1118.517		10
14			P	1004.479	502.7441	9
15			G	907.43		8
16			Y	850.4069		7
17			Q	687.3428		6
18			A	559.2833		5
19			T	488.2472		4
20			P	387.1985		3
21			D	290.1497		2
22			R	175.1191		1

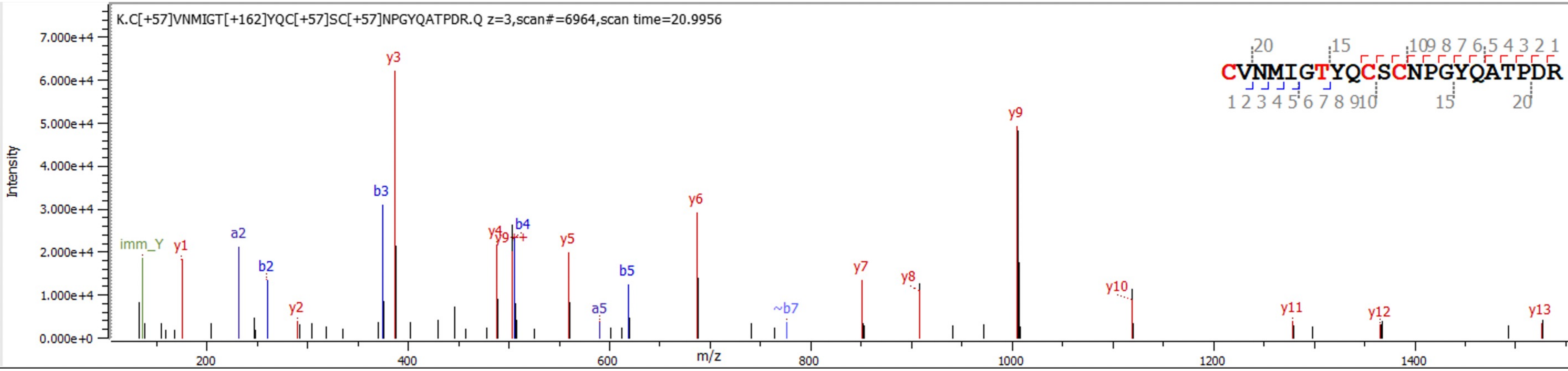

Figure S43
FBN2 EGF19

QGCTDIDECMIMNGGCDTQCTNSEG<u>S</u>YECSCSEGYALMPDGR

● Hexose/Glucose

#	b obs.	b-18 obs.	Seq.	y obs.	#
1			Q		42
2			G		41
3			C		40
4	447.165		T		39
5	562.1974		D		38
6	675.2762	657.2692	I		37
7	790.3046	772.2922	D		36
8	919.3463		E		35
9	1079.3699		C		34
10	1210.4298		M		33
11	1323.501	1305.4912	I		32
12	1454.519		M		31
13			N-Oxiadtion		30
14			G		29
15			G		28
16			C		27
17			D		26
18			T		25
19			Q		24
20			C		23
21			T		22
22			N		21
23			S		20
24			E		19
25			G		18
26			S-Hex(1)		17
27			Y		16
28			E		15
29			C		14
30			S		13
31			C		12
32			S		11
33			E		10
34			G	979.4661	9
35			Y	922.45	8
36			A	759.3838	7
37			L	688.3471	6
38			M	575.2606	5
39			P	444.2202	4
40			D		3
41			G	232.1406	2
42			R		1

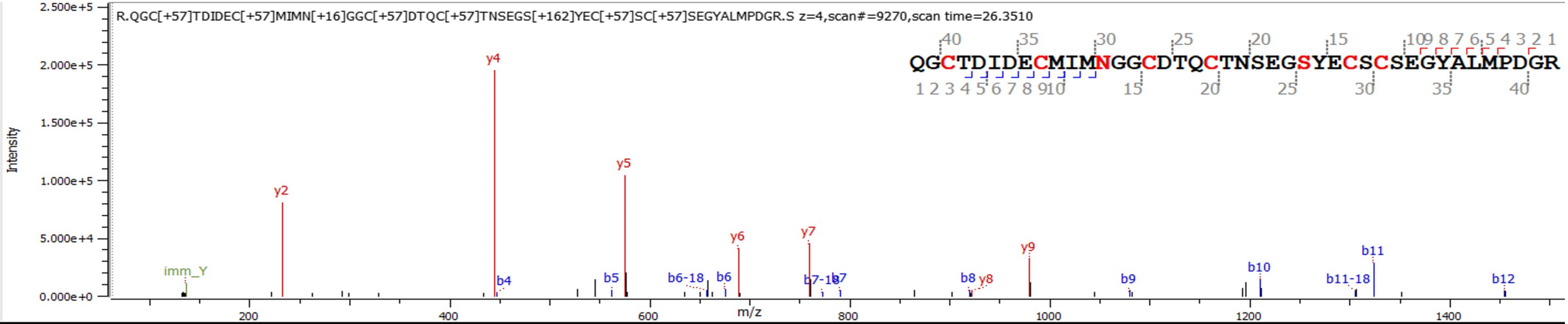

Figure S44
FBN2 EGF21

GSFICHCQLGYSVK

● Hexose/Glucose

*Hexose is assigned to wrong residue. This is not uncommon to find with certain peptides. Double knockout data confirmed this modification is being added by POGLUT2 and 3.

#	a obs.	b obs.	b-18 obs.	Seq.	y obs.	y++ obs.	#
1				G			14
2		145.0603		S			13
3	264.1356	292.1289	274.1192	F			12
4	377.2192	405.2142		I			11
5	537.2566			C			10
6	674.3143	702.3027		H		627.2852	9
7		862.331		C			8
8		990.3979		Q	956.4723		7
9		1103.4694		L			6
10		1160.4967		G			5
11		1323.5577		Y			4
12				S-Hex(1)			3
13				V	246.1813		2
14				K	147.113		1

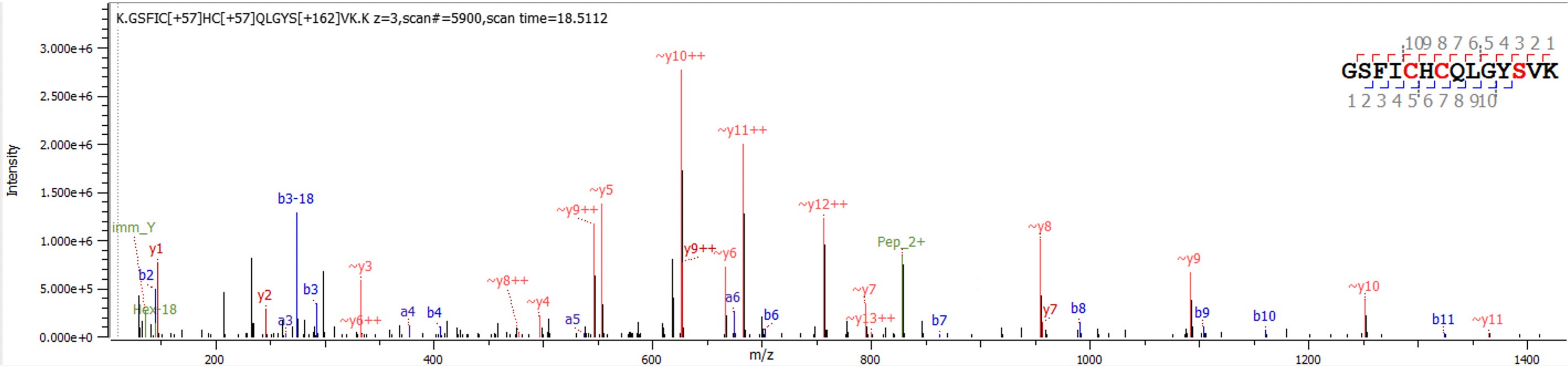

Figure S45
FBN2 EGF22

KGTTGCTDVDECEIGAHNCDMHASCLNIPGSFK (hexose on underlined S)

● Hexose/Glucose

#	a obs.	b obs.	b-18 obs.	b++ obs.	b_3+ obs.	b_4+ obs.	Seq.	y obs.	y++ obs.	y_3+ obs.	#
1							K				33
2		186.1226					G				32
3		287.1714	269.1536				T				31
4		388.2191	370.2071				T				30
5		445.2422	427.2302				G				29
6		605.2719	587.2629				C				28
7		706.3179	688.3069				T				27
8		821.3455	803.3344	411.177			D				26
9	892.4204	920.4147	902.4014	460.7103			V			1008.4225	25
10		1035.4348	1017.4296	518.2167			D			975.4005	24
11		1164.4812		582.7457			E				23
12		1324.5238		662.7588			C			894.0423	22
13		1453.5607		727.2842			E			840.6972	21
14		1566.6188					I			797.6859	20
15							G		1139.481	759.9953	19
16		1694.6879		847.8569			A				18
17				916.3809			H		1075.463		17
18				973.4066			N				16
19							C				15
20				1110.9335			D				14
21				1176.4375			M		812.3672		13
22				1244.986	830.3256		H				12
23				1280.5149	854.0022		A				11
24					883.031		S				10
25					936.3605		C				9
26					974.069		L				8
27					1012.0724		N	924.4719			7
28					1049.7485	787.5662	I	810.4271	405.717		6
29							P	697.3409			5
30							G	600.2932			4
31							S-Hex(1)	543.2667			3
32							F	294.1826			2
33							K	147.1128			1

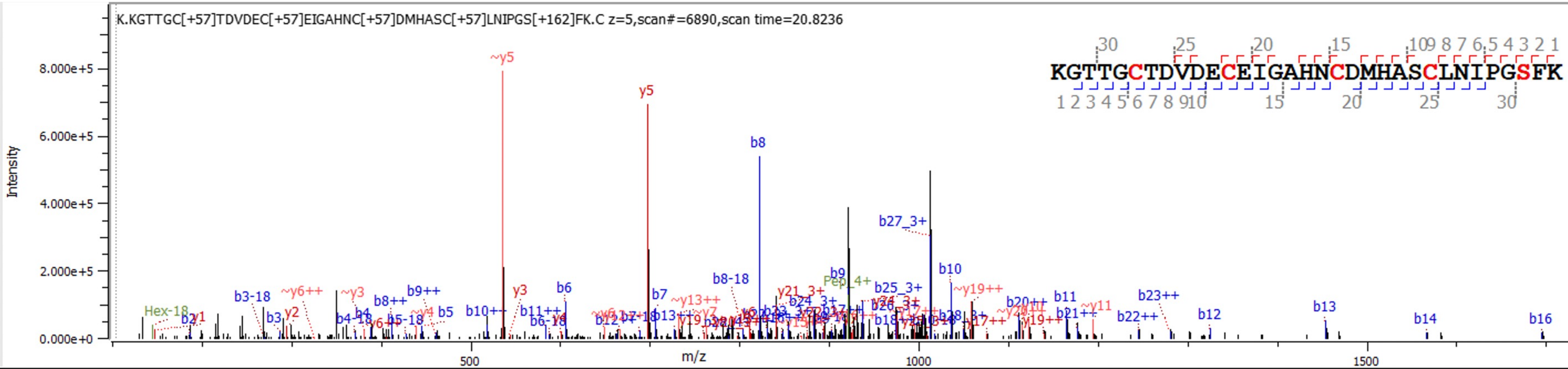

Figure S46
FBN2 EGF27

CQELPGLCQGGNCINTFG<u>S</u>FQCECPQGYYLSE

● Hexose/Glucose

*Hexose is assigned to wrong residue. This is not uncommon to find with certain peptides.

#	a calc.	a obs.	b calc.	b obs.	b-18 calc.	b-18 obs.	b++ calc.	Seq.	y calc.	y obs.	y++ calc.	#
1	133.043		161.0379		143.0274		81.0226	C				32
2	261.1016		289.0965	289.0963	271.0859	271.0852	145.0519	Q	3775.5655		1888.2864	31
3	390.1442		418.1391	418.1389	400.1285	400.1259	209.5732	E	3647.5069		1824.2571	30
4	503.2282	503.2277	531.2231	531.2238	513.2126	513.2139	266.1152	L	3518.4643		1759.7358	29
5	600.281		628.2759		610.2653		314.6416	P	3405.3802		1703.1937	28
6	657.3025		685.2974	685.2983	667.2868		343.1523	G	3308.3275		1654.6674	27
7	770.3865	770.3929	798.3814	798.3824	780.3709		399.6944	L	3251.306		1626.1566	26
8	930.4172	930.423	958.4121	958.4083	940.4015		479.7097	C	3138.2219		1569.6146	25
9	1058.4757		1086.4706		1068.4601		543.739	Q	2978.1913		1489.5993	24
10	1115.4972		1143.4921		1125.4815		572.2497	G	2850.1327		1425.57	23
11	1172.5187		1200.5136		1182.503		600.7604	G	2793.1113		1397.0593	22
12	1286.5616		1314.5565		1296.5459		657.7819	N	2736.0898		1368.5485	21
13	1446.5922		1474.5871	1474.6031	1456.5766		737.7972	C	2622.0469		1311.5271	20
14	1559.6763		1587.6712	1587.677	1569.6606		794.3392	I	2462.0162		1231.5118	19
15	1673.7192		1701.7141		1683.7036		851.3607	N	2348.9322		1174.9697	18
16	1936.8197		1964.8146		1946.8041		982.911	T-Hex(1)	2234.8892		1117.9483	17
17	2083.8881		2111.883		2093.8725		1056.4452	F	1971.7887		986.398	16
18	2140.9096		2168.9045		2150.8939		1084.9559	G	1824.7203		912.8638	15
19	2227.9416		2255.9365		2237.926		1128.4719	S	1767.6989		884.3531	14
20	2375.01		2403.0049		2384.9944		1202.0061	F	1680.6668		840.8371	13
21	2503.0686		2531.0635		2513.053		1266.0354	Q	1533.5984		767.3029	12
22	2663.0992		2691.0942		2673.0836		1346.0507	C	1405.5399		703.2736	11
23	2792.1418		2820.1367		2802.1262		1410.572	E	1245.5092		623.2582	10
24	2952.1725		2980.1674		2962.1568		1490.5873	C	1116.4666	1116.4614	558.7369	9
25	3049.2252		3077.2201		3059.2096		1539.1137	P	956.436	956.4371	478.7216	8
26	3177.2838		3205.2787		3187.2682		1603.143	Q	859.3832		430.1952	7
27	3234.3053		3262.3002		3244.2896		1631.6537	G	731.3246	731.3266	366.166	6
28	3397.3686		3425.3635		3407.353		1713.1854	Y	674.3032		337.6552	5
29	3560.4319		3588.4268		3570.4163		1794.7171	Y	511.2399	511.24	256.1236	4
30	3673.516		3701.5109		3683.5003		1851.2591	L	348.1765	348.1754	174.5919	3
31	3760.548		3788.5429		3770.5324		1894.7751	S	235.0925	235.0923	118.0499	2
32								E	148.0604	148.0604	74.5339	1

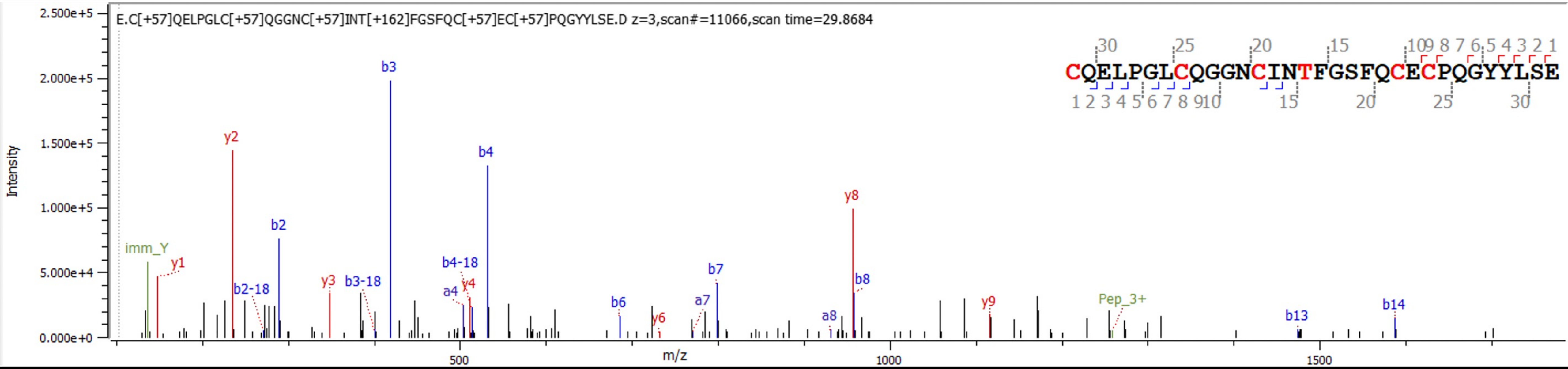

Figure S47
FBN2 EGF30

NADCINSPG<u>S</u>YR (● on S)

● Hexose/Glucose

#	a obs.	b obs.	b-18 obs.	Seq.	y obs.	#
1				N		12
2	158.0922	186.0871		A		11
3		301.1139	283.1024	D	1330.563	10
4	433.1502	461.1437		C	1215.532	9
5	546.2355	574.2266	556.2144	I	1055.5	8
6	660.2762	688.2689		N	942.4152	7
7		775.3053	757.2961	S	828.3714	6
8				P	741.3423	5
9				G		4
10				S-Hex(1)		3
11				Y	338.1813	2
12				R	175.1186	1

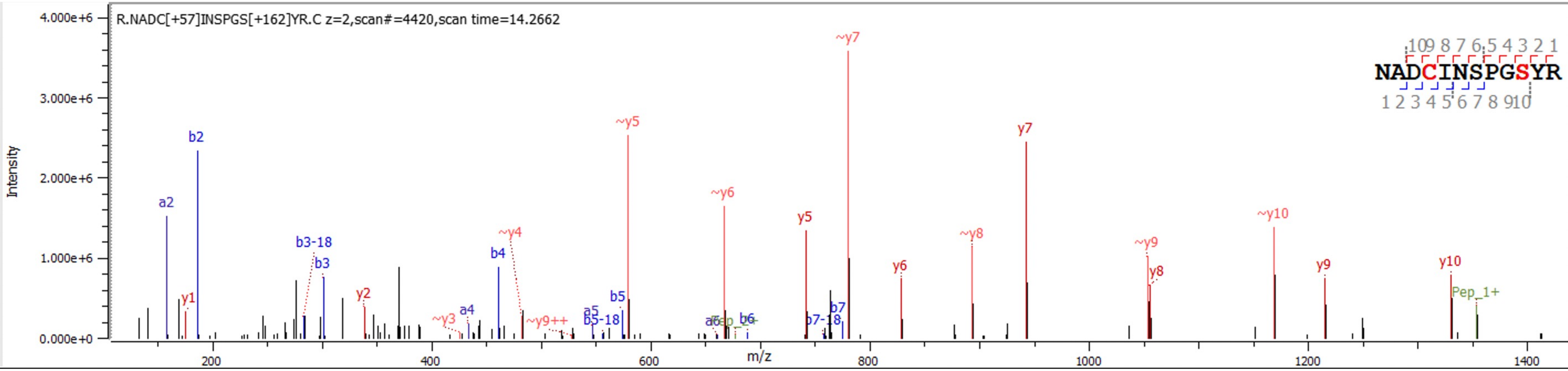

Figure S48
FBN2 EGF32

NTVGSYNCLCYPGFELTHNNDCLDIDECSSFFGQVCR

Hexose/Glucose

*Hexose is assigned to wrong residue. This is not uncommon to find with certain peptides. Double knockout data confirmed this modification is being added by POGLUT2 and 3.

#	a obs.	b obs.	b-18 obs.	b++ obs.	Seq.	y obs.	y++ obs.	#
1					N			37
2	188.1019	216.0974	198.0856		T			36
3		315.166	297.1552		V			35
4		372.1873			G			34
5			441.2087		S			33
6			604.2746		Y			32
7		736.3305			N			31
8		896.3563			C			30
9					L			29
10		1169.4764	1151.4716		C			28
11		1332.5367	1314.5256		Y			27
12					P			26
13					G			25
14					F			24
15					E			23
16					L			22
17					T			21
18					H			20
19					N			19
20					N			18
21					D			17
22					C			16
23					L			15
24				1423.0879	D			14
25					I			13
26					D			12
27					E			11
28					C			10
29					S-Hex(1)			9
30					S	1000.4688		8
31					F	913.4329	457.225	7
32					F	766.3651	383.6861	6
33					G	619.2971		5
34					Q	562.2747		4
35					V	434.2177		3
36					C	335.1487		2
37					R	175.1187		1

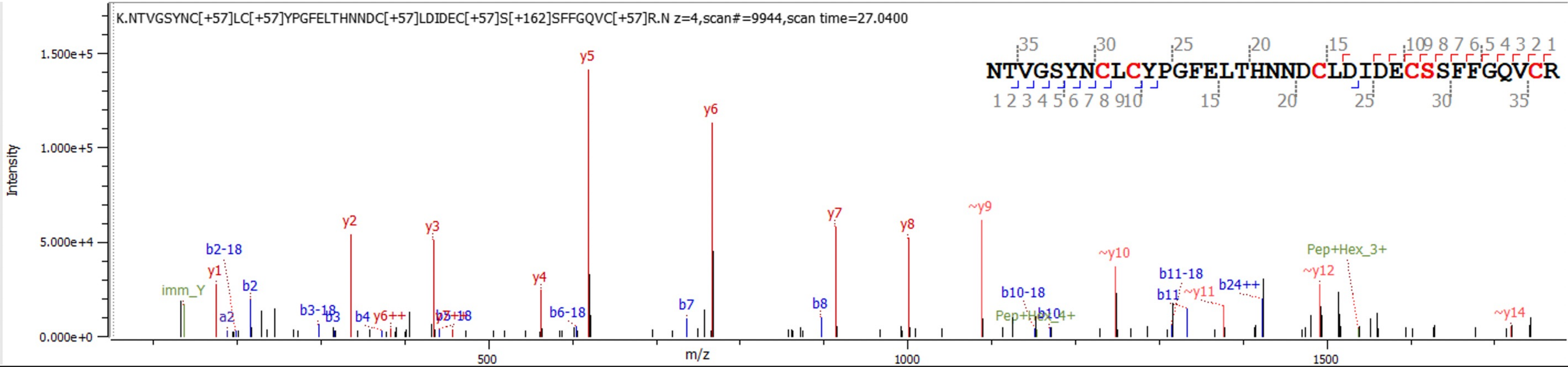

Figure S49
FBN2 EGF33

CGNEIGSFK

● Hexose/Glucose

#	a obs.	b obs.	b-18 obs.	Seq.	y obs.	#
1		161.0376		C		9
2	280.1111	308.1046		F		8
3	394.1494	422.1485		N	956.4503	7
4	523.1996	551.1863	533.1832	E	842.4069	6
5				I	713.3707	5
6				G	600.295	4
7				S-Hex(1)		3
8				F	294.1813	2
9				K	147.1127	1

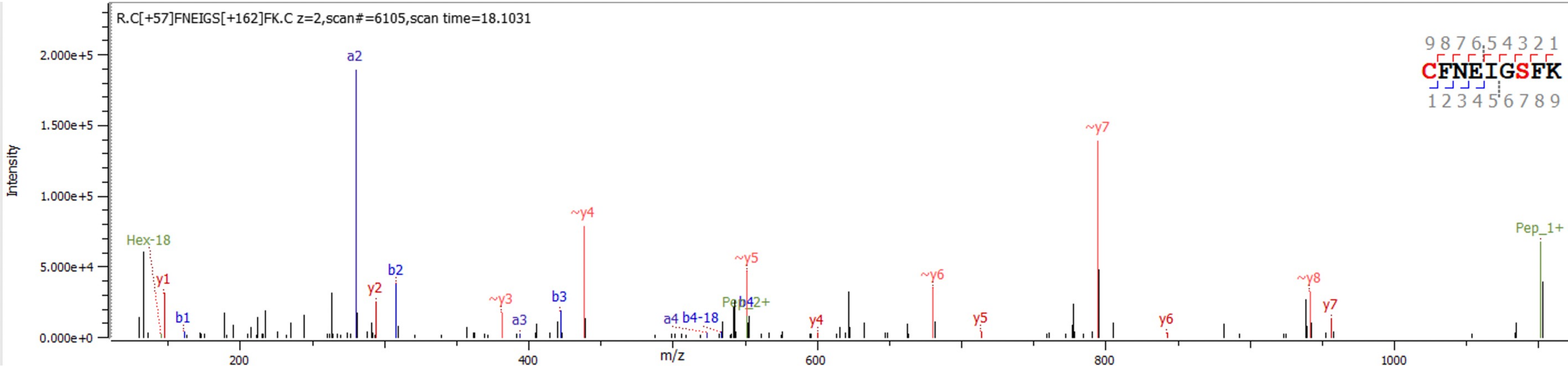

Figure S50
FBN2 EGF36

EDVNECLESPGICSNGQCINTDGSFR (S underlined, marked with ●)

● Hexose/Glucose

#	a obs.	b obs.	b-18 obs.	Seq.	y obs.	y++ obs.	#
1				E			26
2	217.0818	245.076	227.0654	D			25
3		344.144	326.1346	V			24
4		458.1881	440.1779	N			23
5	559.235	587.2289	569.2197	E			22
6		747.2601	729.2528	C			21
7	832.3555	860.3383	842.3405	L			20
8		989.3845	971.3808	E			19
9		1076.4214	1058.418	S			18
10				P		1022.9371	17
11				G			16
12				I			15
13				C	1777.698		14
14				S	1617.6769		13
15				N	1530.6422		12
16				G	1416.5984		11
17				Q			10
18				C	1231.5186		9
19				I	1071.4879		8
20				N	958.4116		7
21				T	844.365		6
22				D	743.3196		5
23				G	628.2969		4
24				S-Hex(1)	571.2721		3
25				F	322.1861		2
26				R	175.1187		1

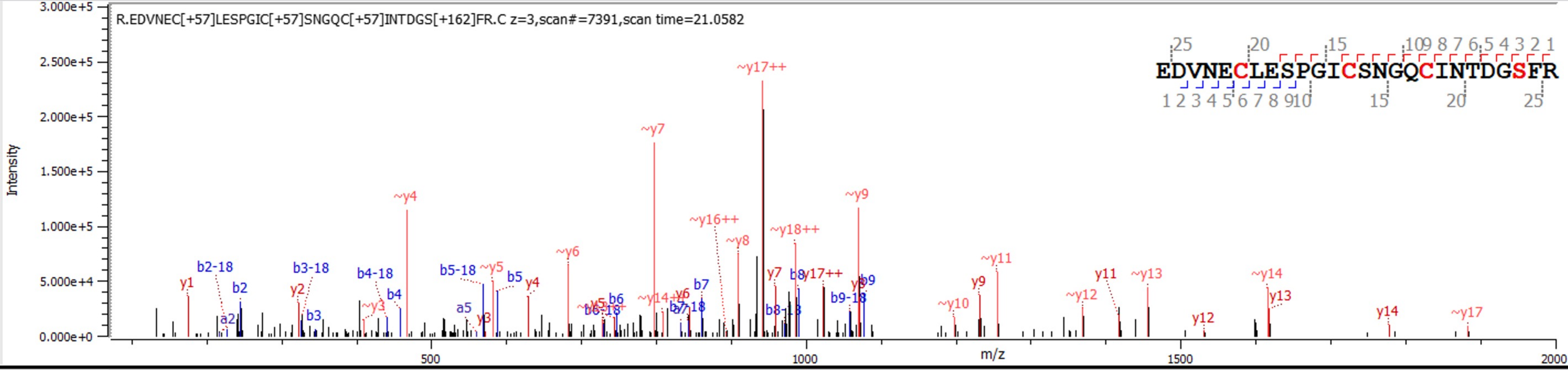

Figure S51
FBN2 EGF38

CMNTFGSYECTCPIGYALREDQK

● Hexose/Glucose

*Hexose is assigned to wrong residue. This is not uncommon to find with certain peptides. Double knockout data confirmed this modification is being added by POGLUT2 and 3.

#	a obs.	b obs.	b-18 obs.	Seq.	y obs.	y++ obs.	#
1		161.0371		C			23
2	264.0829	292.0774		M			22
3	378.1264	406.1198		N			21
4	479.1709	507.1677	489.1562	T			20
5	626.2391	654.2395	636.2275	F		1228.5378	19
6		711.2521		G		1155.0095	18
7			780.2864	S			17
8		961.3509		Y			16
9				E			15
10				C			14
11				T-Hex(1)			13
12				C	1449.7183	725.3682	12
13				P	1289.6827	645.3452	11
14				I			10
15				G	1079.549	540.2797	9
16				Y	1022.528		8
17				A	859.4651	430.2377	7
18				L	788.4254		6
19				R	675.3383		5
20				E	519.2405		4
21				D	390.1985		3
22				Q	275.1707		2
23				K	147.1119		1

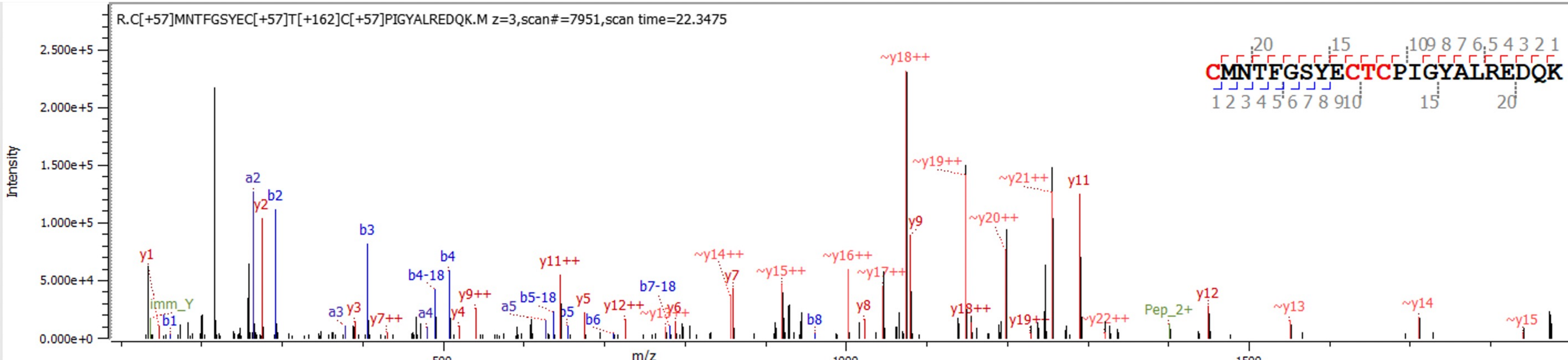

Figure S52
FBN2 EGF40

CVNIIGSYR

Hexose/Glucose

#	a obs.	b obs.	Seq.	y obs.	#
1		161.0372	C		9
2	232.1111	260.1059	V		8
3	346.1544	374.1485	N	984.4982	7
4	459.2385	487.2324	I	870.4556	6
5	572.3238	600.3127	I	757.3721	5
6			G	644.288	4
7			S-Hex(1)	587.2675	3
8			Y	338.1826	2
9			R	175.119	1

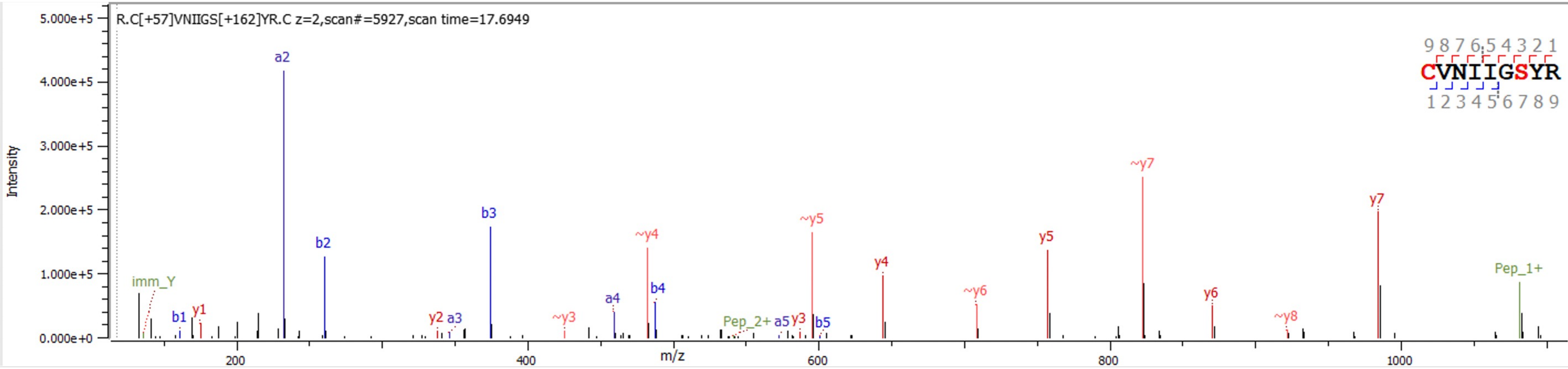

Figure S53
FBN2 EGF41

VMPNLCTNGQCINTMGSFR

Hexose/Glucose

#	a obs.	b obs.	Seq.	y obs.	#
1			V		19
2	203.121	231.1158	M		18
3			P		17
4		442.2117	N		16
5	527.3027	555.296	L		15
6		715.325	C		14
7			T		13
8	902.4132	930.4182	N		12
9			G		11
10		1115.5005	Q		10
11		1275.5135	C	1247.5397	9
12			I	1087.4888	8
13			N	974.4224	7
14			T	860.3857	6
15			M	759.3316	5
16			G	628.2935	4
17			S-Hex(1)		3
18			F	322.1888	2
19			R	175.1189	1

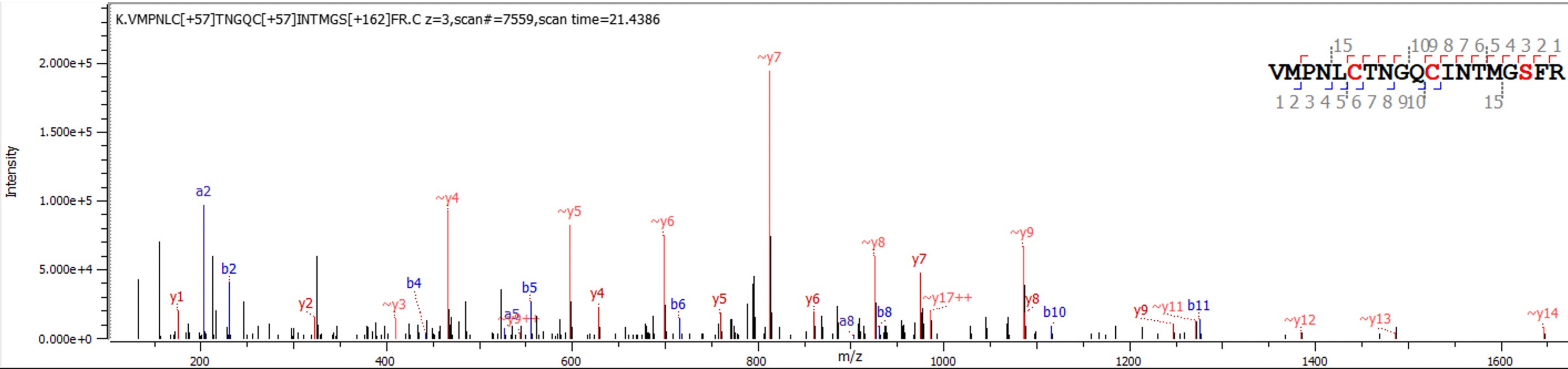

Figure S54
FBN2 EGF42

NTEGSYQCSCPR (Hexose on S)

● Hexose/Glucose

#	a obs.	b obs.	b-18 obs.	Seq.	y obs.	y++ obs.	#
1				N			12
2	188.1025	216.0974	198.0868	T			11
3		345.141	327.1292	E	1405.5387		10
4		402.1619	384.1516	G	1276.4911		9
5				S-Hex(1)	1219.4718		8
6				Y	970.3851	485.6966	7
7				Q	807.3223	404.1654	6
8				C	679.2639		5
9				S	519.2339		4
10				C	432.2017		3
11				P	272.171		2
12				R	175.1184		1

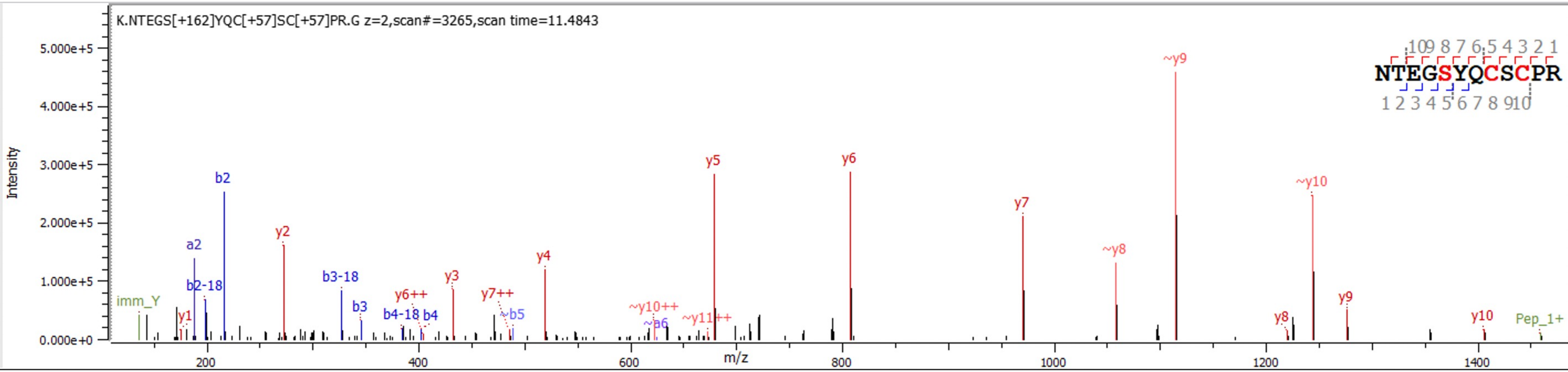

Figure S55
FBN2 EGF44

GICQNTPGSFSCECQR

● Hexose/Glucose

#	a obs.	b obs.	b-18 obs.	Seq.	y obs.	y++ obs.	#
1				G			16
2	143.1178	171.1127		I			15
3		331.1442		C			14
4		459.205		Q			13
5		573.2473		N			12
6		674.29	656.2819	T			11
7				P			10
8		828.3567		G			9
9				S-Hex(1)			8
10				F	986.3815	493.6949	7
11				S	839.3126	420.1601	6
12				C	752.2802		5
13				E	592.2496		4
14				C	463.2079		3
15				Q	303.1771		2
16				R	175.1189		1

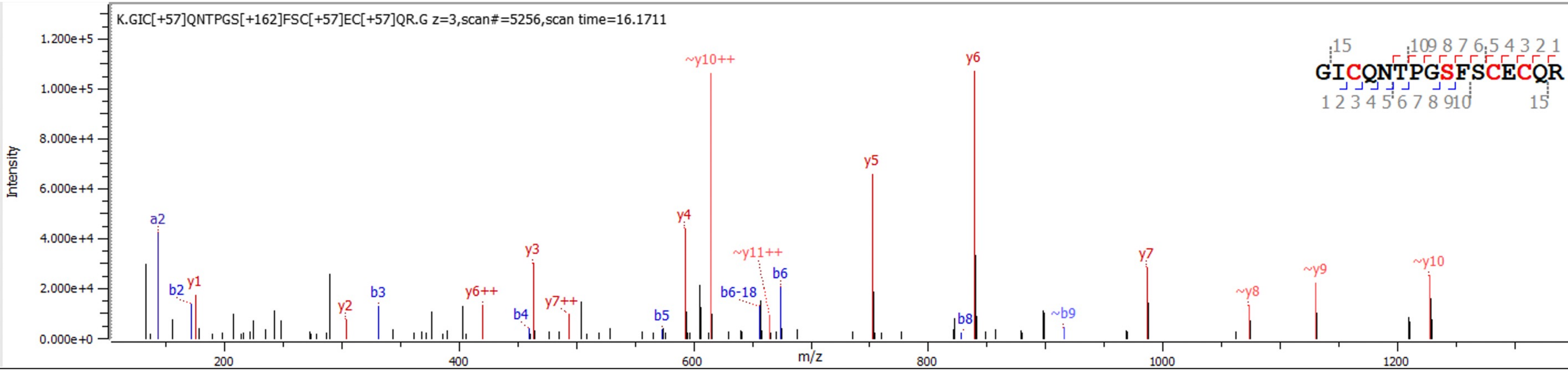

Figure S56
FBN2 EGF46

CGCPQGYIQHYQWNQCVDENECSNPNACGSASCYNTLGSYK

● Hexose/Glucose

#	a obs.	b obs.	b-18 obs.	b++ obs.	Seq.	y obs.	#
1					C		41
2		218.0591			G		40
3	350.0965	378.0894			C		39
4					P		38
5		603.2025			Q		37
6					G		36
7		823.2914			Y		35
8	908.3788	936.3776			I		34
9					Q		33
10					H		32
11					Y		31
12		1492.5825		746.8101	Q		30
13		1678.6846		839.8477	W		29
14		1792.7249			N		28
15				960.9035	Q		27
16				1040.9171	C		26
17		2179.875			V		25
18					D		24
19					E		23
20					N		22
21				1334.0385	E		21
22					C		20
23					S		19
24				1514.5912	N		18
25					P		17
26					N		16
27					A		15
28					C	1729.6984	14
29					G	1569.6573	13
30					S		12
31					A		11
32					S	1354.5796	10
33					C		9
34					Y		8
35					N		7
36					T	830.4108	6
37					L		5
38					G	616.2817	4
39					S-Hex(1)	559.2732	3
40					Y	310.1757	2
41					K	147.1125	1

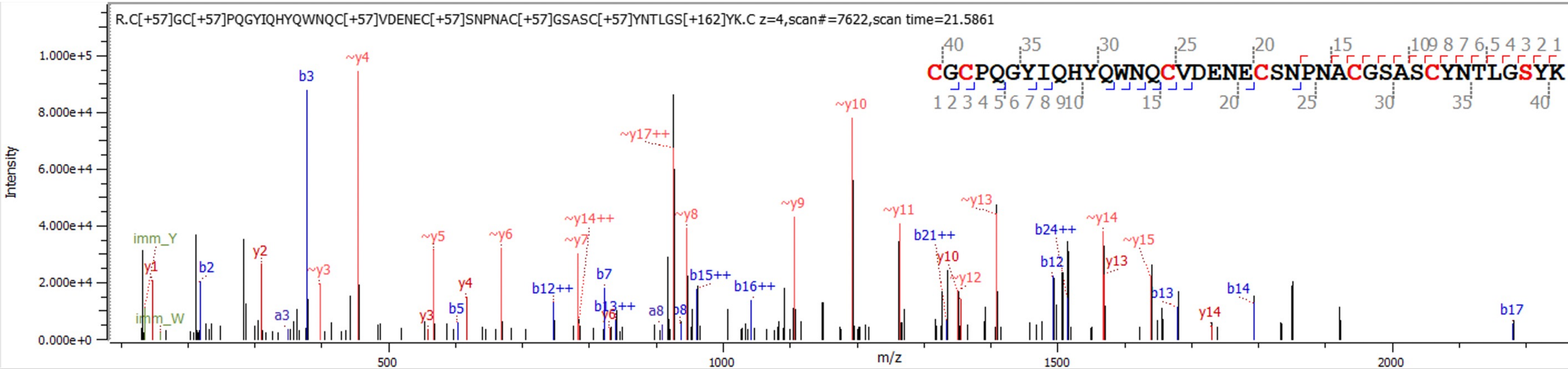

Figure S57
LTBP1 EGF3

CQLQGVCPNGECLNTMGSYR (S underlined; marked with a blue dot)

● Hexose/Glucose

#	a obs.	b obs.	b-18 obs.	Seq.	y obs.	#
1		161.0368		C		20
2	261.1001	289.0943	271.0839	Q		19
3	374.1813	402.1771	384.1674	L		18
4		530.2304	512.2258	Q		17
5	559.2681		569.2453	G	1976.808	16
6	658.3312	686.3276	668.3137	V	1919.7625	15
7	818.3647	846.3518	828.358	C	1820.7069	14
8				P	1660.674	13
9				N	1563.6324	12
10				G	1449.5865	11
11				E	1392.5858	10
12				C	1263.5255	9
13			1498.6293	L	1103.4851	8
14				N	990.4152	7
15				T	876.3672	6
16				M	775.322	5
17				G	644.2831	4
18				S-Hex(1)	587.2591	3
19				Y	338.1801	2
20				R	175.1177	1

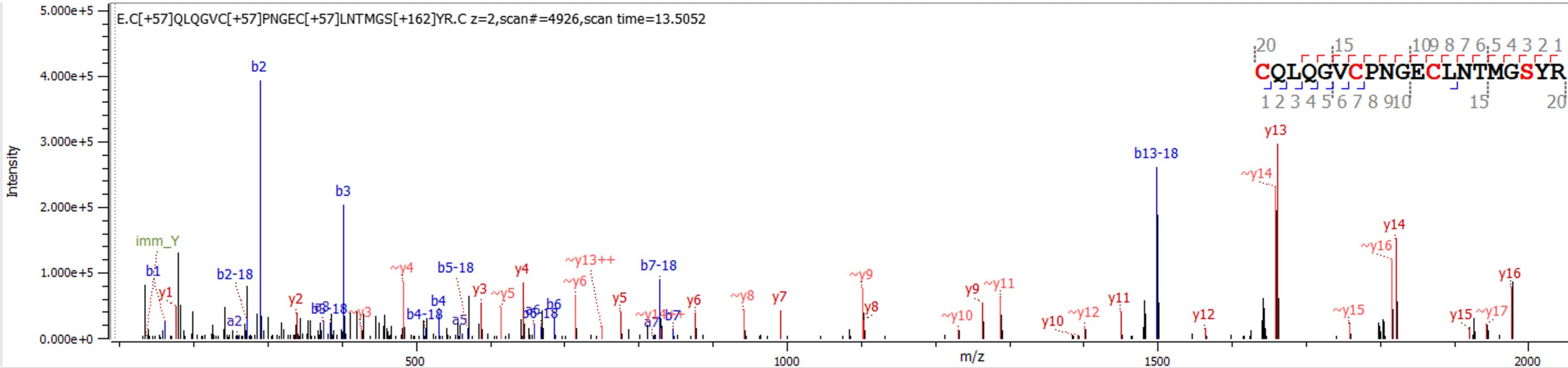

Figure S58
LTBP1 EGF5

GSFLCICPAGFMASEE

● Hexose/Glucose

*Hexose is assigned to wrong residue. This is not uncommon to find with certain peptides. Double knockout data confirmed this modification is being added by POGLUT2 and 3.

#	a obs.	b obs.	b-18 obs.	Seq.	y obs.	#
1				G		16
2		145.0592		S		15
3	264.1325	292.1272	274.1172	F		14
4	377.215	405.2097	387.1997	L		13
5	537.2458	565.239	547.2243	C		12
6	650.3307	678.3251	660.3127	I		11
7	810.3618	838.3511	820.338	C		10
8				P		9
9		1006.4479	988.4263	A		8
10		1063.4552	1045.4572	G		7
11		1210.5261	1192.5236	F		6
12				M		5
13				A		4
14				S-Hex(1)		3
15				E	277.1012	2
16				E	148.0594	1

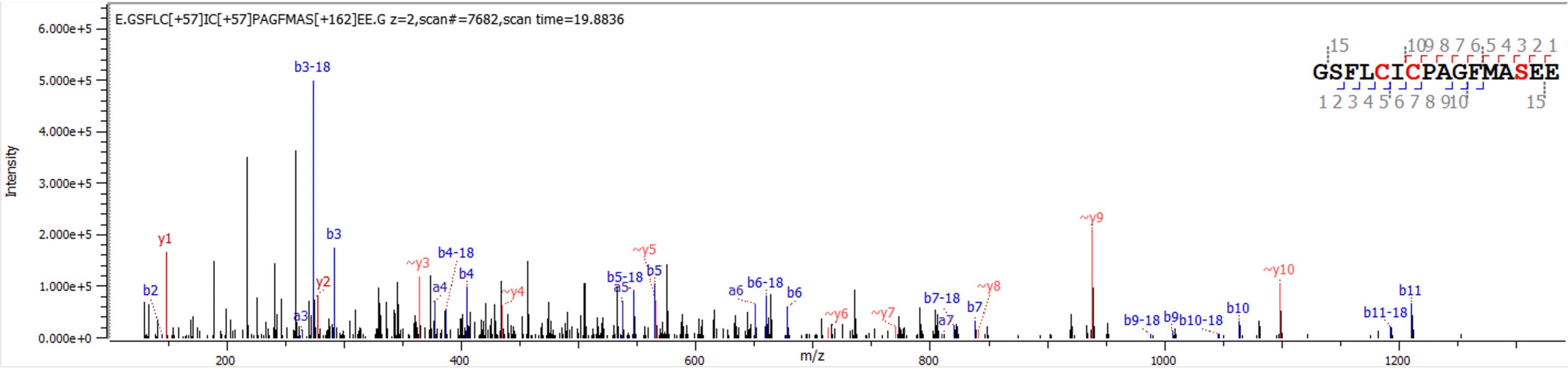

Figure S59
LTBP1 EGF7

GRCEDIDECLNPSTCPDEQCNSPG<u>S</u>YQCVPCTEGFR

● Hexose/Glucose

#	a obs.	b obs.	b-18 obs.	b++ obs.	Seq.	y obs.	y++ obs.	#
1					G			37
2		214.1265			R			36
3	346.1593	374.1522			C			35
4	475.2056	503.2028			E			34
5		618.2261			D			33
6	703.3116	731.3099			I			32
7		846.3365	828.3231		D			31
8	947.3884	975.3789	957.3687		E			30
9	1107.4242	1135.4087	1117.4048		C			29
10	1220.4972	1248.4928	1230.4801		L			28
11	1334.5399	1362.5355	1344.5262	681.765	N			27
12		1459.5962			P		1597.6527	26
13		1546.6226	1528.5963		S			25
14	1619.6757	1647.6681	1629.6636		T			24
15	1779.7014	1807.6957	1789.6925		C			23
16					P		1375.0421	22
17					D			21
18		2148.8323			E	2536.9648		20
19		2276.8655			Q			19
20				1218.9528	C	2279.8987		18
21				1268.5112	V	2119.8728		17
22		2650.0271		1325.5125	N	2020.8043		16
23		2737.0381	2719.032	1369.0316	S	1906.7858		15
24					P	1819.7521	910.378	14
25					G	1722.6852		13
26					S-Hex(1)	1665.679		12
27					Y	1416.5948		11
28					Q	1253.5337		10
29					C	1125.476		9
30					V	965.4458		8
31					P	866.3778	433.6931	7
32					C	769.326		6
33					T	609.2957		5
34					E	508.2491		4
35					G	379.2062		3
36					F	322.1851		2
37					R	175.118		1

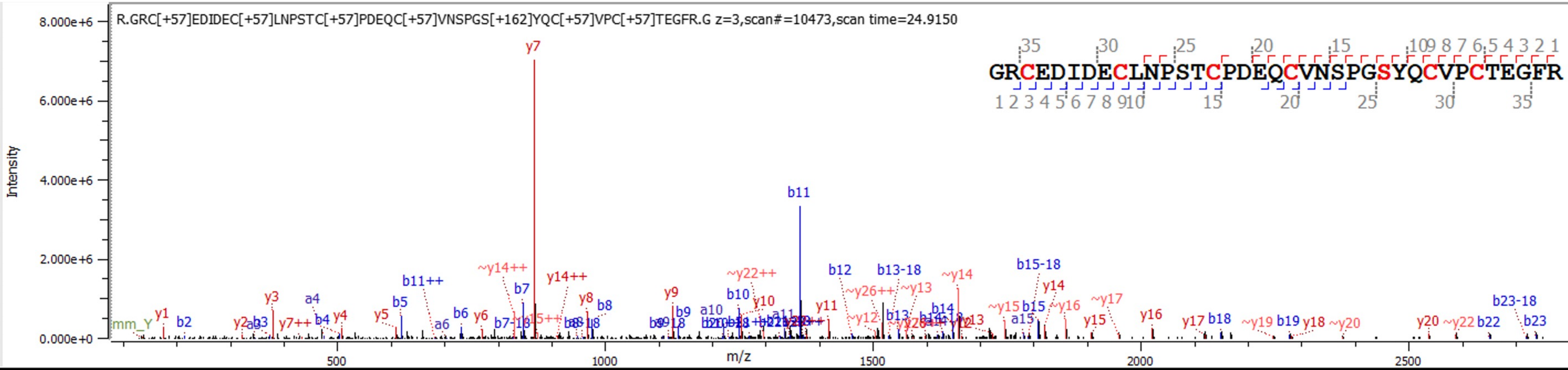

Figure S60
LTBP1 EGF8

GWNGQCLDVDECLEPNVCANGDCSNLEGSYMCSCHK

Hexose/Glucose

#	a obs.	b obs.	b-18 obs.	Seq.	y obs.	y++ obs.	#
1				G			36
2		244.1073		W			35
3		358.1494		N			34
4		415.1696		G		2020.7377	33
5		543.2311		Q			32
6		703.2612		C			31
7	788.3312			L			30
8		931.3701		D			29
9		1030.4381		V			28
10				D			27
11				E			26
12		1434.5417	1416.5511	C			25
13		1547.6023		L			24
14		1676.6643	1658.6598	E			23
15				P		1361.5193	22
16				N			21
17				V			20
18				C			19
19				A	2251.8022	1126.408	18
20				N	2180.7617		17
21				G			16
22				D			15
23				C	1894.7002		14
24				S	1734.6573		13
25				N			12
26				L			11
27				E	1420.5052		10
28				G			9
29				S-Hex(1)			8
30				Y	985.3658		7
31				M	822.3027		6
32				C	691.2614		5
33				S	531.2316		4
34				C	444.202		3
35				H			2
36				K			1

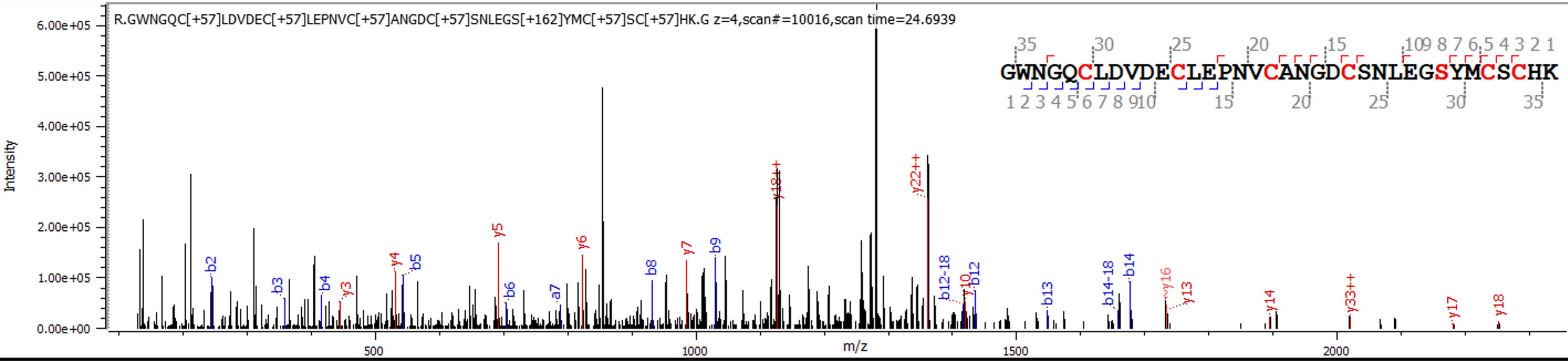

Figure S61
LTBP1 EGF10

NTEG<u>S</u>FQCVCDQGYR

● Hexose/Glucose

#	a obs.	b obs.	b-18 obs.	Seq.	y obs.	#
1				N		15
2	188.1017	216.0963	198.0858	T		14
3		345.1397	327.127	E		13
4		402.1638	384.1486	G	1638.6317	12
5				S-Hex(1)		11
6			780.3003	F	1332.5355	10
7	898.3817			Q	1185.4684	9
8				C	1057.4105	8
9			1167.4524	V	897.381	7
10				C	798.3137	6
11				D	638.2845	5
12				Q	523.2585	4
13				G	395.2007	3
14				Y	338.1794	2
15				R	175.1177	1

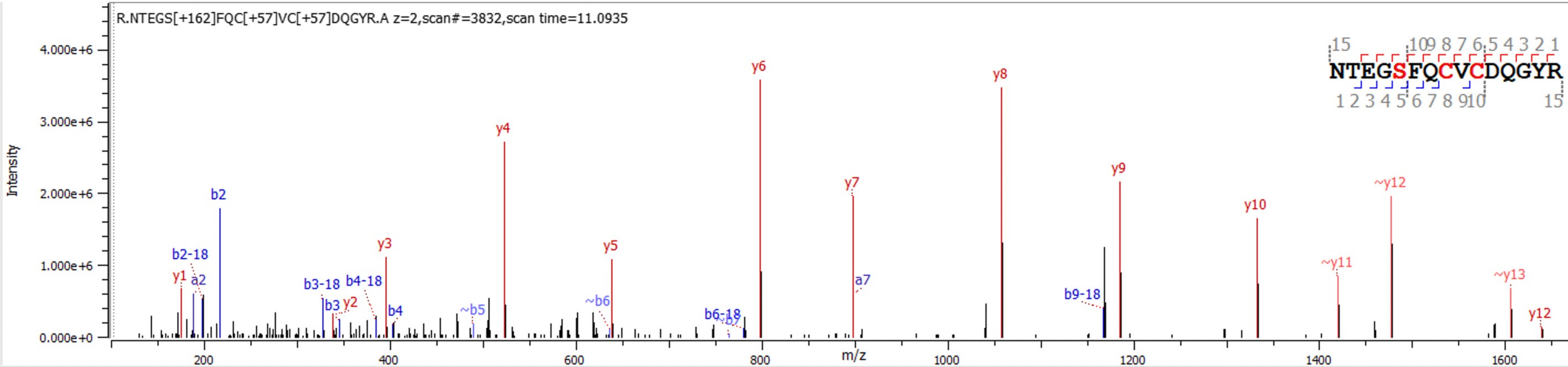

Figure S62
LTBP1 EGF12

GSFHCVCQQGFSISADGR

● Hexose/Glucose

#	b obs.	Seq.	y obs.	y++ obs.	#
1		G			18
2		S-Hex(1)			17
3		F			16
4		H		861.3668	15
5		C	1584.6836		14
6		V	1424.6503		13
7		C	1325.5784		12
8		Q	1165.5514		11
9		Q	1037.4921		10
10		G	909.4349		9
11		F	852.4139		8
12		S	705.3452		7
13		I	618.32		6
14		S	505.2314		5
15		A	418.201		4
16		D	347.1648		3
17		G	232.1386		2
18		R	175.1176		1

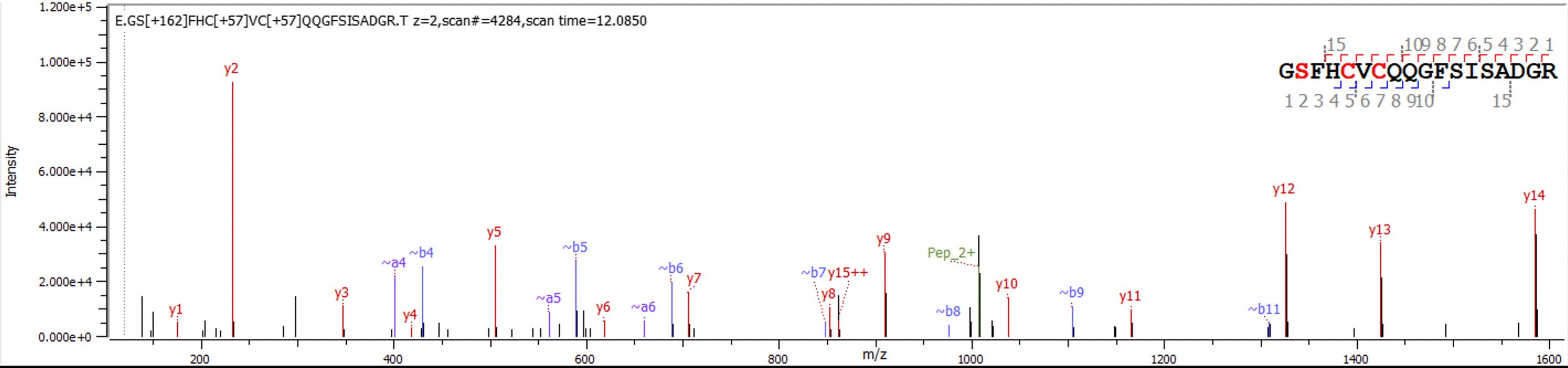

Figure S63
LTBP1 EGF16

GSYNCFCTHPMVLDASEK

Hexose/Glucose

#	b obs.	b++ obs.	Seq.	y obs.	y++ obs.	#
1			G			18
2	348.1399		S-Hex(1)			17
3			Y			16
4			N-Oxidation			15
5			C		847.8654	14
6			F	1534.6899	767.8508	13
7			C	1387.6276	694.3176	12
8			T	1227.5955		11
9			H	1126.5414	563.7773	10
10			P	989.4904	495.2477	9
11			M	892.4417		8
12			V	761.3991		7
13			L	662.3296		6
14			D	549.2477		5
15		986.9194	A	434.2204		4
16			S	363.1844		3
17			E	276.1532		2
18			K	147.1118		1

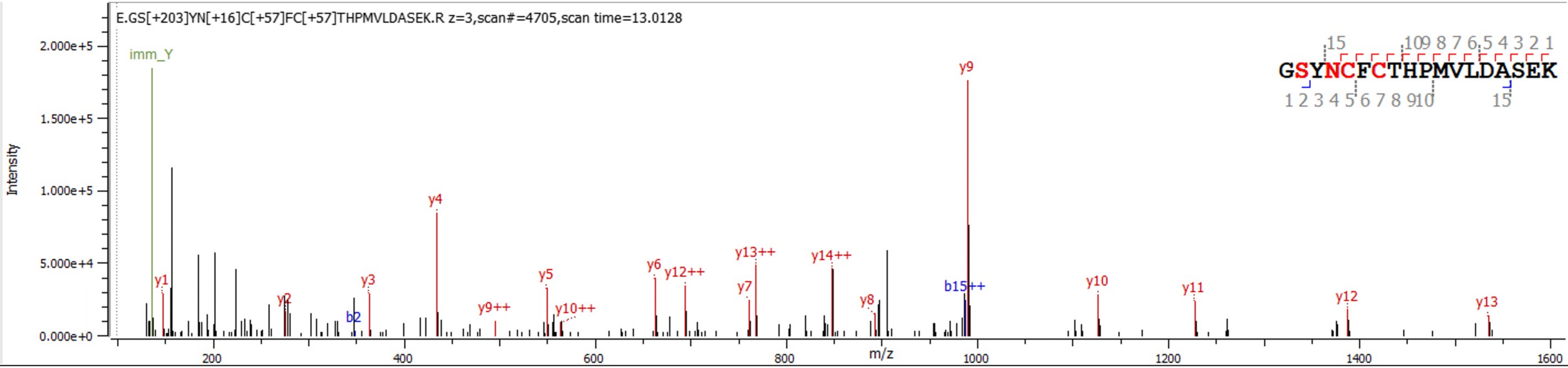

Figure S64
LTBP1 EGF18

CINTDGSYK

Hexose/Glucose

#	a obs.	b obs.	b-18 obs.	Seq.	y obs.	#
1		161.0368		C		9
2	246.1254	274.1202		I		8
3		388.162		N	946.3942	7
4		489.2102		T	832.3514	6
5		604.2361	586.2297	D	731.2941	5
6				G	616.2803	4
7				S-Hex(1)		3
8				Y	310.1748	2
9				K	147.112	1

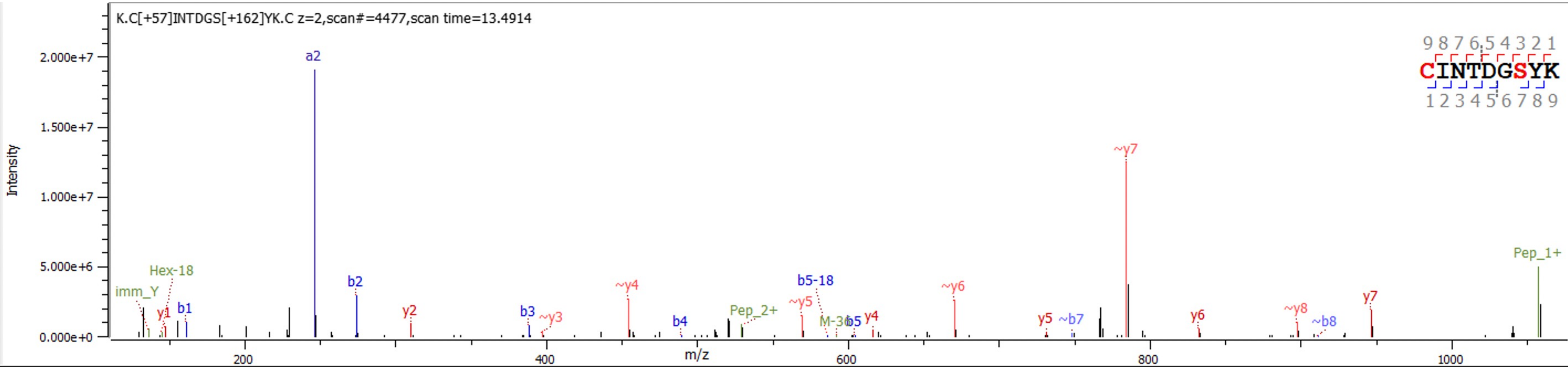

Supplement: Supplemental Figures S1–S64 and Tables S1–S3 [file mmc1.pdf]
